# Supplementary material for: ATP13A4 gates extracellular polyamine levels to control excitatory synaptogenesis
Source: Nat Commun. 2026 Aug 4;17:9395. doi: 10.1038/s41467-026-76132-z (PMC13534584; doi:10.1038/s41467-026-76132-z)
Supplement: Supplementary file 1 [file 41467_2026_76132_MOESM1_ESM.pdf]

Supplementary Information

Supplementary Figures and Legends

Supplementary Tables

Supplementary References

Uncropped blots and autoradiograms for Supplementary Figures

Supplementary Figures and Legends

Supplementary Fig. 1: hATP13A4 is enriched in astrocytes and oligodendrocyte precursor cells in the human brain.

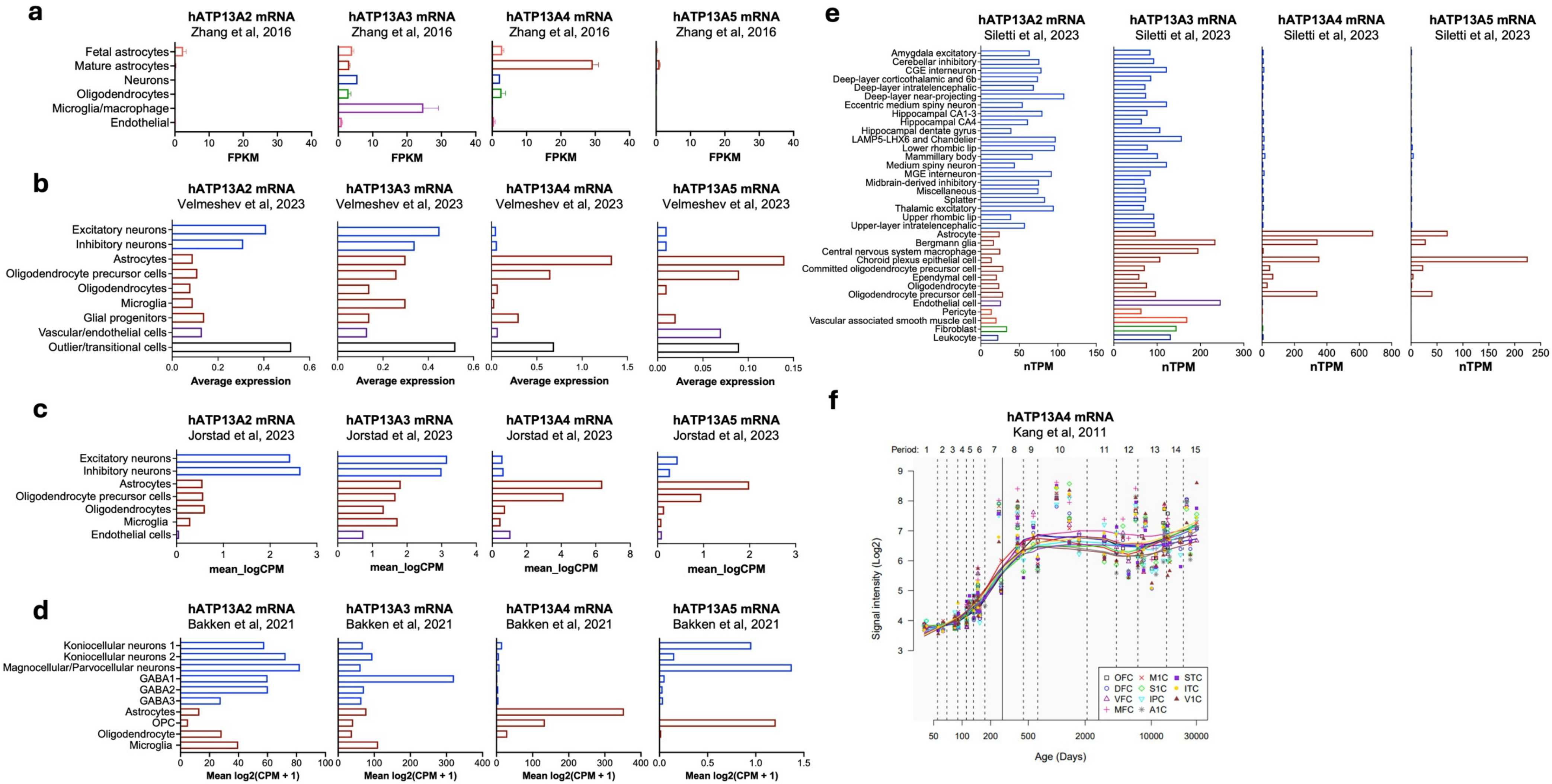

Supplementary Fig. 1: hATP13A4 is enriched in astrocytes and oligodendrocyte precursor cells in the human brain. Analysis of *hATP13A2-5* mRNA expression across major human brain cell types using publicly available transcriptomic datasets. **a**, Bulk RNA-seq from purified human brain cell populations (1), shown as FPKM (Fragments Per Kilobase Million), demonstrating strong enrichment of *hATP13A4* in astrocytes, with higher expression in mature compared to fetal astrocytes; **b-e**, Single-cell and single-nucleus RNA-seq datasets from human prenatal and postnatal cortex (2), neocortex (3), dorsal lateral geniculate nucleus (4), and adult human brain (5), shown as CPM (counts per million) or nTPM (normalized transcript-per-million) as indicated. Across datasets, *hATP13A4* expression is predominant in astrocytes, with lower expression in oligodendrocyte precursor cells (OPCs) and additional enrichment in specialized glial populations such as Bergmann glia and choroid plexus epithelial cells, while neuronal and microglial expression remains low to undetectable. **f**, *hATP13A4* expression across human cortical regions (6) (Human Brain Transcriptome Atlas; http://hbatlas.org/), showing increased expression during late fetal and early postnatal development, consistent with potential developmental regulation. OFC, orbital prefrontal cortex; DFC, dorsolateral prefrontal cortex; VFC, ventrolateral prefrontal cortex; MFC, medial prefrontal cortex; M1C, primary motor cortex; S1C, primary somatosensory cortex; IPC, posterior inferior parietal cortex; A1C, primary auditory cortex; STC, posterior superior temporal cortex; ITC, inferior temporal cortex; V1C, primary visual cortex. Source data are provided as a Source Data file.

Supplementary Fig. 2: msAtp13a4 is predominantly expressed in astrocytes in the mouse brain.

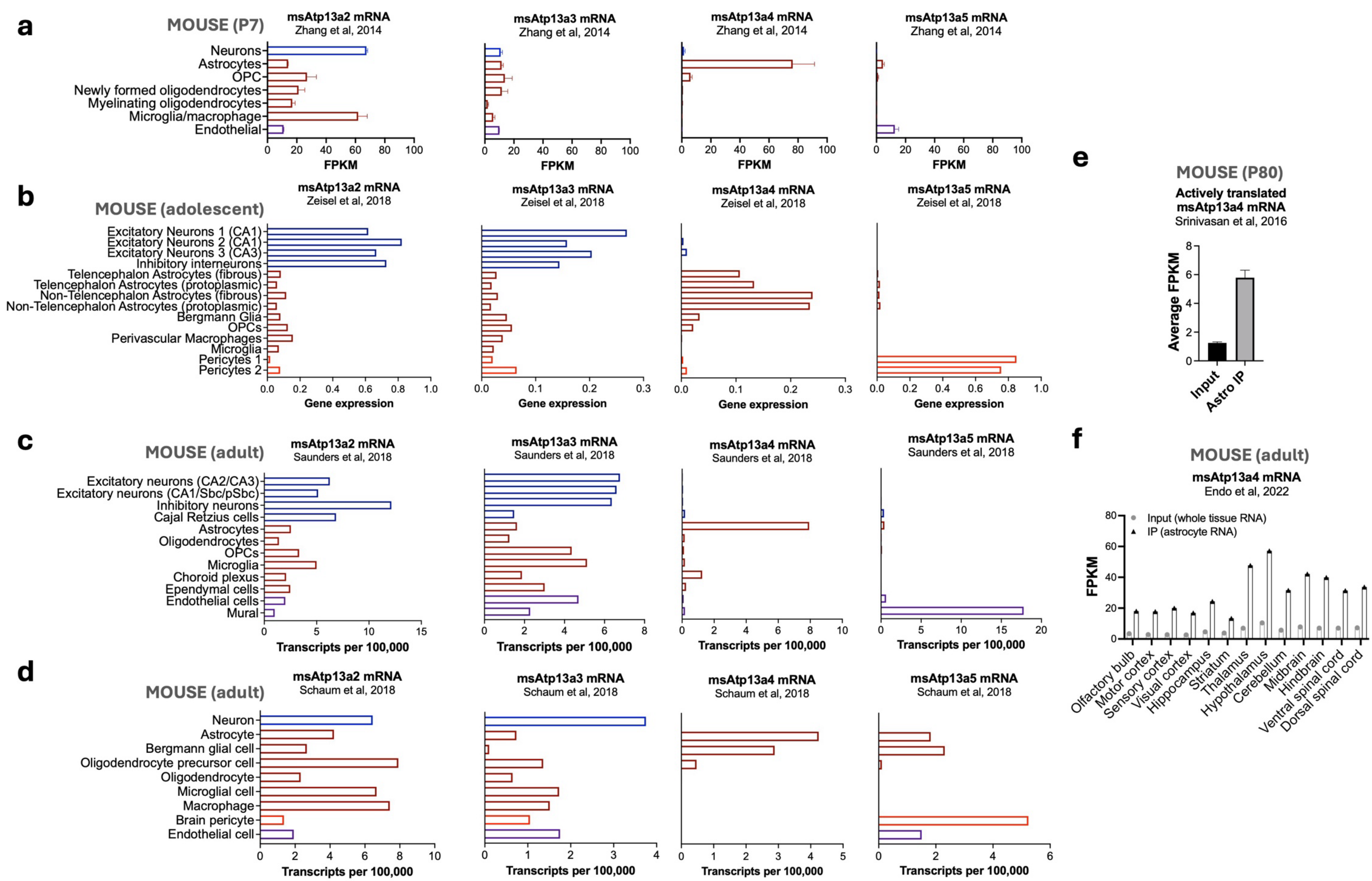

Supplementary Fig. 2: msAtp13a4 is predominantly expressed in astrocytes in the mouse brain. Analysis of *msAtp13a2-5* mRNA levels across major mouse brain cell types and developmental stages using publicly available transcriptomic datasets. **a-d**, Bulk and single-cell RNA-seq datasets from mouse cortex and brain, including purified cortical cell populations at postnatal day 7 (P7) (7), adolescent brain (8), adult brain (9), and the Tabula Muris dataset (10), demonstrating strong and selective enrichment of *msAtp13a4* in astrocytes, with negligible expression in neurons and microglia and minimal expression in oligodendroglial lineages. **e**, Ribosome profiling (Astro-IP) showing active translation of *msAtp13a4* mRNA in P80 mouse astrocytes compared to bulk tissue RNA (input) (11). **f**, Region-specific astrocyte RNA-seq datasets showing consistent enrichment of *msAtp13a4* across 13 CNS regions in adult mice (12), identifying msAtp13a4 as part of a core astrocyte-enriched gene set. Source data are provided as a Source Data file.

Supplementary Fig. 3: Atp13a4 localizes to late endo-/lysosomal compartments.

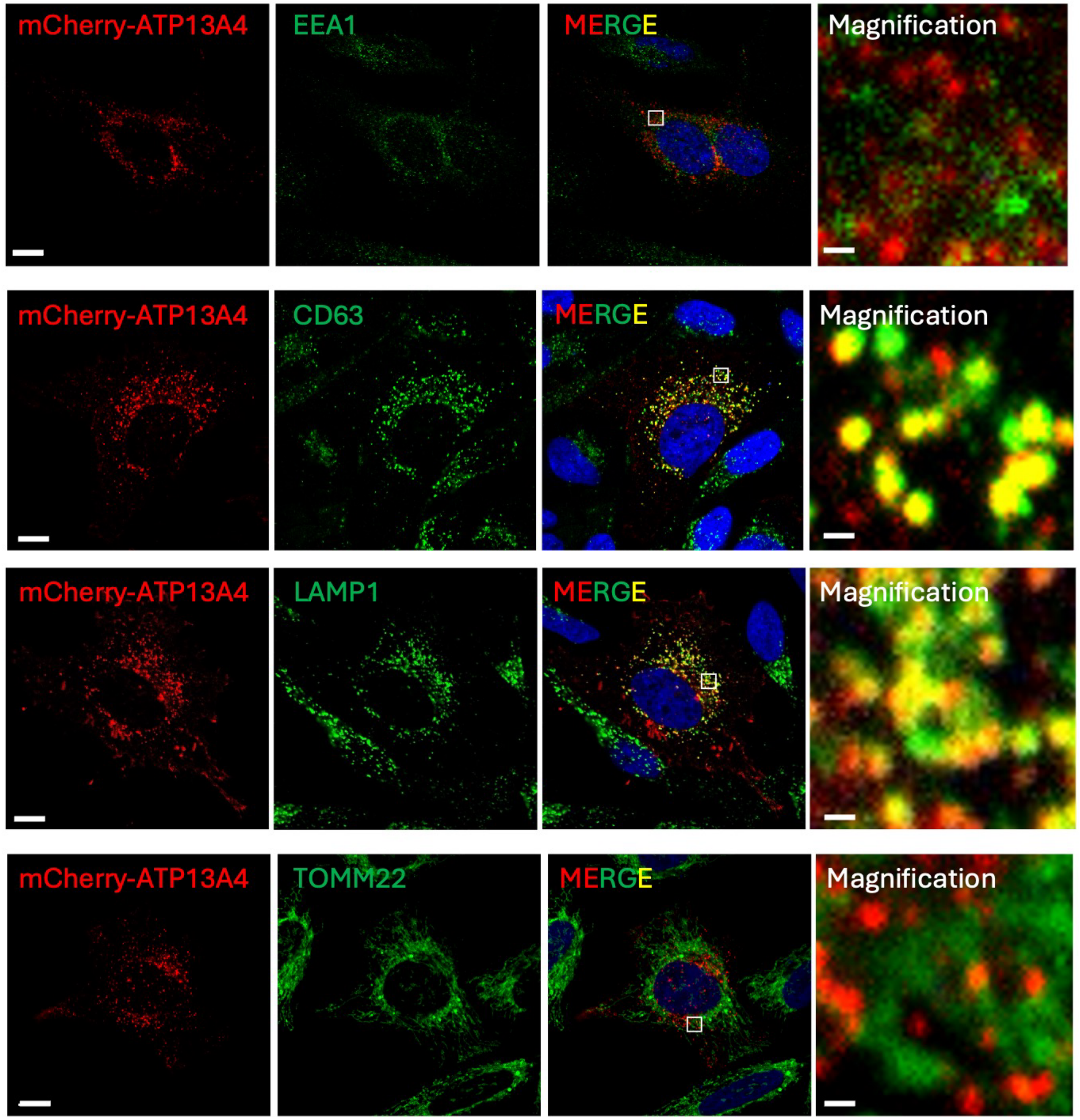

Supplementary Fig. 3: Atp13a4 localizes to late endo-/lysosomal compartments. Immunocytochemistry of HeLa cells transiently expressing N-terminal mCherry-labeled mouse Atp13a4 (msAtp13a4), co-stained with markers for specific organelles: EEA1 (early endosomes), CD63 (late endosomes), LAMP1 (lysosomes), and TOMM22 (mitochondria). Scale bar, 10 µm. Inset shows a magnification of the indicated white rectangular region (scale bar, 625 nm). *n* = 41 (CD63), 49 (EEA1), 51 (LAMP1), 41 (TOMM22) cells from three independent experiments.

Supplementary Fig. 4: TurboID construct expression and biotinylation *in vivo.*

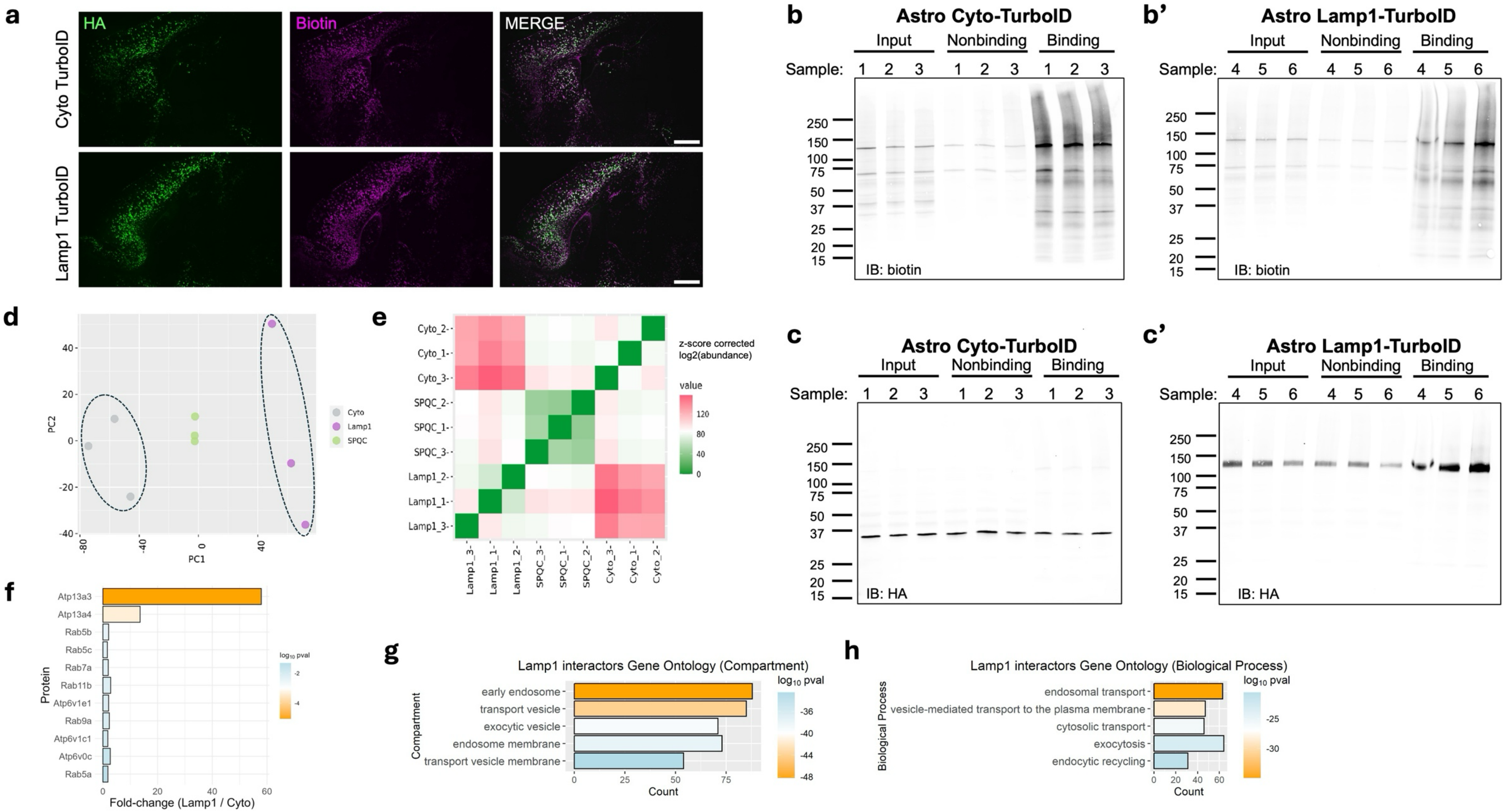

Supplementary Fig. 4: TurboID construct expression and biotinylation *in vivo.* **a**, Representative images of *in vivo* expression in the cortex of different TurboID constructs labeled with HA and the biotinylating activity labeled with streptavidin. Merged images show the colocalization of HA and biotin signals in astrocytes. Scale bar, 1000 µm. **b**-**c**, HA immunoblotting verifies expression of Astro-Cyto-TurboID (**b**) and Astro-Lamp1-TurboID (**b'**). Streptavidin blotting verifies biotinylation activity of Astro-Cyto-TurboID (**c**) and Astro-Lamp1-TurboID (**c'**) in cortical lysates (input) and subsequent immunoprecipitation (binding). **d**, Principal component analysis (PCA) of TurboID data shows Astro-Lamp1-TurboID samples (purple) clustering separately from Astro-Cyto-TurboD samples (gray) and study pool QC (SPQC) samples (green). **e**, Heatmap displaying the correlation of protein abundance for all pairwise combinations of samples. Unsupervised hierarchical clustering shows Astro-Lamp1-TurboID samples clustering separately from Astro-Cyto-TurboID and SPQC samples. **f**, Bar graph depicting the detection ratio of Lamp1-TurboID versus Astro-Cyto-TurboID for putative polyamine transporters (Atp13a3 and Atp13a4) and selected endo-lysosomal proteins. (**g**-**h**) Bars show the top 10 most significant Gene Ontology (GO) terms, ordered by lowest adjusted p-value, for the proteins differentially detected by Astro-Lamp1-TurboID compared to Astro-Cyto-TurboID. (**g**) Cellular Compartment; (**h**) Biological Process.

Supplementary Fig. 5: hATP13A4 spontaneously forms a phospho-enzyme that is sensitive to polyamines.

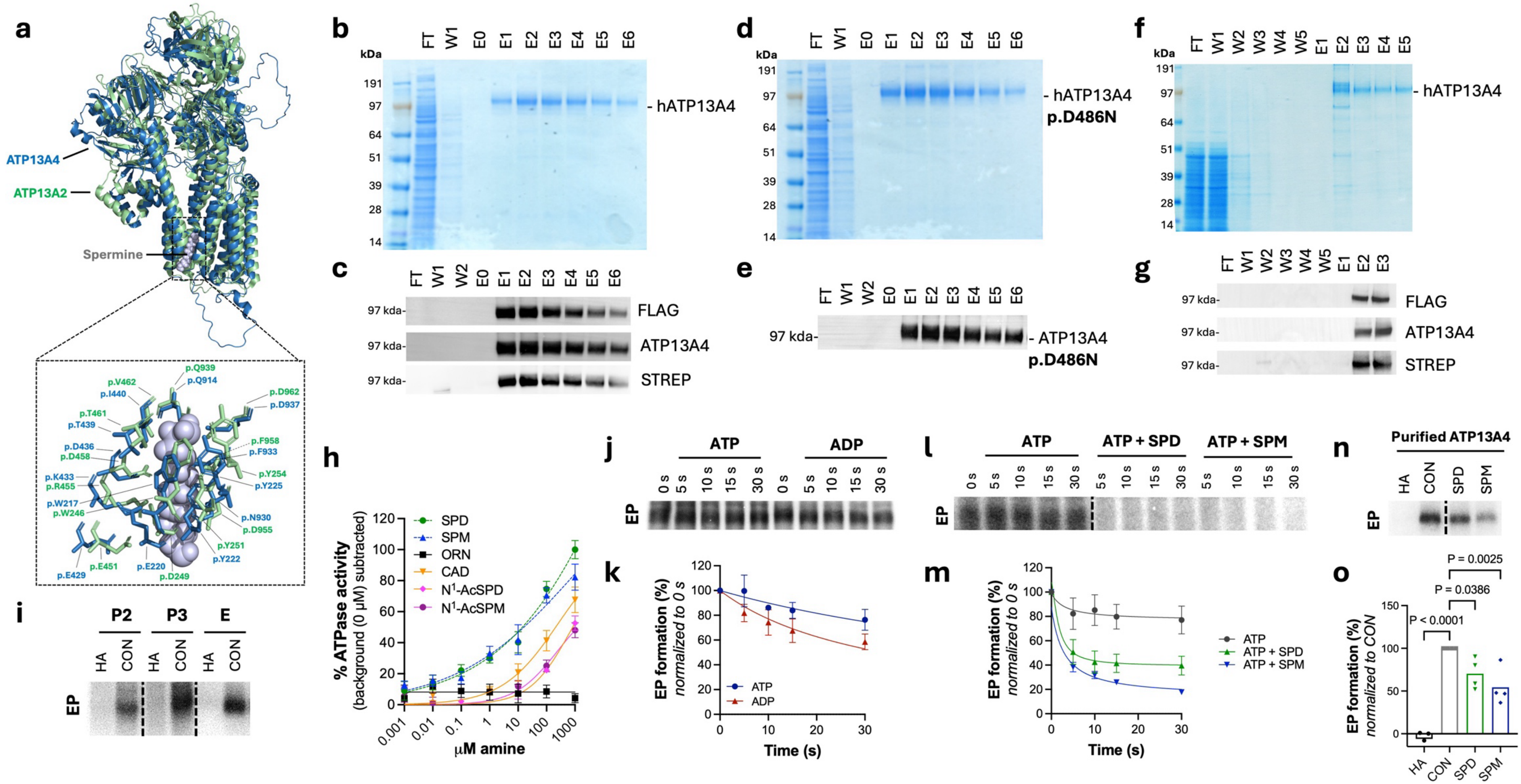

Supplementary Fig. 5: hATP13A4 spontaneously forms a phospho-enzyme that is sensitive to polyamines. a, Structural visualization of hATP13A4 (AlphaFold prediction) and hATP13A2 (PDB 7n78) using PyMOL. Top: overlay of hATP13A4 (blue) and hATP13A2 (green), with spermine shown in grey. Bottom: close-up view of the spermine binding pocket. **b**-**g**, Coomassie staining (**b**, **d**, **f**) and Western blot analysis (**c**, **e**, **g**) showing the purification process for WT hATP13A4 (**b**-**c**, **f**-**g**) or the p.D486N mutant (**d**-**e**), starting from detergent-solubilized HEK293T cell lysates (**b**-**e**) or yeast membranes (**f**-**g**), followed by streptavidin affinity chromatography and subsequent elution with biotin. $n \geq 3$ independent purifications. FT, flow-through; W, wash; E, elution. **h**, Dose-response curves showing the effect of spermidine (SPD), ornithine (ORN), cadaverine (CAD), N^1-acetylspermidine (N^1-AcSPD) and N^1-acetylspermine (N^1-AcSPM) on the ATPase activity of solubilized purified ATP13A4 WT. A spermine (SPM) reference curve, derived from Fig. 2b, is included for comparison. Data are presented as mean ± SEM. $n = 4$ independent experiments for ORN, CAD, N^1-AcSPD, and N^1-AcSPM. For SPD, $n = 4$ at 1000 µM and $n = 2$ at the other concentrations. **i**, Representative autoradiogram showing phosphoenzyme (EP) formation of WT hATP13A4 in yeast membrane fractions (heavy membrane fraction P2 and light membrane fraction P3) and purified protein, which is sensitive to hydroxylamine (HA). **j**-**k**, Representative autoradiogram (**j**) and quantification (**k**) of pulse ([γ-^{32}P]-ATP) chase (cold ATP or ADP) experiment to determine ATP and ADP sensitivity of hATP13A4 EP. Data are presented as mean ± SEM. $n = 3$ independent experiments. **l**-**m**, Representative autoradiogram (**l**) and quantification (**m**) of pulse ([γ-^{32}P]-ATP) chase (cold ATP) experiment in the presence and absence of spermidine (SPD) or spermine (SPM) in yeast membranes. Data are presented as mean ± SEM. $n = 4$ (ATP + SPM) or 5 (ATP, ATP + SPD) independent experiments. **n**-**o**, Representative autoradiogram (**n**) and quantification (**o**) of purified ATP13A4 EP levels in the presence of 0.3 M HA, 1 mM SPD or SPM. $n = 4$ independent experiments. Data are presented as mean with individual data points. One-way ANOVA with Dunnett's post-test. Source data are provided as a Source Data file.

Supplementary Fig. 6: ATP13A4 promotes cellular polyamine uptake.

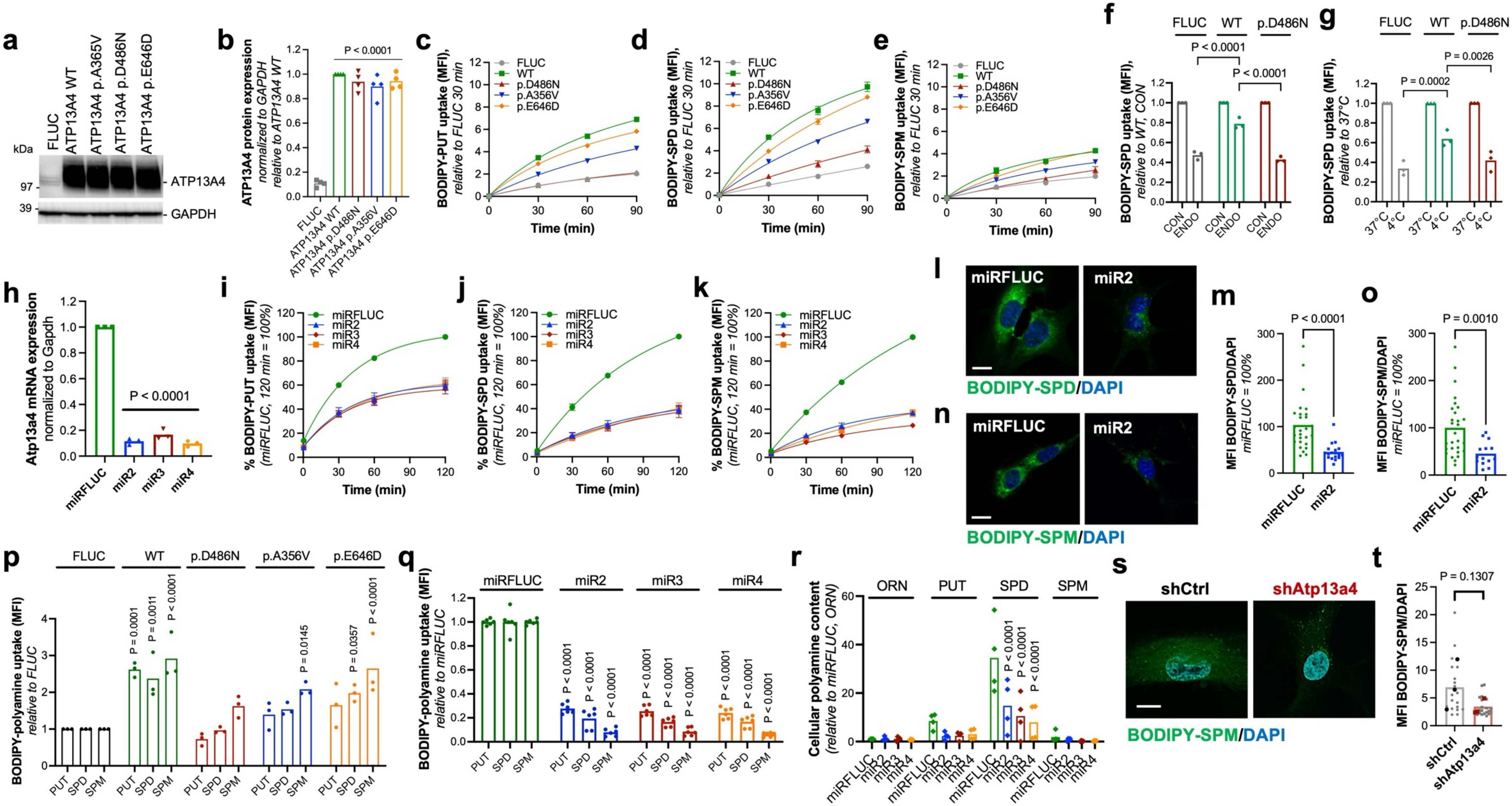

Supplementary Fig. 6: ATP13A4 promotes cellular polyamine uptake. **a**, Overexpression of firefly luciferase (FLUC), WT hATP13A4, the p.D486N mutant, or disease-associated variants p.A356V and p.E646D in H4 cells was confirmed by immunoblotting. GAPDH, loading control. **b**, Densitometric quantification of hATP13A4 expression shown in **a**. $n = 4$ independent cultures. One-way ANOVA with Dunnett's multiple comparisons test. **c-e**, Flow cytometric analysis of cellular BODIPY-putrescine (PUT, **c**), -spermidine (SPD, **d**) or -spermine (SPM, **e**) uptake in serum-containing medium. MFI, mean fluorescence intensity. $n = 3$ independent experiments. **f**, BODIPY-SPD uptake following treatment with a combination of endocytosis inhibitors (ENDO; Dynasore, Genistein, Pitstop 2). **g**, BODIPY-SPD uptake at 4 °C and 37 °C. **f**-**g**, $n = 3$ independent experiments. Two-way ANOVA with Šídák's post-test. **h**, *msAtp13a4* mRNA expression following knockdown with lentiviral shRNA transduction (miR2-4) was assessed by qPCR. $n = 3$ independent experiments. One-way ANOVA with Dunnett's post-test. **i-k**, BODIPY-PUT (**i**), -SPD (**j**) or -SPM (**k**) uptake in serum-containing medium. $n = 3$ independent experiments. **l-o**, Confocal microscopy analysis of BODIPY-SPD (**l**, **m**) and -SPM (**n**, **o**) distribution. Scale bar, 10 µm. MFI of BODIPY-SPD (**m**) and -SPM (**o**) corresponding to images in **l** (miRFLUC, 27 cells; miR2, 18 cells) and **n** (miRFLUC, 28 cells; miR2, 14 cells), respectively. $n = 3$ independent cultures. Two-tailed Mann-Whitney test. **p**-**q**, BODIPY-PUT, -SPD or -SPM uptake in H4 cells overexpressing FLUC, WT hATP13A4 or indicated variants (**p**) and in C8-D1A cells with *msAtp13a4* knockdown (**q**), performed in serum-free medium. $n = 3$ independent experiments. Two-way ANOVA with Šídák's post-test. **r**, Metabolomics of cellular polyamines in *msAtp13a4* knockdown cells (miR2-4) compared with C8-D1A control (miRFLUC). $n = 3$ independent experiments. Two-way ANOVA with Šídák's post-test. **s**, Confocal images of BODIPY-labelled SPM distribution in control (shCtrl) and *msAtp13a4* knockdown (shAtp13a4) primary astrocytes. Scale bar, 10 µm. **t**, MFI of BODIPY-SPM (shCtrl, 19 cells; shAtp13a4, 20 cells). $n = 3$ independent cultures. Individual data points shown (light grey), with experiment averages plotted in black (shCtrl) or red (shAtp13a4). Paired t-test. Data are presented as mean ± SEM (**c**-**e**, **i**-**k**) or as mean with individual data points (**b**, **f**-**h**, **m**-**r**, **t**). Source data are provided as a Source Data file.

Supplementary Fig. 7: Disease-associated ATP13A4 variants.

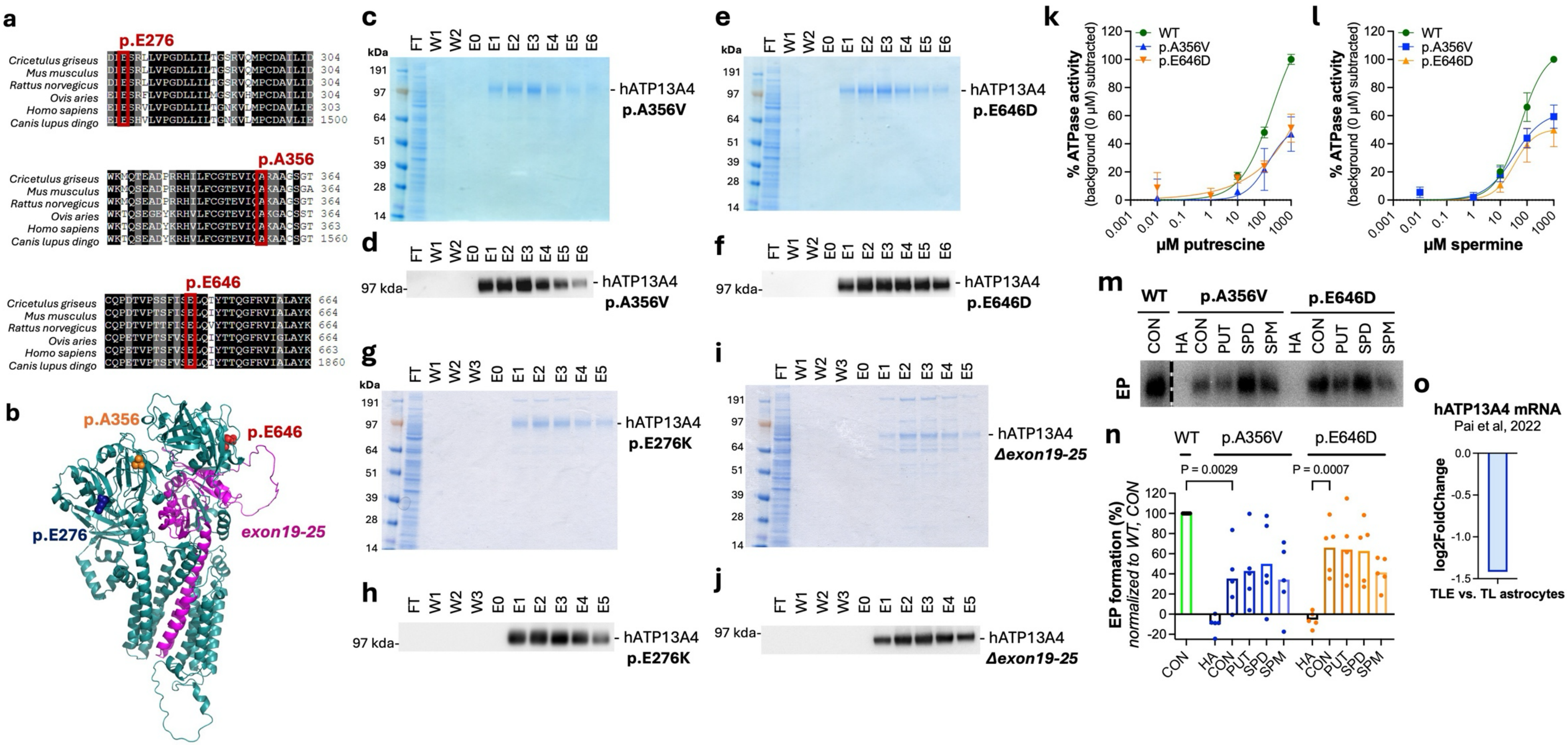

Supplementary Fig. 7: Disease-associated ATP13A4 variants. a, Sequence alignment showing disease-associated residues are highly conserved. The alignment was generated using Clustal Omega (13). **b**, Predicted 3D structure of ATP13A4 (AlphaFold) with disease-associated residues indicated. **c-j**, Coomassie staining (**c**, **e**, **g**, **i**) and Western blot analysis (**d**, **f**, **h**, **j**) showing the purification process for the disease-associated ATP13A4 variants p.A356V (**c**-**d**), p.E646D (**e**-**f**), p.E276K (**g**-**h**) and *Δexon19-25* (**i-j**) starting from detergent-solubilized HEK293T cell lysates, followed by streptavidin affinity chromatography and subsequent elution with biotin. $n \geq 3$ independent purifications. FT, flow-through; W, wash; E, elution. **k**-**l**, Dose-response curve showing the effect of putrescine (**k**) and spermine (**l**) on the ATPase activity of solubilized purified ATP13A4 (WT or disease-associated p.A356V and p.E646D variants). Data are presented as mean ± SEM. $n = 4$ independent experiments. **m**-**n**, Phosphoenzyme (EP) levels of purified, solubilized ATP13A4 (WT and disease-associated variants p.A356V and p.E646D) under control (CON) conditions and in the presence of putrescine (PUT), spermidine (SPD), spermine (SPM), or hydroxylamine (HA). **m**, Representative autoradiogram. **n**, Quantification of EP in **m**. Data are presented as mean with individual data points. $n = 5$ independent experiments. Two-way ANOVA with Tukey's multiple comparisons test. **o**, Human RNA-seq data (from (14)) showing that *ATP13A4* is differentially downregulated in human epilepsy astrocytes (temporal lobe epilepsy (TLE) *versus* temporal lobe (TL) control). Source data are provided as a Source Data file.

Supplementary Fig. 8: Validation of the *Atp13a4* knockout mouse model.

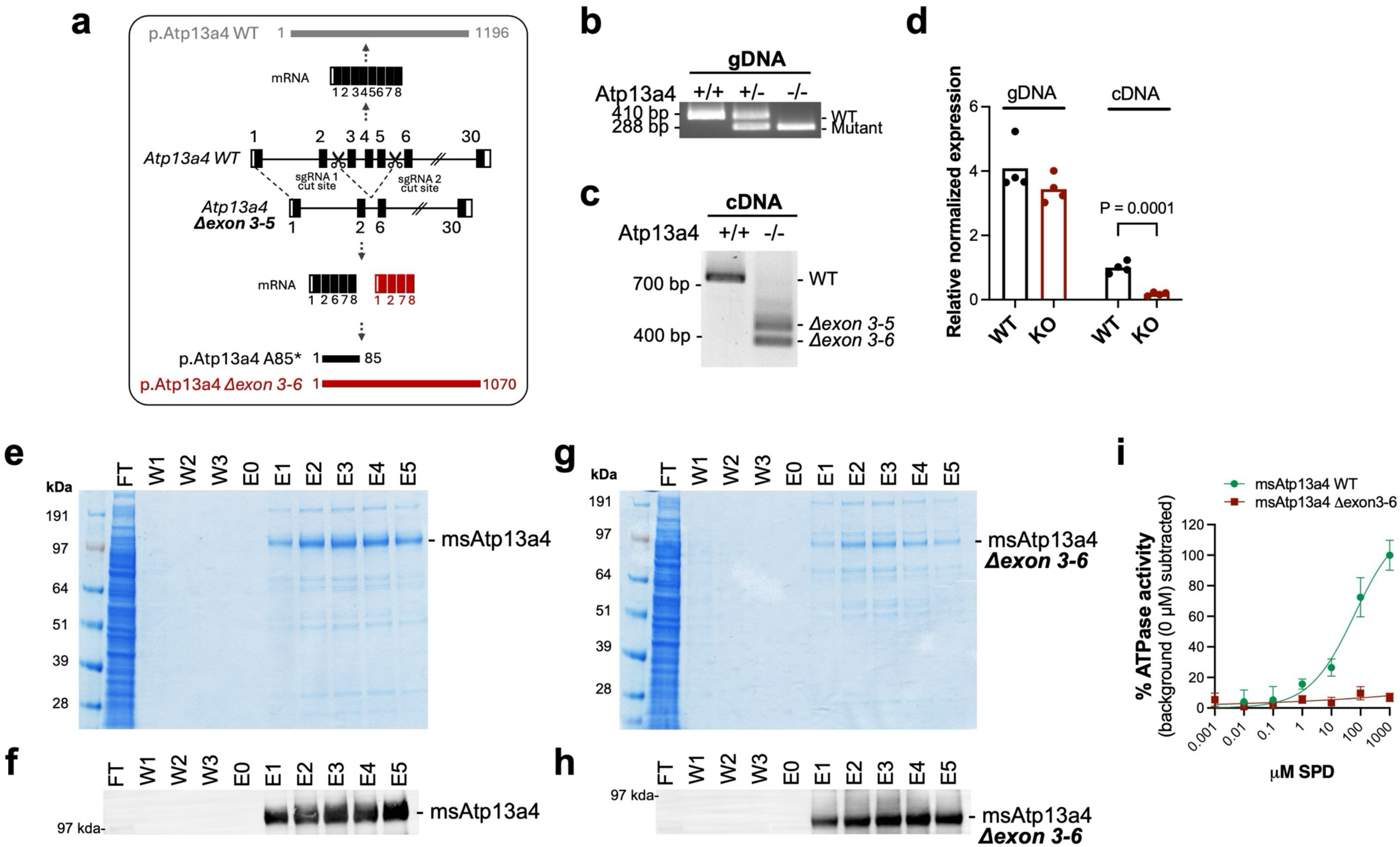

Supplementary Fig. 8: Validation of the *Atp13a4* knockout mouse model. a, Schematic representation of the CRISPR-Cas9-generated *Atp13a4* knockout allele, showing deletion of exons 3-5 and the resulting transcript variants. The deletion introduces a premature stop codon (Ala85*) and is predicted to generate a truncated protein lacking essential functional domains. An alternative splice variant lacking exon 6 (Δexon3-6) is indicated. **b**, Genomic PCR confirming deletion of exons 3-5 in homozygous *Atp13a4* KO mice compared to WT and heterozygous mice. **c**, RT-PCR analysis of cortical RNA showing loss of the full-length *Atp13a4* transcript and the presence of aberrant exon-deleted products in KO mice. Sanger sequencing of excised bands confirmed the expected exon 3-5 deletion and additionally identified a variant transcript lacking exon 6, indicating altered downstream splicing. *n* = 4 WT and 5 KO mice. **d**, qPCR analysis targeting exon 6 demonstrating reduced transcript levels in KO cDNA despite comparable genomic DNA levels, consistent with altered splicing and transcript instability. *n* = 4 mice per genotype. Two-tailed unpaired t-test. **e**-**h**, Purification of recombinant WT and Δexon3-6 msAtp13a4 proteins, visualized by Coomassie staining (**e-f**) and corresponding immunoblot analysis (**g-h**). $n \geq 3$ independent purifications. **i**, ATPase activity assay showing robust spermidine-stimulated activity of WT msAtp13a4, whereas the Δexon3-6 variant displays no detectable stimulation, demonstrating loss of catalytic function. *n* = 3 independent experiments. Data are presented as mean with individual data points (**d**) or mean ± SEM (**i**). Source data are provided as a Source Data file.

Supplementary Fig. 9: Disease-associated C-terminal deletion fails to rescue astrocyte morphological defects in an *Atp13a4* KO background.

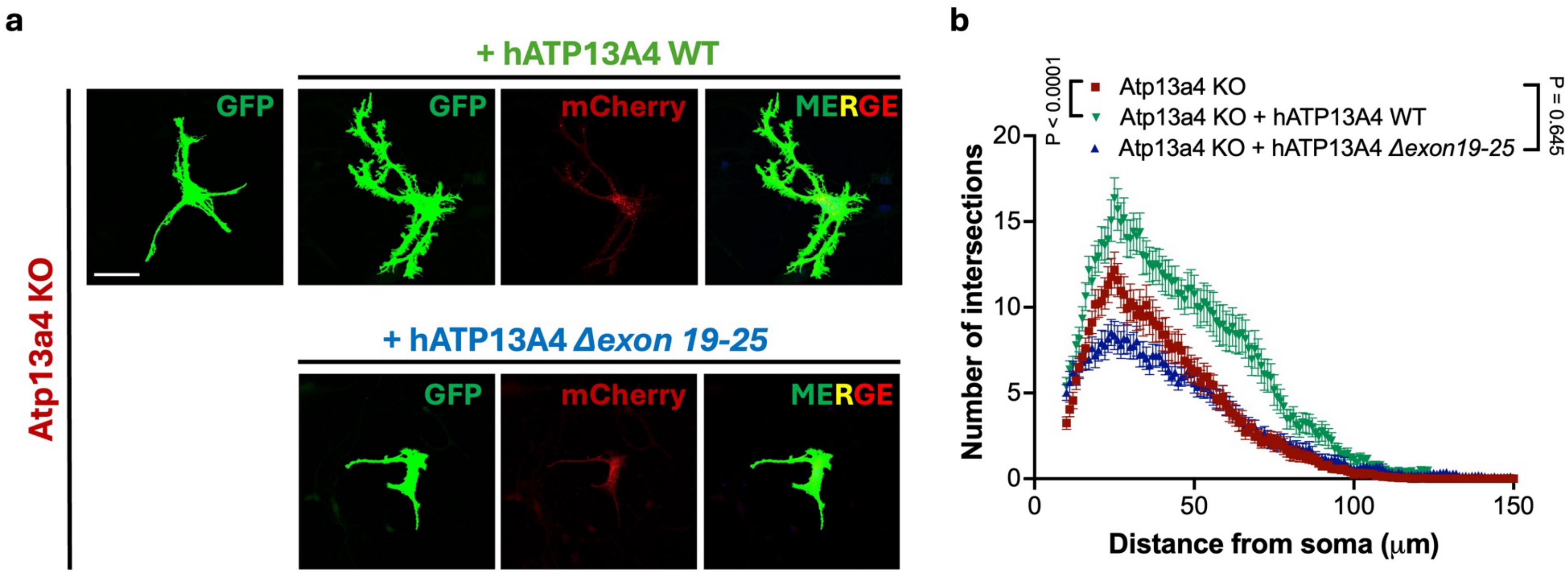

Supplementary Fig. 9: Disease-associated C-terminal deletion fails to rescue astrocyte morphological defects in an *Atp13a4* KO background. a, Representative images of GFP-transfected *Atp13a4* KO astrocytes (green) in the presence or absence of hATP13A4 constructs (WT or Δexon19-25) with an N-terminal mCherry tag (red) after 48 h co-culture with WT neurons. Scale bar, 50 µm. **b**, Quantification of astrocyte complexity for conditions in **a**. *n* = 41 (KO) and 40 (KO + WT, KO + Δexon19-25) astrocytes from two independent experiments. Data are presented as mean ± SEM. Mixed effects ANOVA with Tukey's post-hoc test. Source data are provided as a Source Data file.

Supplementary Fig. 10: Polyamines decrease astrocyte complexity in an ATP13A4-dependent manner.

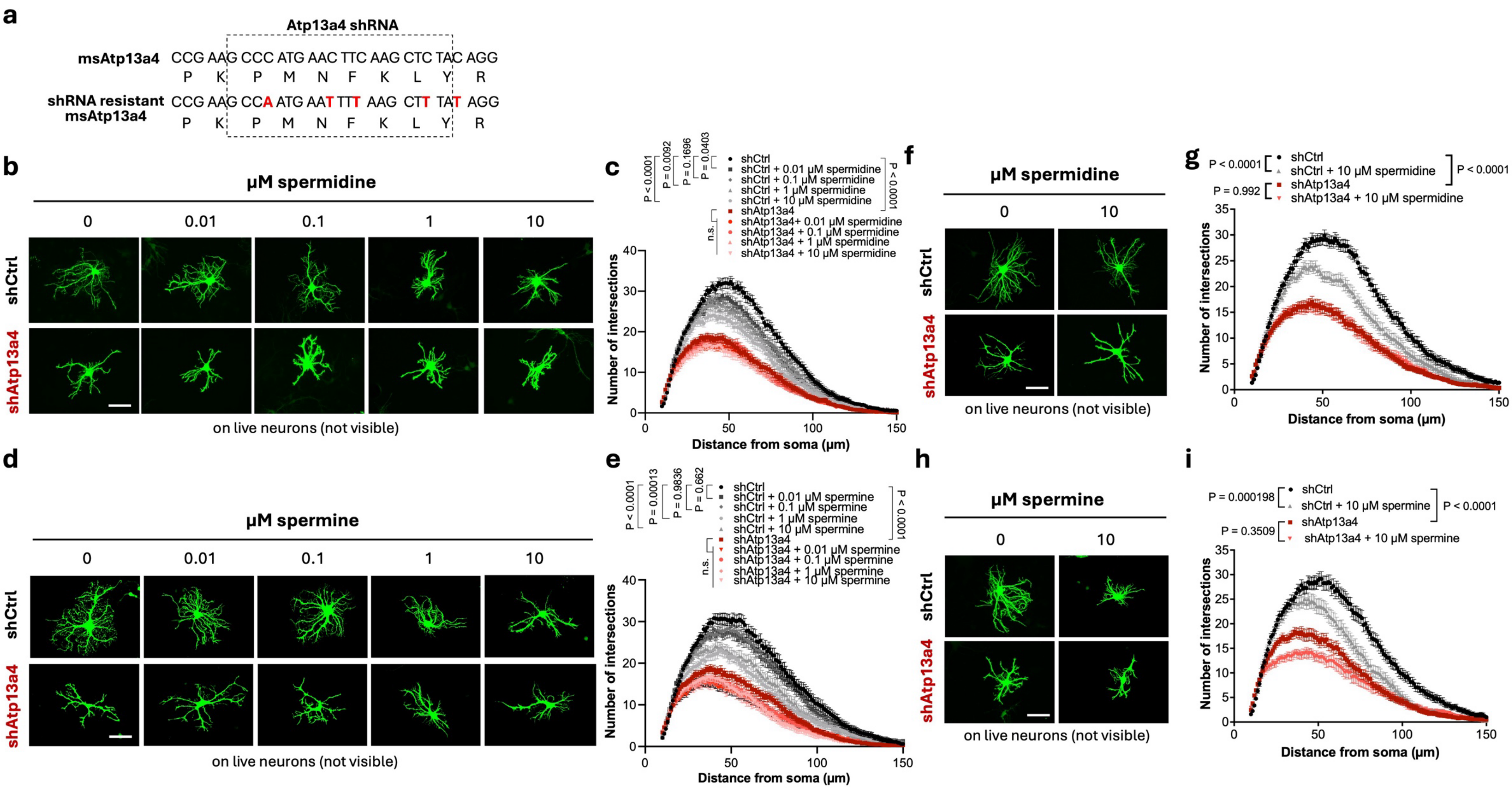

Supplementary Fig. 10: Polyamines decrease astrocyte complexity in an ATP13A4-dependent manner. a, shRNA-resistant ATP13A4 construct with silent mutations (red) in the shRNA target site to prevent knockdown. **b**, Representative images of astrocytes (green) transfected with shRNA targeting mouse/rat Atp13a4 (shAtp13a4) or a scrambled control (shCtrl), co-cultured with WT neurons (not labeled) for 48 h, and treated with spermidine during the final 8 h of co-culture. Scale bar, 50 μm. **c**, Quantification of astrocyte complexity via Sholl analysis for conditions in **b**. *n* = 40-68 astrocytes per condition from 2-4 independent experiments. Exact sample sizes are provided in the Source Data file. **d**, Representative images of shRNA expressing astrocytes co-cultured with WT neurons for 48 h and treated with spermine during the final 8 h of co-culture. Scale bar, 50 μm. **e**, Quantification of astrocyte complexity for conditions in **d**. *n* = 60 astrocytes per condition from 3 independent experiments, except shAtp13a4, 0.01 μM (*n* = 50 astrocytes). **f**, Representative images of shRNA expressing astrocytes (green), treated with spermidine for 24 h and then co-cultured with WT neurons for 48 h. Scale bar, 50 μm. **g**, Quantification of astrocyte complexity for conditions in **f**. *n* = 60-63 astrocytes per condition from three independent experiments. Exact sample sizes are provided in the Source Data file. **h**, Representative images of shRNA expressing astrocytes (green), treated with spermine for 24 h and then co-cultured with WT neurons for 48 h. Scale bar, 50 μm. **i**, Quantification of astrocyte complexity for conditions in **h**. *n* = 60 astrocytes per condition from three independent experiments. All data are presented as mean ± SEM. **c**, **e**, **g**, **i**, Mixed effects ANOVA with Tukey's post-hoc test. Source data are provided as a Source Data file.

Supplementary Fig. 11: Atp13a4 knockdown in astrocytes does not impact neuronal morphology or inhibitory synapse function.

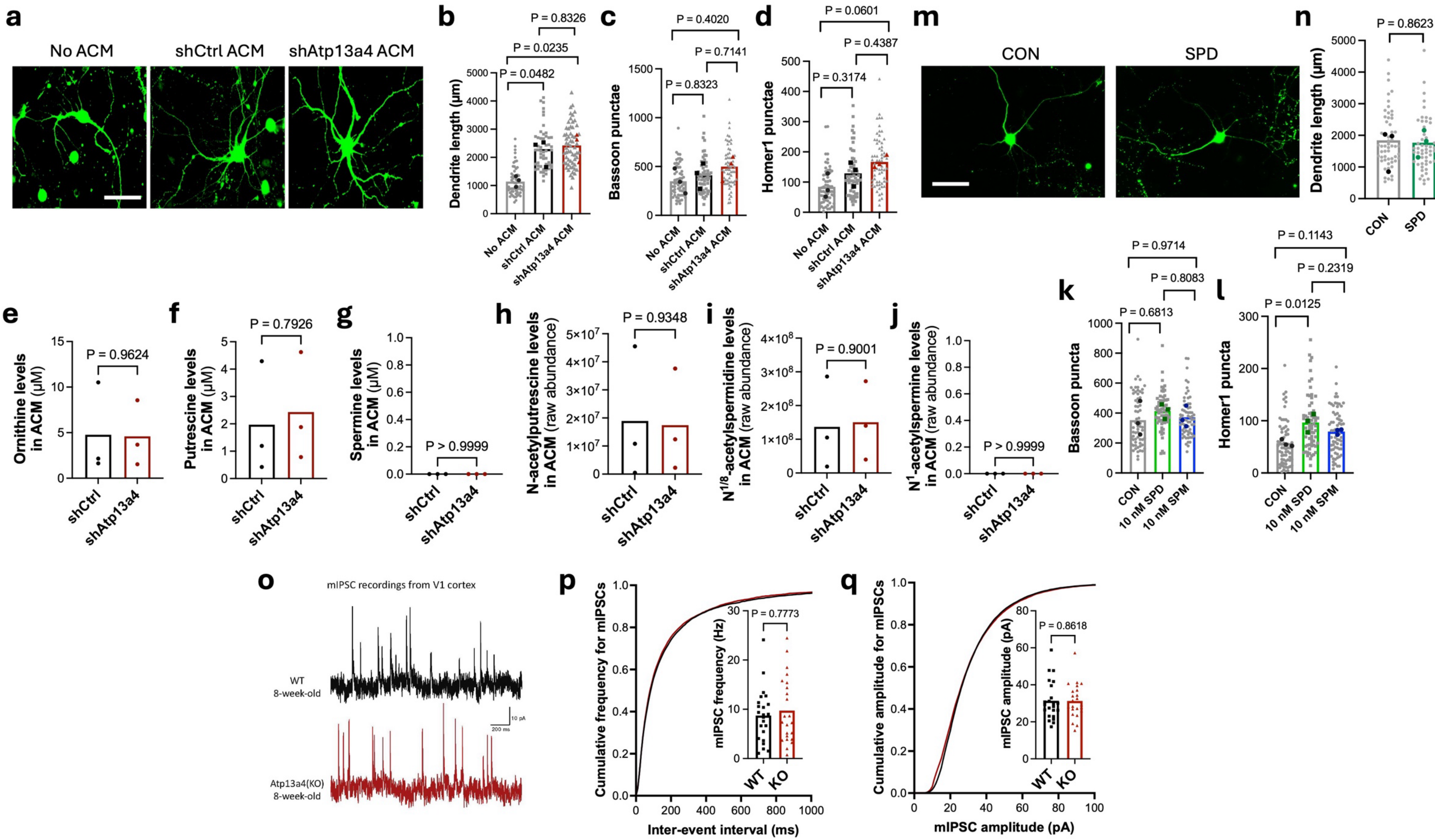

Supplementary Fig. 11: Atp13a4 knockdown in astrocytes does not impact neuronal morphology or inhibitory synapse function. **a**, Representative images of neurons transfected with GFP and treated with astrocyte-conditioned medium (ACM). Scale bar: 100 µm. **b**, Quantification of dendrite tree length in **a**. *n* = 58 (no ACM), 52 (shCtrl ACM), 60 (shAtp13a4 ACM) cells from three independent experiments. **c**-**d**, Quantification of Bassoon (**c**) and Homer1 (**d**) puncta of neuronal cultures treated with ACM (Fig. 4b), *n* = 60 (no ACM), 58 (shCtrl ACM), 64 (shAtp13a4) cells from three independent experiments. **b**-**d**, Individual data points shown (light grey), with experiment averages plotted in grey (no ACM), black (shCtrl ACM), red (shAtp13a4 ACM). Nested one-way ANOVA with Tukey's post-test. **e**-**g**, Metabolomics analysis of ornithine (**e**), putrescine (**f**), spermine (**g**), N-acetylputrescine (**h**), $N^{1/8}$-acetylspermidine (**i**), and N^{1}-acetylspermine (**j**) levels in shCtrl ACM versus shAtp13a4 ACM. *n* = 3 independent experiments. Unpaired two-tailed t-test, except panels **g** and **j** (two-tailed Mann-Whitney test). **k-l**, Quantification of Bassoon (**k**) and Homer1 (**l**) puncta of neuronal cultures treated with SPD or SPM (Fig. 4e). *n* = 64 (CON), 66 (SPD), 65 (SPM) cells from three independent experiments. Individual data points shown (light grey), with experiment averages plotted in grey (no ACM), green (SPD) or blue (SPM). Nested one-way ANOVA with Tukey's post-test. **m**, Representative images of neurons transfected with GFP and treated with vehicle control (CON) or spermidine (10 nM). Scale bar: 100 µm. **n**, Quantification of dendrite tree length in **m**. *n* = 54 (CON), 52 (SPD) cells from three independent experiments. Individual data points shown (light grey), with experiment averages plotted in grey (CON) or green (SPD). Nested two-tailed t-test. **o**, Representative mIPSC (miniature inhibitory postsynaptic currents) traces from V1 cortex in acute brain slices of WT and *Atp13a4* KO mice. **p**-**q**, Quantification of frequency (**p**) and amplitude (**q**) averages (inset) and cumulative probability of mIPSC from *Atp13a4* WT and KO neurons. *n* = 23 neurons from 3 mice per genotype. Two-tailed Mann-Whitney test (inset); Kolmogorov-Smirnov test (cumulative probability). Individual data points shown. Source data are provided as a Source Data file.

Supplementary Fig. 12. Extended metabolomic profiling of plasma, cortex, and CSF in two-month-old *Atp13a4* WT and KO mice.

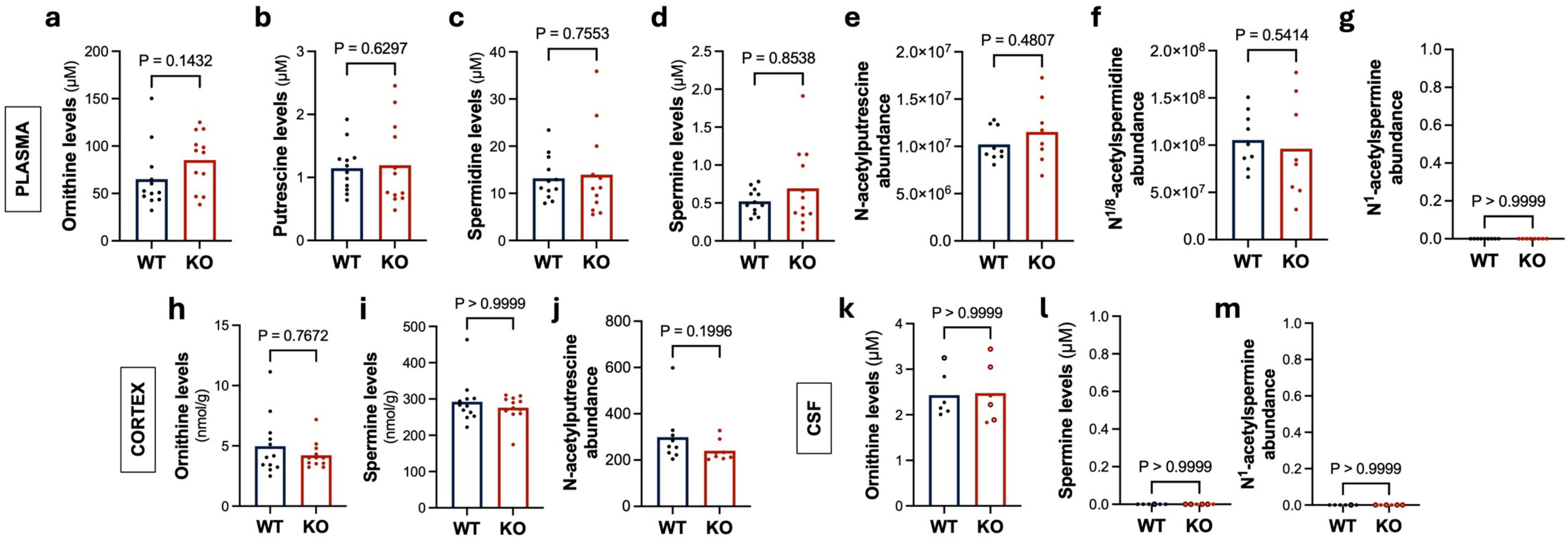

Supplementary Fig. 12. Extended metabolomic profiling of plasma, cortex, and CSF in two-month-old *Atp13a4* WT and KO mice.
Targeted LC-MS/MS quantification of ornithine (**a**), putrescine (**b**), spermidine (**c**), spermine (**d**), N-acetylputrescine (**e**), $N^{1/8}$-acetylspermidine (**f**) and N^1-acetylspermine (**g**) in plasma from *Atp13a4* WT and KO mice. For **a**-**d**, *n* = 12 (WT) and 12 (KO) mice; for **e**-**g**, *n* = 9 (WT) and 8 (KO) mice. **h**-**j**, Quantification of ornithine (**h**), spermine (**i**), and N-acetylputrescine (**j**) in cortical tissue. For **h**-**i**, *n* = 12 (WT) and 12 (KO) mice; for **j**, *n* = 9 (WT) and 8 (KO) mice. **k**-**m**, Quantification of ornithine (**k**), spermine (**l**), and N^1-acetylspermine (**m**) in cerebrospinal fluid (CSF). *n* = 6 biological samples per genotype. Filled symbols indicate single-animal samples; open symbols indicate pooled samples derived from 2-3 mice. Data are presented as mean, with individual data points representing individual mice (**a**-**j**) or biological samples (**k**-**m**). Statistical comparisons were performed using two-tailed Mann-Whitney tests. Source data are provided as a Source Data file.

Supplementary Fig. 13: ATP13A4 loss induces selective adaptations in the polyamine pathway without evidence of microglial activation.

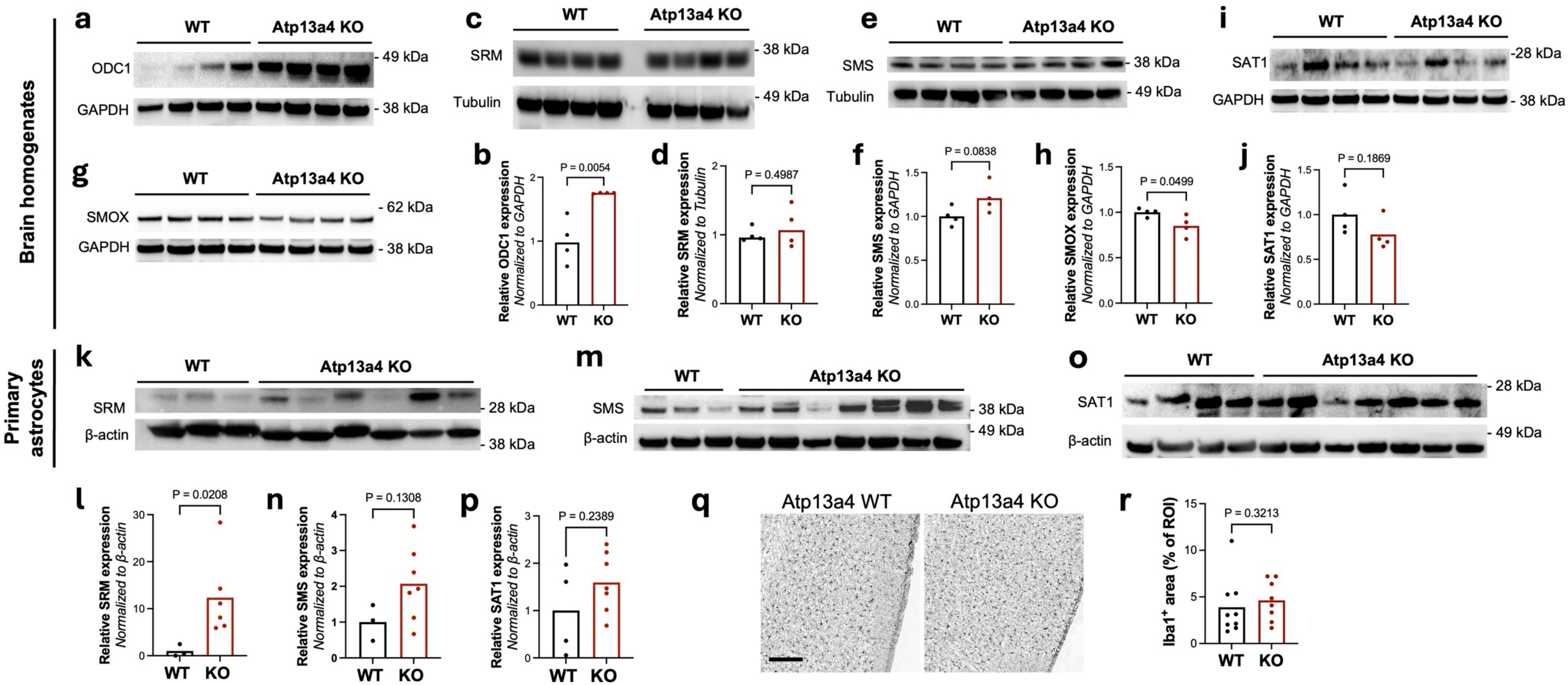

Supplementary Fig. 13: ATP13A4 loss induces selective adaptations in the polyamine pathway without evidence of microglial activation. a-**j**, Western blot analyses and corresponding quantifications of ODC1 (**a**-**b**), SRM (**c**-**d**), SMS (**e**-**f**), SMOX (**g**-**h**) and SAT1 (**i**-**j**) expression in brain homogenates from *Atp13a4* WT (*n* = 4) and KO (*n* = 4) mice. GAPDH and tubulin served as loading controls. **k**-**p**, Western blot analyses and corresponding quantifications of SRM (**k**-**l**; *n* = 3 WT and 6 KO), SMS (**m**-**n**; *n* = 3 WT and 7 KO) and SAT1 (**o**-**p**; *n* = 4 WT and 7 KO) expression in primary astrocytes from *Atp13a4* WT and KO mice. β-actin served as a loading control. Data are presented as mean, with individual data points representing independent biological replicates (**a**-**j**, individual mice; **k**-**p**, individual primary astrocyte preparations). Statistical comparisons were performed using unpaired two-tailed t-tests, except for **l** (Welch's t-test). **q**, Iba1 immunostaining in cortex of two-month-old *Atp13a4* WT and KO mice. Scale bar, 50 µm. **r**, Quantification of cortical Iba1 immunoreactivity. Data points represent mouse averages. *n* = 8 (KO) and 9 (WT) mice. Statistical comparisons were performed using a two-tailed Mann-Whitney test. Source data are provided as a Source Data file.

Supplementary Fig. 14: Early neurodevelopmental impairments in *Atp13a4* KO mice occur similarly in male and female pups.

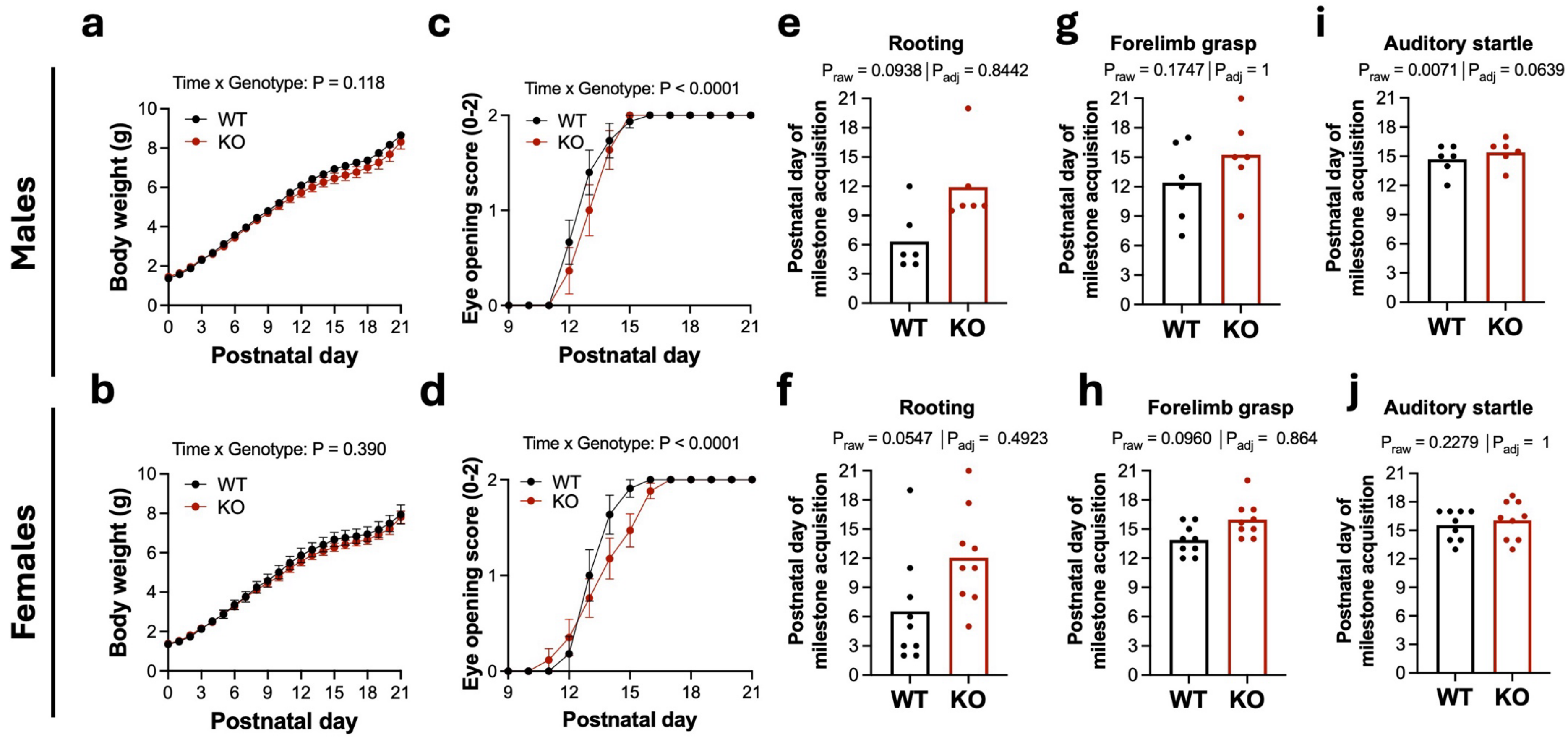

Supplementary Fig. 14: Early neurodevelopmental impairments in *Atp13a4* KO mice occur similarly in male and female pups. Developmental milestone acquisition (from Fig. 6a, b, f, h, j) was analyzed separately in male (**a**, **c**, **e**, **g**, **i**) and female (**b**, **d**, **f**, **h**, **j**) *Atp13a4* WT and KO littermates. **a**-**b**, Body weight progression over time. **c**-**d**, Eye opening acquisition. For **a**, n = 11 (WT, female), 15 (WT, male), 19 (KO, female) and 12 (KO, male) mice; for **b**, n = 11 (WT, female), 15 (WT, male), 17 (KO, female) and 11 (KO, male) mice; animals with unilateral eye development were excluded from this analysis. Statistical comparisons were performed using a linear mixed-effects model (**a**) and a cumulative link mixed model (**b**). Data are presented as mean ± SEM (**a**-**d**). **e**-**f**, Rooting. **g**-**h**, Forelimb grasp. **i**-**j**, Auditory startle. Data are presented as mean with individual data points representing litter-level genotype averages from matched litters. n = 6 (male), 9 (female) litters. For milestone analyses, acquisition is defined as the second consecutive day on which pups met the predefined criterion. Statistical comparisons were performed using paired two-tailed t-tests (**g**-**j**) or Wilcoxon matched-pairs signed-rank tests (**e**-**f**), as appropriate based on normality. P values were adjusted for multiple comparisons across the nine milestone tests using Bonferroni correction (adjusted threshold α = 0.0056; or equivalently, raw p-values multiplied by 9 and compared to 0.05). Source data are provided as a Source Data file.

Supplementary Fig. 15: Behavioral characterization of two-month-old *Atp13a4* KO mice reveals mild, female-biased differences.

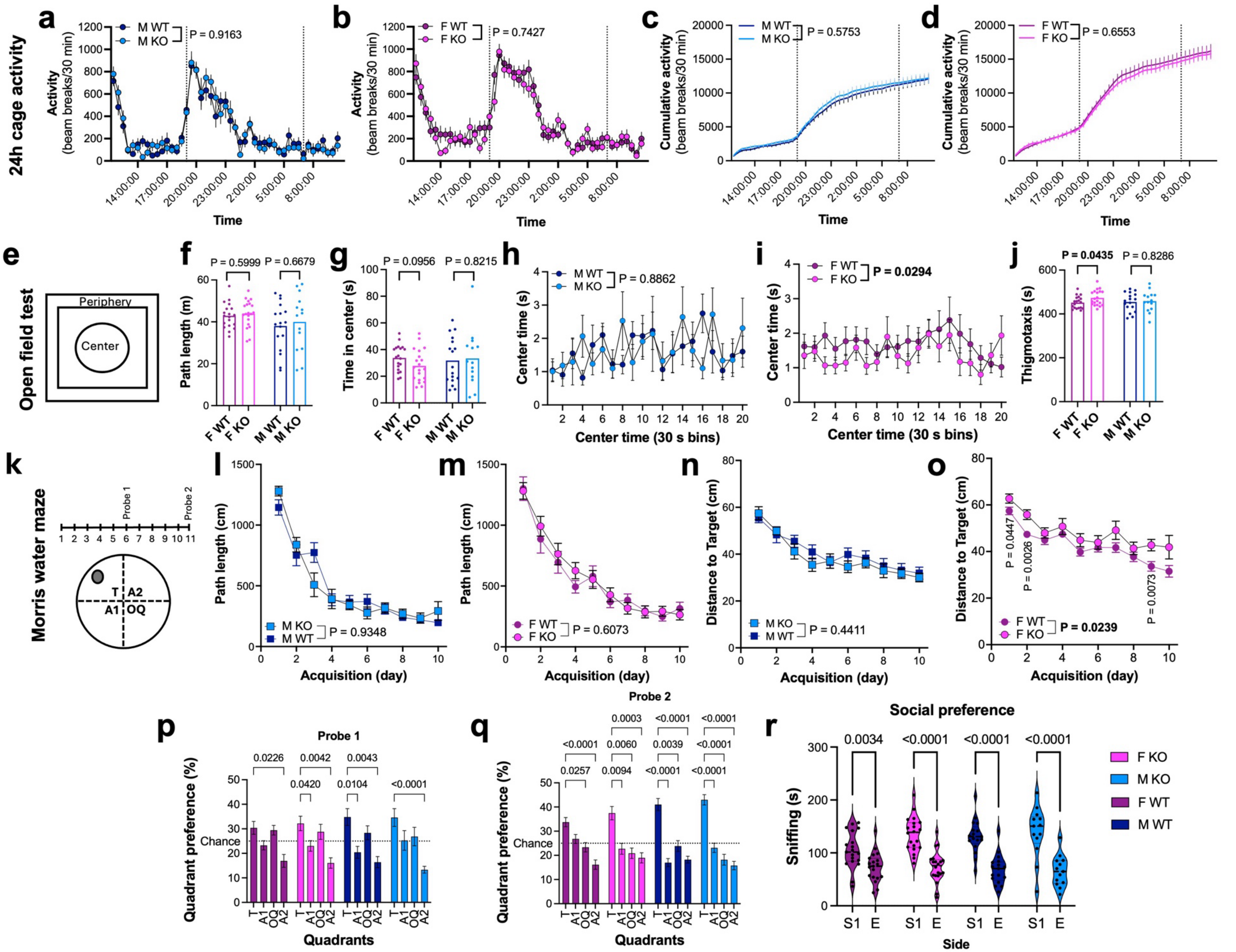

Supplementary Fig. 15: Behavioral characterization of two-month-old *Atp13a4* KO mice reveals mild, female-biased differences. **a**-**d**, 24 h home-cage activity monitoring in male (**a**, **c**) and female (**b**, **d**) mice. Beam breaks (30 min bins), plotted as time-resolved (**a**-**b**) or cumulative (**c**-**d**) activity. Dotted lines, lights off (19:00)/on (07:00). No genotype-dependent differences in locomotor activity or circadian patterns. *n* = 14 (KO/male), 12 (WT/male), 19 (KO/female), 18 (WT/female). **e**-**j**, Open field test. **e**, Arena schematic. **f**, Total distance traveled (path length), showing no genotype differences. **g**, Time spent in center. **h-i**, Center time in 30 s bins for male (**h**) and female (**i**) mice. Global nonlinear regression compared single versus separate curve fits. Female KO mice show altered center exploration, males unaffected. **j**, Time in periphery (thigmotaxis), increased in female KO mice. *n* = 14 (KO/male), 17 (WT/male), 20 (KO/female), 19 (WT/female). **k**-**q**, Morris water maze. **k**, Schematic showing 10 training days and probe trials on day 6/11. T, target quadrant; A1/A2, adjacent quadrants; OQ, opposite quadrant. **l**-**m,** Total distance traveled to platform during acquisition in male (**l**) and female (**m**) mice. **n-o,** Distance to platform across training days in male (**n**) and female (**o**) mice. Female KO mice show increased distance, indicating a mild learning delay; males are unaffected. **p**-**q,** Probe trials on days 6 (**p**) and 11 (**q**). Dotted line, chance (25 %). All groups show comparable target quadrant preference, indicating intact memory retention. **l**-**q**, n = 14 (KO/male), 17 (WT/male), 20 (KO/female), and 19 (WT/female). For probe trials (**p**-**q**), three female KO mice were excluded due to persistent floating. **r**, Three-chamber social preference test. Violin plots show time spent in sniffing zones (Stranger mouse 1, S1; empty, E) during the first 5 min. Normal social preference across groups. *n* = 13 (KO/male), 17 (WT/male), 20 (KO/female), 19 (WT/female). Data are mean ± SEM, except **f**, **g**, **j** and **r** (individual data points). Statistics: unpaired two-tailed t-test (**f**, **g**, **j**), mixed-effects analysis (REML) with Tukey's (**a**-**d**) or Dunnett's (**l**-**o**) post-tests, two-way RM ANOVA with Dunnett's post-test (**p**, **q**), two-way ANOVA with Tukey's post-test (**r**). Source data are provided as a Source Data file.

Supplementary Fig. 16: Female-biased alterations in PTZ-induced immobility dynamics in two-month-old *Atp13a4* KO mice.

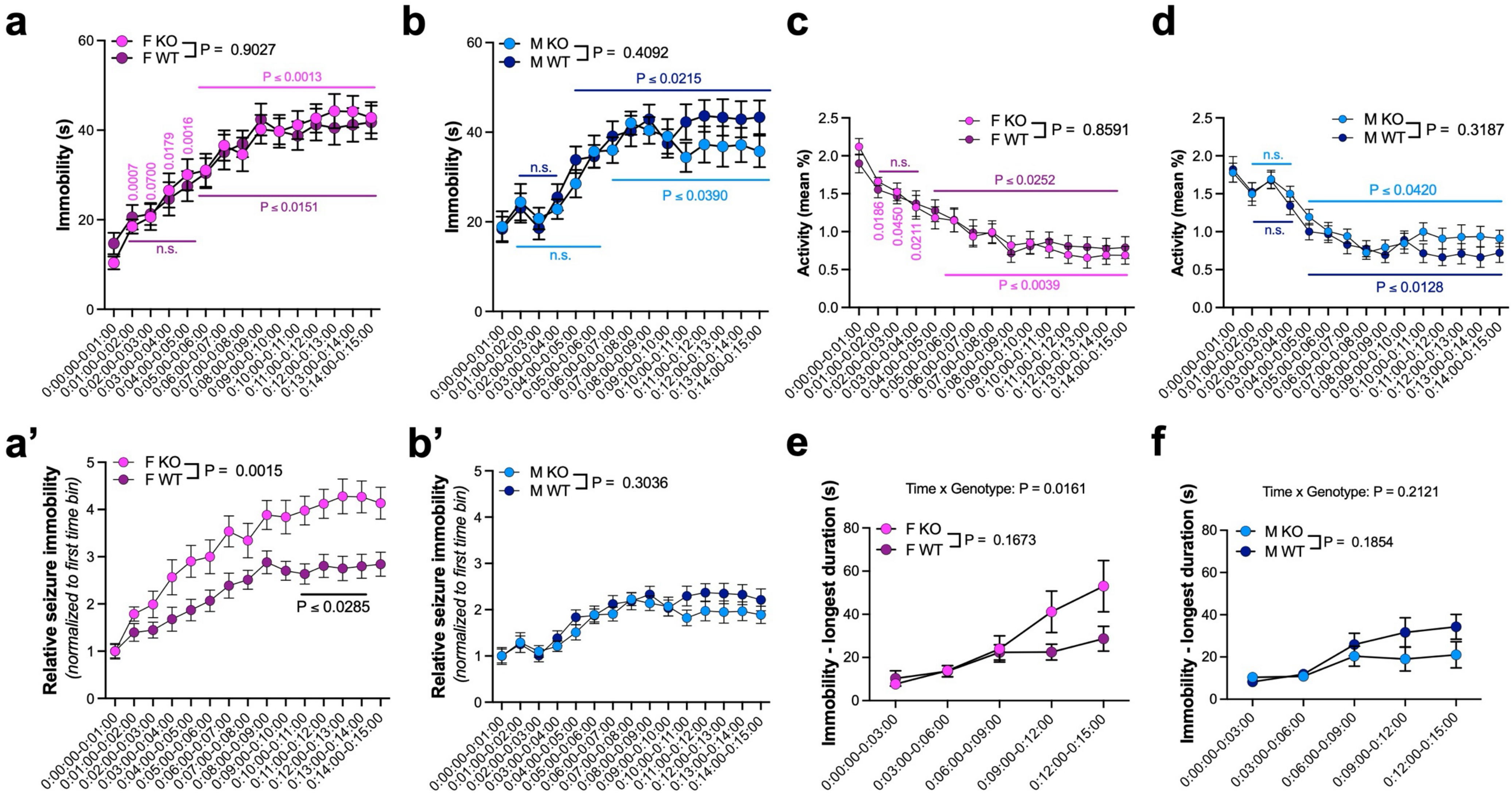

Supplementary Fig. 16: Female-biased alterations in PTZ-induced immobility dynamics in two-month-old *Atp13a4* KO mice. a-**b**, Immobility time following PTZ (pentylenetetrazol) challenge. Immobility duration is analyzed in 60 s time bins in female (**a**) and male (**b**) mice. Female *Atp13a4* KO mice exhibit an earlier increase in immobility compared to WT, whereas male mice show no genotype-dependent differences. **a'**-**b'**, Normalized immobility (same dataset as **a**-**b**). Immobility is normalized to each mouse's baseline (*i.e.* first time bin) and plotted over time in female (**a'**) and male (**b'**) mice. This normalization highlights differences in the temporal onset of immobility independent of baseline activity levels. Female KO mice show an earlier increase compared to WT, whereas males are unaffected. **c**-**d**, Locomotor activity following PTZ injection. Activity is expressed as mean % over time in female (**c**) and male (**d**) mice. While overall activity levels are comparable between genotypes, female *Atp13a4* KO mice exhibit an earlier decline in activity following PTZ injection, consistent with an earlier transition into immobility. Male mice show no genotype-dependent differences. **e**-**f**, Longest immobility episode. Duration of the longest immobility bout is analyzed in 3 min time bins in female (**e**) and male (**f**) mice. A significant genotype × time interaction is observed in females but not in males, indicating altered temporal progression of immobility. Data are presented as mean ± SEM. n = 20 (KO, female), 19 (WT, female), 14 (KO, male), 17 (WT, male). Statistical comparisons were made using mixed-effects analysis (REML) with Dunnett's (**a**-**b**) or Šidák's (**c-d**) multiple comparisons test, two-way RM ANOVA with Šidák's post-test (**a'-b', e-f**). Source data are provided as a Source Data file.

Supplementary Fig. 17: Polyamine homeostasis and astrocyte reactivity are similarly altered across sexes, while excitatory synapse density shows sex-dependent differences in two-month-old *Atp13a4* KO mice.

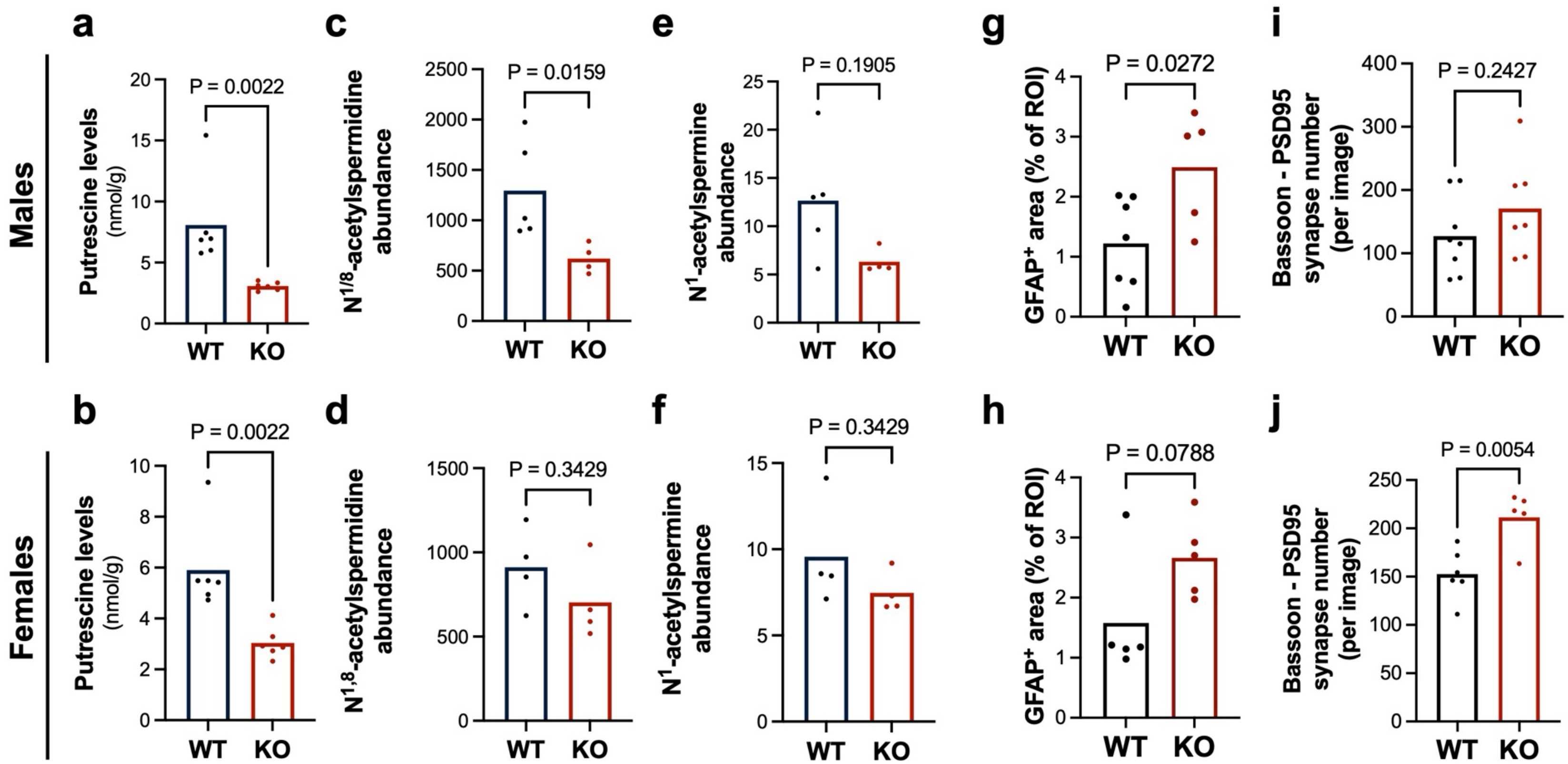

Supplementary Fig. 17: Polyamine homeostasis and astrocyte reactivity are similarly altered across sexes, while excitatory synapse density shows sex-dependent differences in two-month-old *Atp13a4* KO mice. a-f, Targeted LC-MS/MS quantification of polyamine species in cortical tissue (from Fig. 5a-d) was analyzed separately in male (**a**, **c**, **e**) and female (**b**, **d**, **f**) *Atp13a4* WT and KO mice. Data are presented as mean, with individual data points representing biological replicates (individual mice). Polyamine levels are comparably altered across sexes. **g-h**, Quantification of astrocyte reactivity based on GFAP immunostaining (from Fig. 5m-n) was analyzed separately in male (**g**) and female (**h**) mice. GFAP immunoreactivity is increased in *Atp13a4* KO mice relative to WT controls in both sexes. **i-j**, Quantification of excitatory synapse numbers (Bassoon and PSD95 co-localization) in V1 cortex (from Fig. 5o-p) was analyzed separately in male (**i**) and female (**j**) *Atp13a4* WT and KO mice. Data points represent mouse averages (2 sections/mouse, 3-4 images/section). Statistical comparisons were performed using two-tailed Mann-Whitney test (**a-f**) and unpaired two-tailed t-test (**g-j**). Source data are provided as a Source Data file.

Supplementary Tables

Supplementary Table 1

Sample information and experimental conditions for TurboID study.

**Project**		**6099**						**Samples**											
**Client**																			
**Samples**	Proteomics ID	uL of sample	50 mM Ambic	total uL	starting SDS	casein spike volume	uL of 20% SDS, 50mM TEAB	uL of 200 mM DTT	uL of 400 mM IAA	uL 12% phosphoric acid for final 1.2%	S-trap binding buffer, 90% MeOH, 100mM TEAB pH 7.1	Volume	4x Wash, Binding buffer	20ng/uL trypsin in 50mM TEAB	Elute 50mM TEAB	Elute, 0.2% formic acid	Elute, 50% ACN, 0.2% formic acid	Final Recon uL of 1% TFA 2% ACN	total pmol/casein
**Cyto_A**	75043	60	0	60	0	2	10.58823529	0	3.820433437	7.640866873	588.3467492	672.3962848	150	25	40	40	35	12	1
**Cyto_B**	75044	60	0	60	0	2	10.58823529	0	3.820433437	7.640866873	588.3467492	672.3962848	150	25	40	40	35	12	2
**Cyto_C**	75045	60	0	60	0	2	10.58823529	0	3.820433437	7.640866873	588.3467492	672.3962848	150	25	40	40	35	12	1
**Lamp1_A**	75046	60	0	60	0	2	10.58823529	0	3.820433437	7.640866873	588.3467492	672.3962848	150	25	40	40	35	12	2
**Lamp1_B**	75047	60	0	60	0	2	10.58823529	0	3.820433437	7.640866873	588.3467492	672.3962848	150	25	40	40	35	12	1
**Lamp1_C**	75048	60	0	60	0	2	10.58823529	0	3.820433437	7.640866873	588.3467492	672.3962848	150	25	40	40	35	12	2
**SPQC**	75049																		
													Spin 4000g	1hr, 47C	Spin 4000g	Spin 4000g	Spin 4000g		
	start micro s-trap protocol																		
	freeze lyophilize overnight																		

Supplementary Table 2

Oligonucleotide list.

GENE	SEQUENCE	ADDITIONAL INFORMATION
msAtp13a4	(F): 5’-CCAGCATGCTTTACTCAATG-3’<br>(R): 5’-GAAGATGGATCCAATGAGAC-3’	Primers used for RT-qPCR analysis of C8-D1A cells
msGapdh	(F): 5’-TGTGTCCGTCGTGGATCTGA-3’<br>(R): 5’-CCTGCTTCACCACCTTCTTGA-3’	Primers used for RT-qPCR analysis of C8-D1A cells
shAtp13a4	5’-GCCCATGAACTTCAAGCTCTA-3’	shRNA sequence targeting *msAtp13a4* and *rAtp13a4* for knockdown
shCtrl	5’-GCTCACCACGTGCAATACTAT-3’	scrambled shRNA sequence used as a negative control
miRFLUC	5' -GCGCTGAGTACTTCGAAATGTC- 3'	shRNA sequence targeting FLUC (firefly luciferase) used as a negative control in C8-D1A cells
miR2	5’-GCCAATATTTCAGCAGCTTATT-3’	shRNA sequence targeting *msAtp13a4* for knockdown in C8-D1A cells
miR3	5’-AGCCCATGAACTTCAAGCTCTA-3’	shRNA sequence targeting *msAtp13a4* for knockdown in C8-D1A cells
miR4	5’-GCGATACCTAAACAGTTATCAA-3’	shRNA sequence targeting *msAtp13a4* for knockdown in C8-D1A cells
msAtp13a4	(F): 5’-GGCAGCCCACCTATACAAACTATATAT-3’<br>(R): 5’-GAATGAAAAGACATACGCCCATCT-3’	Primers used for RT-qPCR analysis of primary astrocytes
18S	(F) 5′-GCAATTATTCCCCATGAACG-3′<br>(R) 5′-GGCCTCACTAAACCATCCAA-3′	Primers used for RT-qPCR analysis of primary astrocytes
U6	(F): 5′-GGACTAGTCAGGCCCGAAGGAATAGAAG-3′<br>(R): 5′-GGACTAGTGCCAAAGTGGATCTCTGCTG-3′	Used for amplifying hU6 promoter and shRNA from pLKO.1-shRNA-EGFP
Atp13a4 KO/WT Fwd (*Atp(50715) CF*)	5′-GGTCCCAGAATTCCTTGGCA-3′	Genotyping
Atp13a4 WT Rv (*Atp(50715) WTR*)	5′-CAGTGCTGACAGGGAGATCC-3′	Genotyping
Atp13a4 KO Rv (*Atp(50715) KOR*)	5′-GCCTCACACTGCACTCTTCT-3′	Genotyping
msAtp13a4 (EXON1-7)	(F): 5′-ACCTTGAGAAGAGCCAGCAT-3′ (R): 5′-GAGGTCATACACGGTCAAAGC-3′	Primers used for RT-PCR analysis of cortical RNA
msAtp13a4 (EXON6)	(F): 5′-GAGGTTAATATGTGGGCCTA-3′ (R): 5′-ACCTCCTTGATGAGCAGTTTC-3′	Primers used for RT-qPCR analysis of cortical RNA (exon 6 validation)
msGapdh	(F): 5′-TGTGTCCGTCGTGGATCTGA-3′ (R): 5′-CCTGCTTCACCACCTTCTTGA-3′	Normalization reference for RT-qPCR analysis of cortical RNA (exon 6 validation)

Supplementary References

1. Zhang Y, Sloan SA, Clarke LE, Caneda C, Plaza CA, Blumenthal PD, et al. Purification and Characterization of Progenitor and Mature Human Astrocytes Reveals Transcriptional and Functional Differences with Mouse. Neuron. 2016;89(1):37-53.
2. Velmeshev D, Perez Y, Yan Z, Valencia JE, Castaneda-Castellanos DR, Wang L, et al. Single-cell analysis of prenatal and postnatal human cortical development. Science. 2023;382(6667):eadf0834.
3. Jorstad NL, Close J, Johansen N, Yanny AM, Barkan ER, Travaglini KJ, et al. Transcriptomic cytoarchitecture reveals principles of human neocortex organization. Science. 2023;382(6667):eadf6812.
4. Bakken TE, van Velthoven CT, Menon V, Hodge RD, Yao Z, Nguyen TN, et al. Single-cell and single-nucleus RNA-seq uncovers shared and distinct axes of variation in dorsal LGN neurons in mice, non-human primates, and humans. Elife. 2021;10.
5. Siletti K, Hodge R, Mossi Albiach A, Lee KW, Ding SL, Hu L, et al. Transcriptomic diversity of cell types across the adult human brain. Science. 2023;382(6667):eadd7046.
6. Kang HJ, Kawasawa YI, Cheng F, Zhu Y, Xu X, Li M, et al. Spatio-temporal transcriptome of the human brain. Nature. 2011;478(7370):483-9.
7. Zhang Y, Chen K, Sloan SA, Bennett ML, Scholze AR, O'Keeffe S, et al. An RNA-sequencing transcriptome and splicing database of glia, neurons, and vascular cells of the cerebral cortex. J Neurosci. 2014;34(36):11929-47.
8. Zeisel A, Hochgerner H, Lonnerberg P, Johnsson A, Memic F, van der Zwan J, et al. Molecular Architecture of the Mouse Nervous System. Cell. 2018;174(4):999-1014 e22.
9. Saunders A, Macosko EZ, Wysoker A, Goldman M, Krienen FM, de Rivera H, et al. Molecular Diversity and Specializations among the Cells of the Adult Mouse Brain. Cell. 2018;174(4):1015-30 e16.
10. Tabula Muris C, Overall c, Logistical c, Organ c, processing, Library p, et al. Single-cell transcriptomics of 20 mouse organs creates a Tabula Muris. Nature. 2018;562(7727):367-72.
11. Srinivasan R, Lu TY, Chai H, Xu J, Huang BS, Golshani P, et al. New Transgenic Mouse Lines for Selectively Targeting Astrocytes and Studying Calcium Signals in Astrocyte Processes In Situ and In Vivo. Neuron. 2016;92(6):1181-95.
12. Endo F, Kasai A, Soto JS, Yu X, Qu Z, Hashimoto H, et al. Molecular basis of astrocyte diversity and morphology across the CNS in health and disease. Science. 2022;378(6619):eadc9020.
13. Madeira F, Madhusoodanan N, Lee J, Eusebi A, Niewielska A, Tivey ARN, et al. The EMBL-EBI Job Dispatcher sequence analysis tools framework in 2024. Nucleic Acids Res. 2024;52(W1):W521-W5.
14. Pai B, Tome-Garcia J, Cheng WS, Nudelman G, Beaumont KG, Ghatan S, et al. High-resolution transcriptomics informs glial pathology in human temporal lobe epilepsy. Acta Neuropathol Commun. 2022;10(1):149.

Uncropped blots and autoradiograms for Supplementary Figures

Supplementary Fig. 5: hATP13A4 spontaneously forms a phospho-enzyme that is sensitive to polyamines.

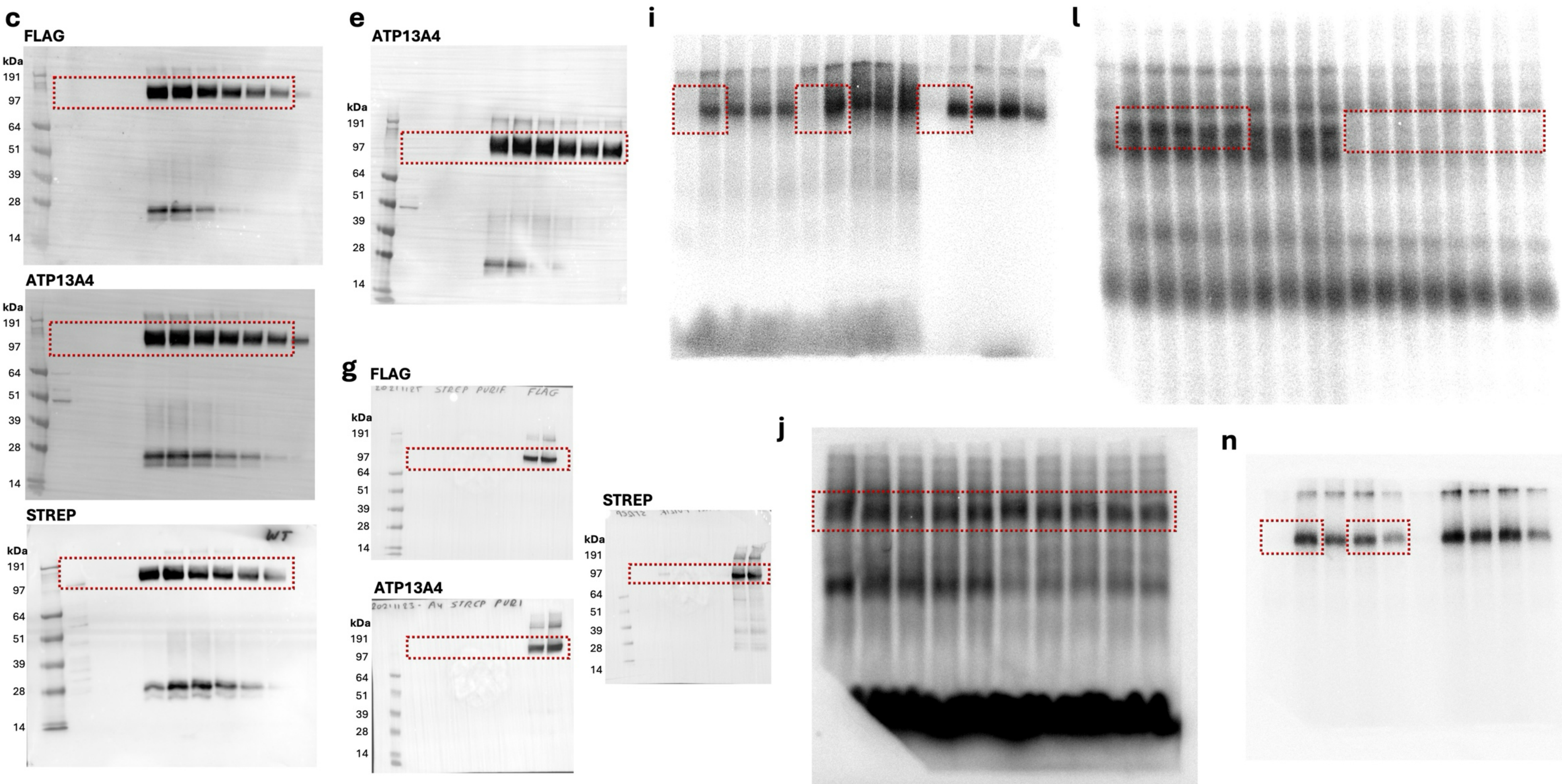

Supplementary Fig. 6: ATP13A4 promotes cellular polyamine uptake.

a

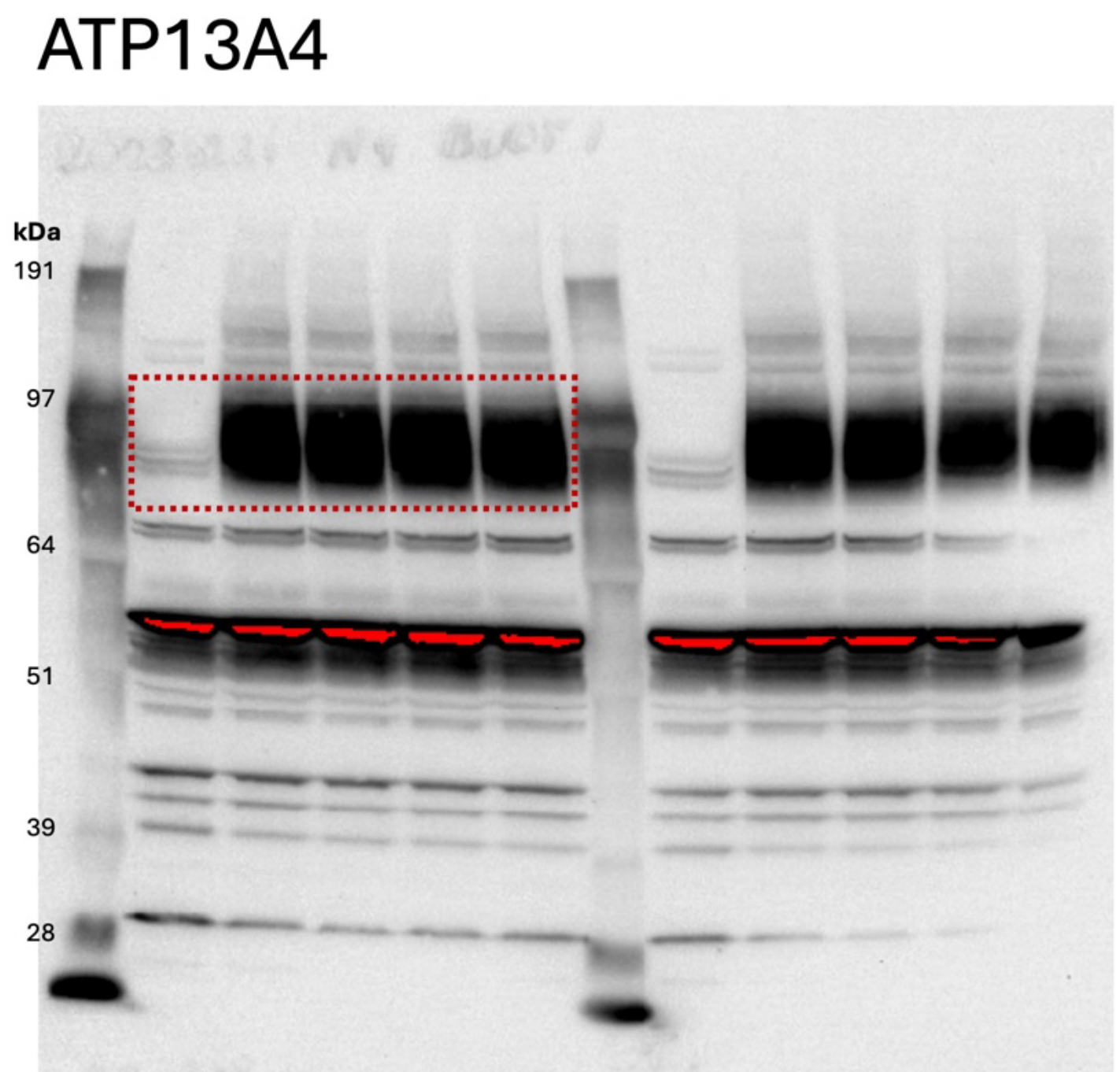

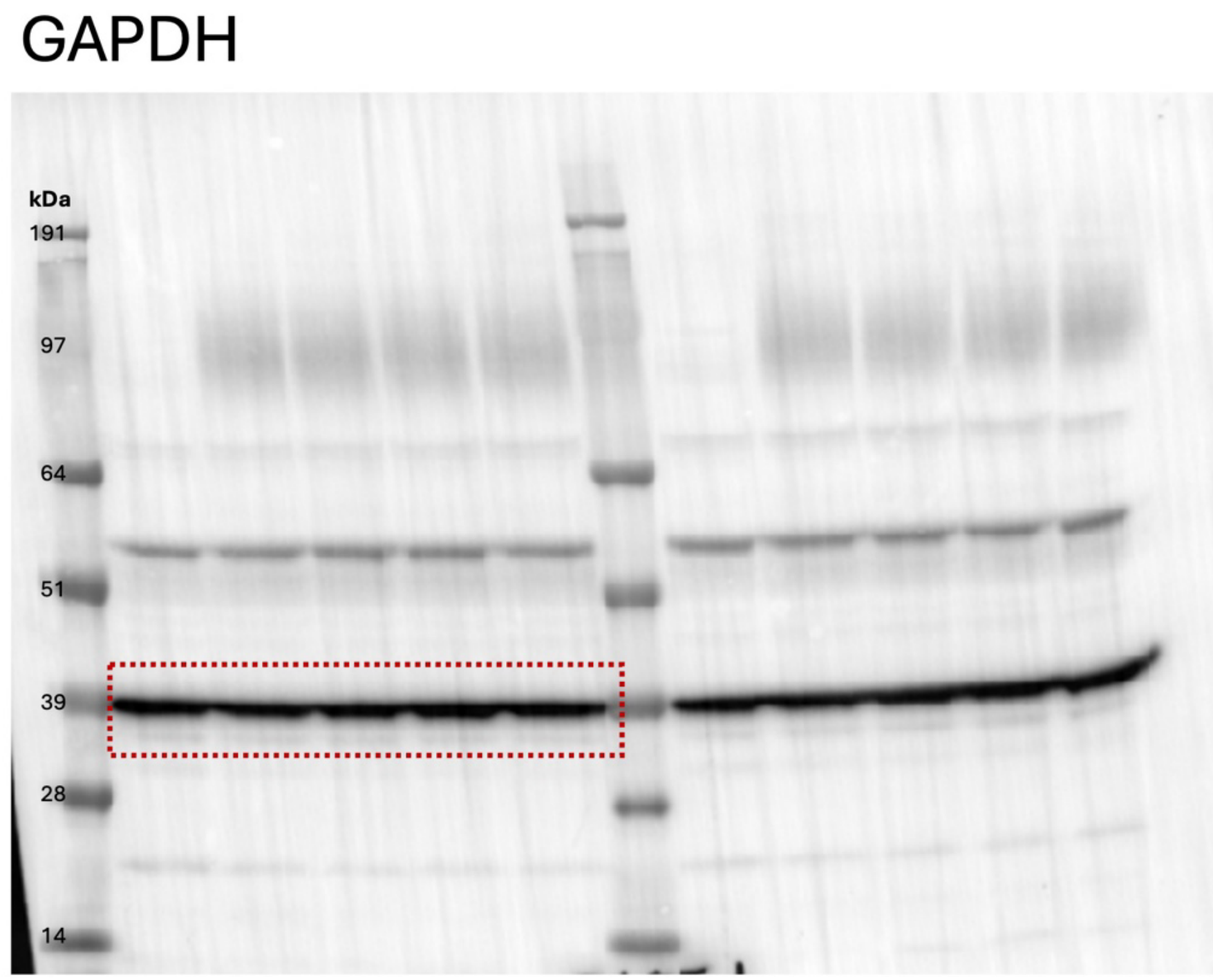

Supplementary Fig. 7: Disease-associated ATP13A4 variants.

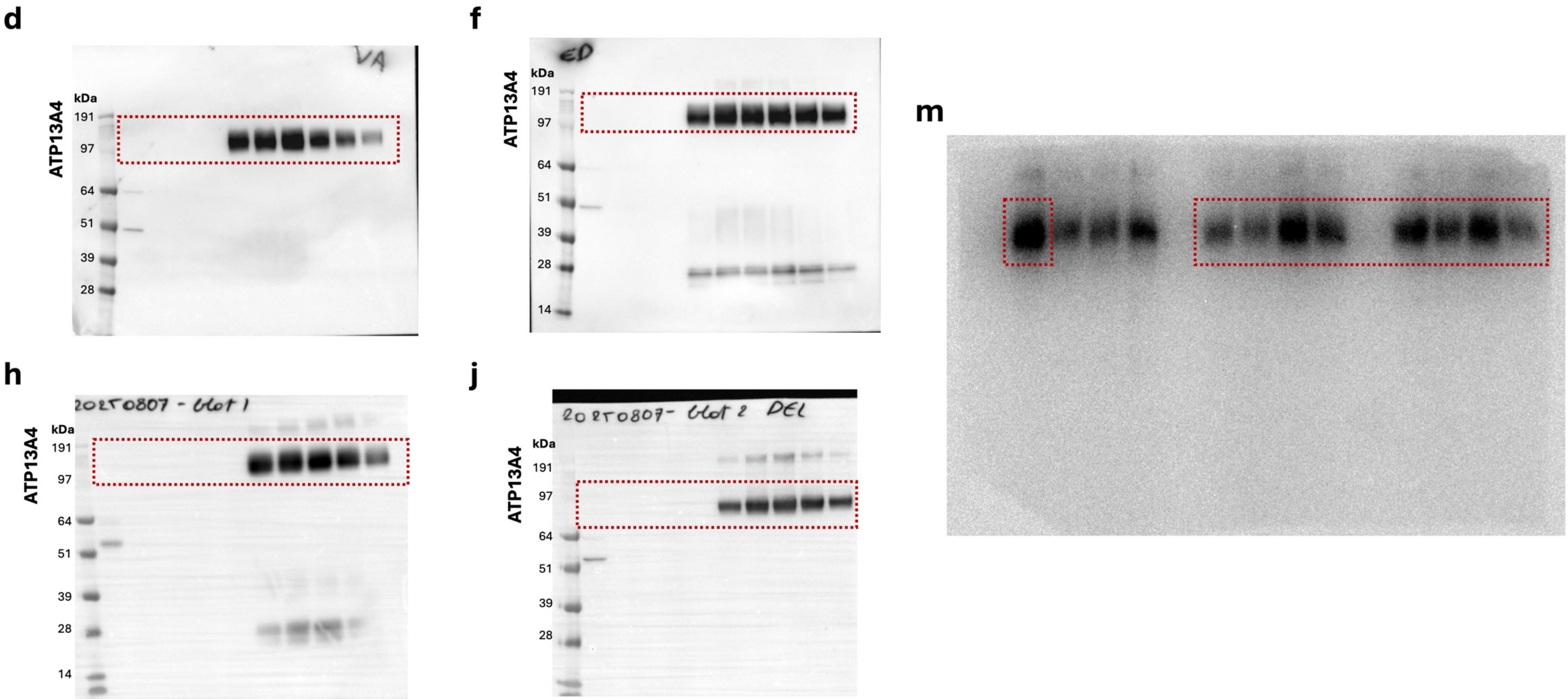

Supplementary Fig. 8: Validation of the *Atp13a4* knockout mouse model.

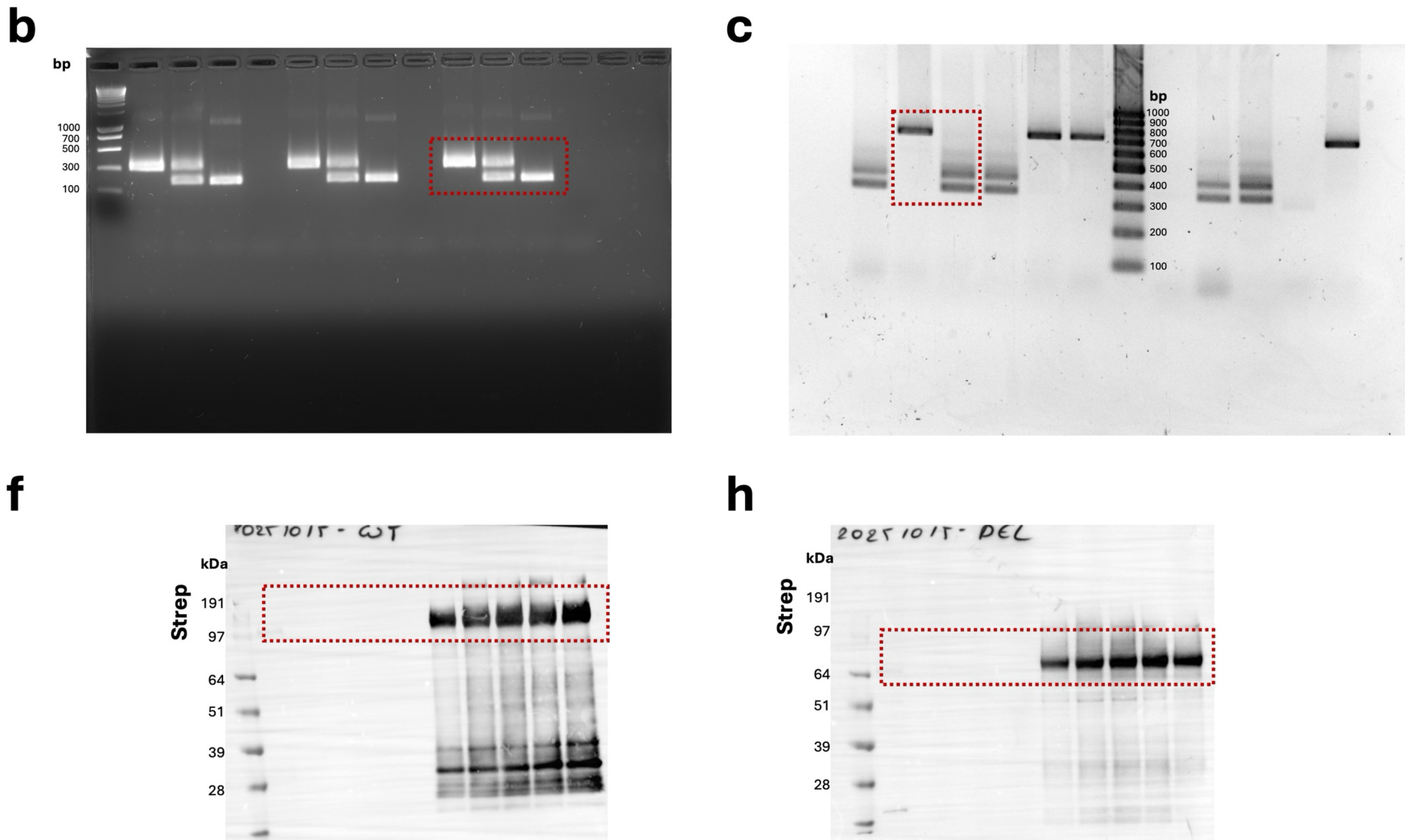

Supplementary Fig. 13: ATP13A4 loss induces selective adaptations in the polyamine pathway without evidence of microglial activation.

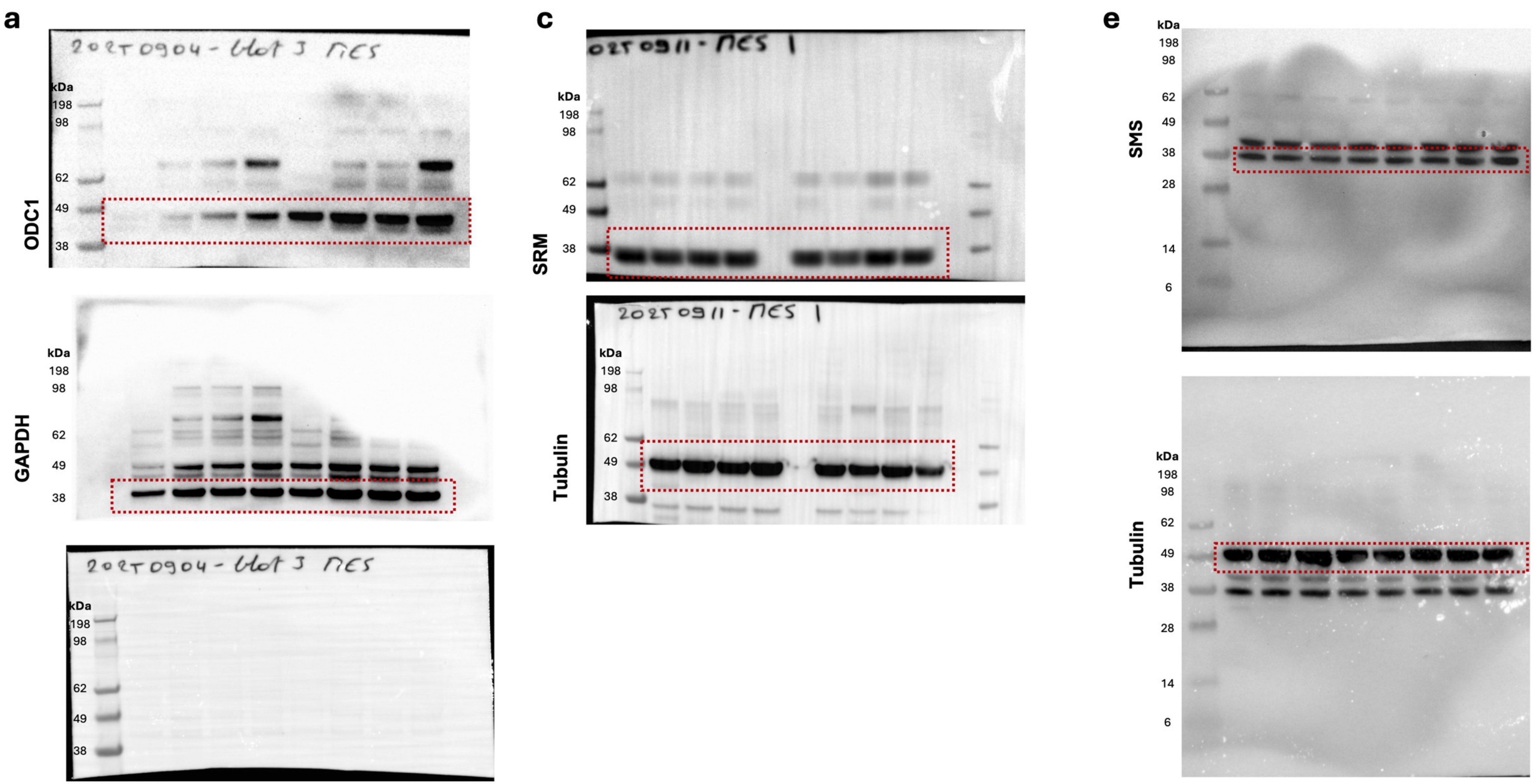

g

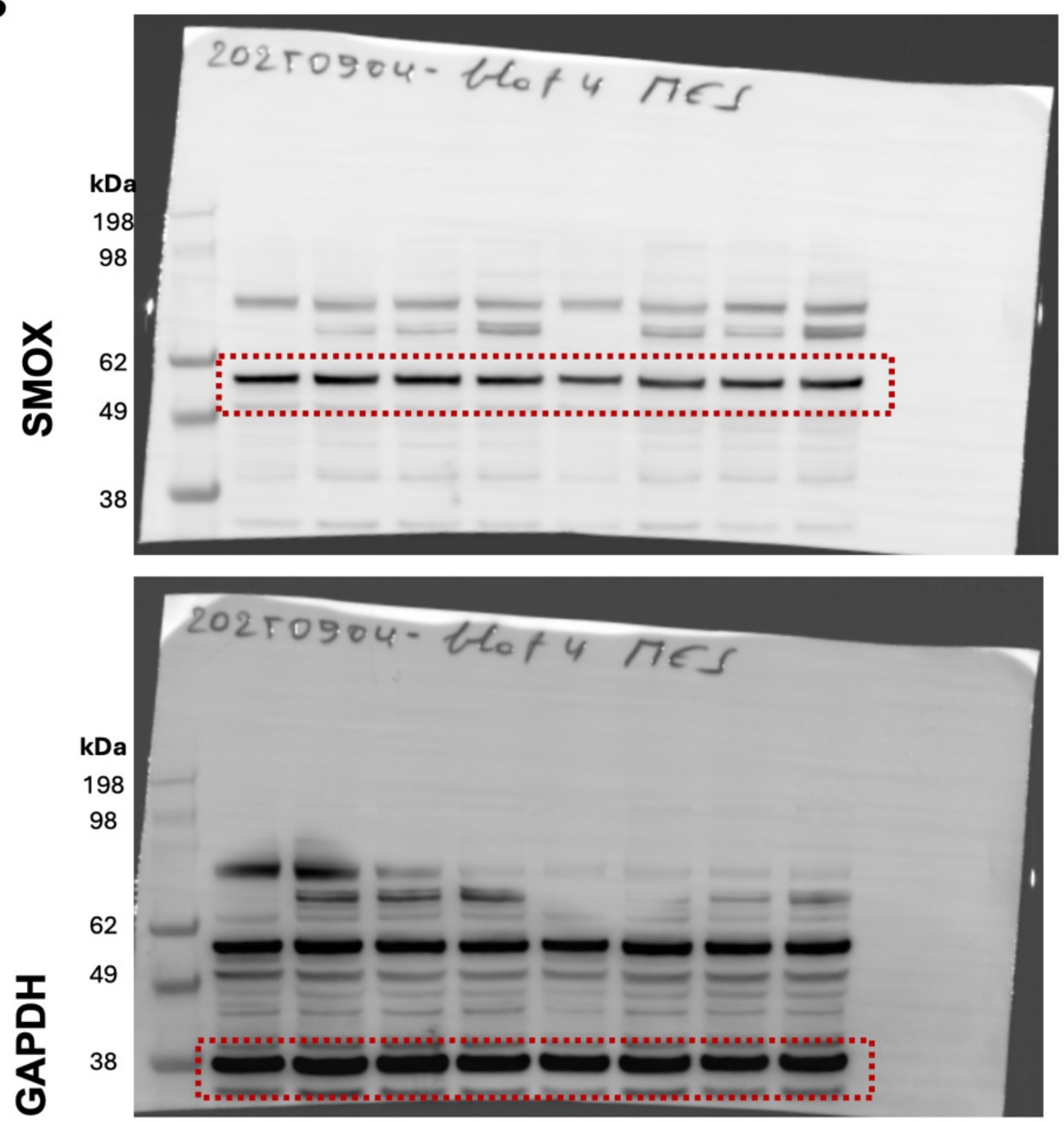
kDa
198
98
62
49
38
SMOX
kDa
198
98
62
49
38
GAPDH

i

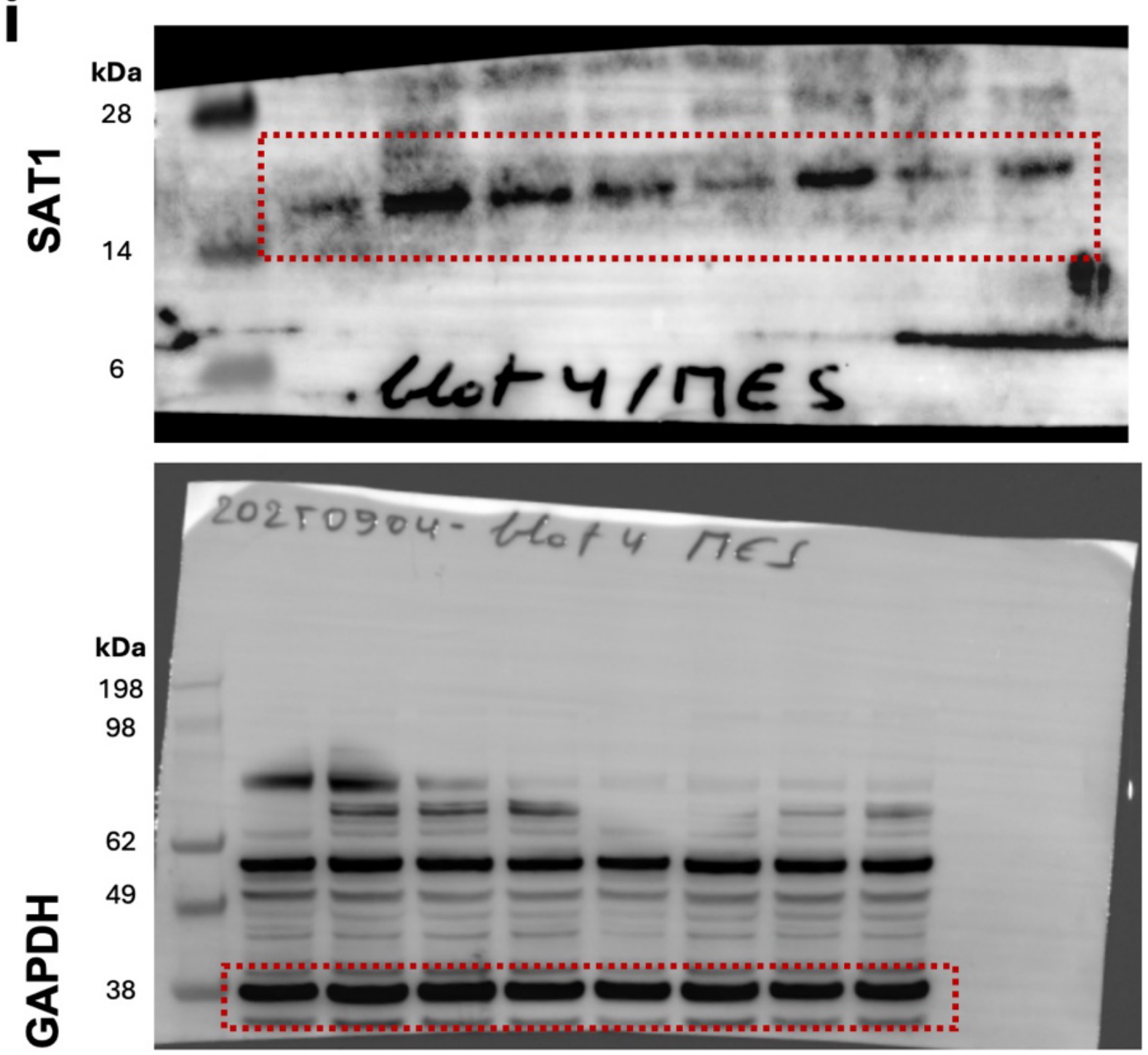
kDa
28
14
6
SAT1
kDa
198
98
62
49
38
GAPDH

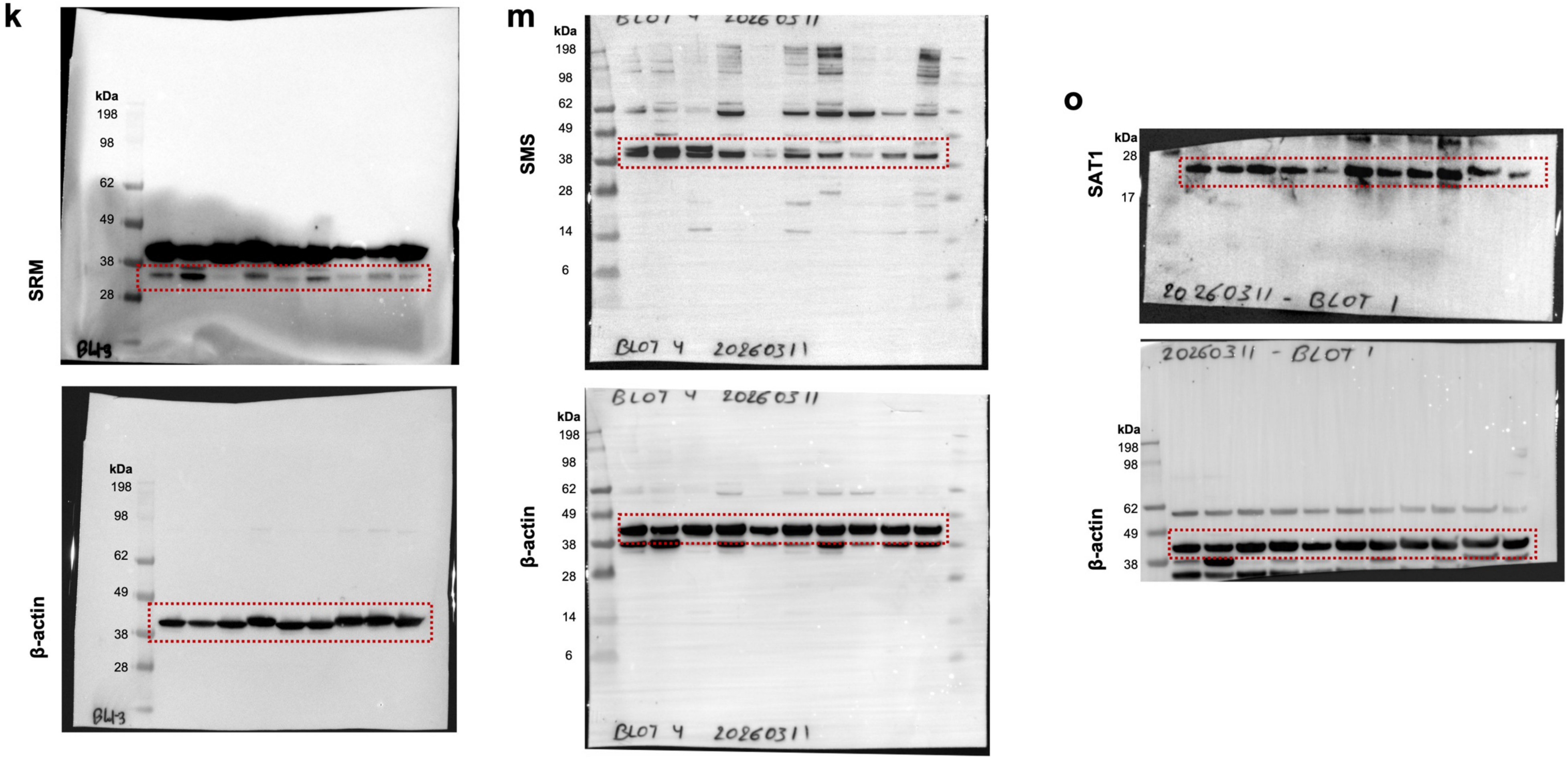
k
kDa
198
98
62
49
38
28
SRM
β-actin
m
SMS
β-actin
14
6
BLOT 4 20260311
o
SAT1
17
20260311 - BLOT 1
