# Supplementary material for: ATP13A4 gates extracellular polyamine levels to control excitatory synaptogenesis
Source: Nat Commun. 2026 Aug 4;17:9395. doi: 10.1038/s41467-026-76132-z (PMC13534584; doi:10.1038/s41467-026-76132-z)
Supplement: Supplementary file 2 [file 41467_2026_76132_MOESM2_ESM.pdf]

Description of Additional Supplementary Files

Supplementary Data 1. Raw peptide intensity data before filtering and normalization for TurboID.

Supplementary Data 2. Peptide intensity data after imputation of missing values for TurboID.

Supplementary Data 3. Normalized peptide intensity data for TurboID.

Supplementary Data 4. Differentially abundant proteins between experimental groups.

Supplementary Data 5. Detailed list of protocols, datasets, code/software, reagents, genetically modified mouse strains and cell lines used in this study.
