# Supplementary material for: ATP13A4 gates extracellular polyamine levels to control excitatory synaptogenesis
Source: Nat Commun. 2026 Aug 4;17:9395. doi: 10.1038/s41467-026-76132-z (PMC13534584; doi:10.1038/s41467-026-76132-z)
Supplement: Supplementary file 4 [file 41467_2026_76132_MOESM9_ESM.pdf]

nature portfolio

Peer Review File

ATP13A4 gates extracellular polyamine levels to control excitatory synaptogenesis

Corresponding Author: Dr Sarah van Veen

This file contains all reviewer reports in order by version, followed by all author rebuttals in order by version.

Version 0:

Reviewer comments:

Reviewer #1

(Remarks to the Author)
In this article the role of ATP13A4 in astrocytes is examined. The authors demonstrate that ATP13A4 is a transporter of polyamines in astrocytes and further show that loss of ATP13A4 influences astrocyte morphogenesis in the developing cortex and excitatory synapse formation in vitro and in development. Finally, they use a germline KO of ATP13A4 to show that dysregulation of polyamine transport can impact mouse behavior in the adult. They find a few variants of ATP13A4 associated with intellectual disability and autism, and show that these variants are defective in polyamine transport. Overall, this is potentially an interesting paper that sadly fails to live up to its billing. The studies are certainly interesting and provocatively presented, but are lacking depth and rigor, which calls into question many of the results. Moreover, important mechanisms are lacking, particularly in regards to the observed sex differences and molecular explanations for how defects in polyamine uptake can specifically influence excitatory synapse formation. Below are experimental suggestions that I hope the authors will consider as they revise a potentially interesting manuscript.

1) I remain completely unconvinced about the specificity of ATP13A4 expression in astrocytes. The data in Fig 1C is specious and unconvincing, also its just in the developing cortex. Their analysis of behavior in Fig 4 is with a germline KO and therefore, they need to show that this gene is specific to astrocytes throughout the brain, in multiple brain regions. Supplemental Figure 1 is from various scRNA-Seq datasets and is not convincing, i.e. they show mouse data from P7 cortex that shows various levels of expression of this gene in nearly every lineage in the brain. Notably, all these data are RNA based, some protein analysis is necessary, along with more rigorous demonstration of the astrocyte-specificity of this gene throughout the brain.
2) In Fig.2i they show that shRNAi knockdown of ATP13A4 yields reduced morphological complexity of astrocytes. This is not the ideal experiment, as it’s known that shRNAi knockdown experiments in astrocytes can often give results that differ from the gold standard KO (as witnessed by the recent neuroligin paper, Golf, et al. 2025). Accordingly, the authors have the ATP13A4-KO mouse line, therefore, they should repeat this experiment in the KO mouse line. Perhaps using in utero electroporation approaches used in their prior studies or sparse labeling with viral approaches. Either way, the authors need to get away from the flawed in vitro, shRNAi approaches and use the optimal tools to demonstrate this point.
3) The in vitro studies in Fig.3 nicely show that polyamine levels can influence excitatory synapse number, linking dysregulation of ATP13A4 to extracellular polyamine levels. However, mechanistic insight into how polyamines selectively influence excitatory synapses is lacking. The authors need to provide some molecular explanation for this, as this is a potentially interesting result. Do polyamine interact with NMDA or AMPA receptors? Do they influence clustering of these receptors? What is the nature of their interactions with excitatory synapses? This question is paramount
4) The behavior studies in Figure 4 are fundamentally flawed as the authors use a germline KO line, when the gold standard for any astrocyte gene is to selectively knockout in astrocytes using Aldh1l1-CreER in combination with a floxed allele, or alternatively use some conditional dCas9/gRNA method. This goes back to point 1, where the specificity of ATP13A4 has not been definitely demonstrated. Given this, its very difficult to interpret the behavioral results in Figure 4, as this phenomenon could be the result of loss of ATP13A4 in other lineages—either during development or in the adult. Some sort of conditional knockout experiment needs to be included in these behavioral studies.
5) Also, the behavioral assays are not convincing at all, very subtle defects, in a sex specific manner. There are to issues that arise from this. First, I question the statistics. They need to employ multiplicity adjusted statistical analysis to account for the number of tasks performed, as these subtle differences could simply be by chance. Second, there are no explanations offered for the sex differences. Do the astrocytes from male or females from ATP13A4 KO mice show differences in morphology or capacity to promote excitatory synapses? This needs to be further explored, because if male astrocytes

demonstrate alterations in synapse formation and morphology, but not effect on behavior, then these phenomenon cannot fully explain the behavioral deficits. There is a clear disconnect between the behavior data and the cellular phenotypes that needs further explanation.
6) The authors rely on loss of function approaches. What happens when they overexpress ATP13A4? Is astrocyte morphology enhanced and excitatory synapse formation reduced? Such complementary manipulations should be explored, at least in the in vitro setting.
7) The variants associated with autism and intellectual disability are interesting, but the studies with the variants is quite thin. The biochemical studies suggest that these variants function as a loss of function. To prove this, the author should overexpress the variants in the context of KO and assess rescue of core synaptic or morphological phenotypes. It is entirely possible that these variants are hypomorphs or even neomorphs, and they might observe some degree of rescue or even a new function. The analysis of the variants, while tantalizing is incomplete.
8) The variants are associated with intellectual disability and are proposed to be loss of function alleles. Yet KO of the gene has as very mild defect on learning (water maze) and has as very subtle (and questionable) seizure phenotype. These disconnects need further EXPERIMENTAL explanation, as they make this manuscript very difficult to follow.
9) The claims of neurodevelopmental defects with the KO analysis are overblown, as nothing in the Fig4 behavior assays suggests a neurodevelopmental defect.
10) The ephys studies in Fig.3 are presumably performed in developing cortex, yet the behavior studies are performed at later stages. Thus, do the defects in astrocyte morphology and excitatory synaptic activity persist into adulthood (when the behaviors are performed) or are these phenotypes developmental in nature and resolve during maturation into adulthood. This issue needs to be clarified.

Reviewer #2

(Remarks to the Author)
The manuscript "Astrocytes fine-tune excitatory synaptic development via polyamine transport" by Sarah van Veen and colleagues describes ATP13A4 as an astrocyte-specific polyamine transporter. The team uses orthogonal approaches to 1) validate astrocyte-specific Atp13a4 expression; 2) demonstrate subcellular localization of Atp13a4 in late endosomes/lysosomes; 3) confirm substrate specificity of polyamines in stimulating ATPase activity; 4) demonstrate requirement of Atp13a4 for astrocytic polyamine uptake; 5) identify a role for Atp13a4 and polyamines in establishing efficient astrocyte morphology and excitatory synapses; 6) document reduced Atp13a4 functionality in purified protein harboring disease-associated variants; and 7) describe sex-specific neurodevelopmental deficits in a mouse model associated with human pathology. The manuscript is very well written, locally structured, and presents several new findings that expand what is known about polyamine transport in the brain. As polyamines are becoming increasingly associated with neurodevelopment, the results of this study will be of value across disciplines. I have a few suggestions, mostly minor, for improvement:
The manuscript overall gives the impression that polyamine biosynthesis/interconversion is inactive in astrocytes. Data presented in figure S4p indicate a loss of endogenous intracellular spermidine with Atp13a4 KD, presumably due to decreased uptake. However, spermidine is not generally detectable in culture medium, suggesting additional effects either on the pathway or export. Similarly, Fig. 3D indicates more extracellular SPD, which is referenced as "endogenous," but no analysis of non-conditioned medium is included. This would be informative in determining the source of spermidine, compared with WT, supporting decreased uptake or potentially increased excretion. Additionally, quantification of extracellular N1-acetylated SPD in the ACM may be informative. As it stands, ATP13A4 certainly plays a role in transport, but the polyamine data suggest there might be more to the story. The discussion and conclusions should reflect this.
Also regarding 3d and e: if the value of WT ACM actually equals that of non-conditioned medium, it would alleviate the discrepancy in concentration between 3d and 3e
Fig S5, astrocyte morphology study and synapse formation: the fact that signal changes are observed with as little as 10 nM polyamine is notable and should be emphasized for its physiological relevance (but also because this experiment is a longer treatment time in the absence of aminoguanidine or SFM; heat inactivation does not eliminate BSAO activity)
Minor suggestions:
Figure 1c: msAtp13a4 puncta are not visible; please provide a more convincing image
Lines 150-151 state that msAtp13a4 is enriched within GFP-positive astrocytes, but it is unclear to what this enrichment is compared to. Were puncta also measured in neurons?
Fig. 1j: This schematic is very useful but could be improved by defining the orange and green dots in the figure legend.
Are the sex-specific effects observed in mice also noted in human patients?
The idea that extracellular spermidine is part of the pathology is very interesting. Can it be measured in vivo? Could it be detected in CSF? Is there increased spermidine in peripheral blood of the mouse model?

Reviewer #3

(Remarks to the Author)
The study identifies ATP13A4, a P5B-type ATPase highly enriched in astrocytes, as the principal transporter that internalizes extracellular polyamines and maintains their homeostasis. ATP13A4 loss in cultured astrocytes or knockout mice reduces astrocytic arbor complexity, heightens excitatory synaptogenesis, and produces mild, sex-specific behavioral phenotypes; human loss-of-function variants produce similar defects, firmly linking disturbed astrocytic polyamine transport to neurodevelopmental pathology. Exogenous spermidine phenocopies the knockout, underscoring astrocytes' pivotal role in clearing excess polyamines to sculpt synapse formation.

This work provides the first mechanistic bridge between glial polyamine handling and excitatory synapse development, spotlighting astrocytes (not neurons) as critical regulators of circuit maturation. By uniting molecular, cellular, animal, and human genetic data, it opens a new therapeutic avenue for neurodevelopmental disorders rooted in disrupted astrocyte physiology. The presented data are comprehensive and largely convincing, and addressing the following points would strengthen the manuscript.

1. In Figure 1c the msAtp13a4 in-situ hybridization signal is barely discernible. It would be helpful to know whether this faint appearance reflects genuinely low transcript levels in the sampled tissue or instead arises from technical factors such as probe efficiency, imaging settings, or overall image quality.

2. The TurboID data in Figures 1e–i place ATP13A4 and its paralog ATP13A3 in close proximity to the endo-/lysosomal marker LAMP1, yet the conventional fluorescence images in Supplementary Figure 1 (particularly panels 1a, 1c, and 1f) do not show obvious colocalization between ATP13A4 and LAMP1. A brief explanation that reconciles these observations, perhaps by highlighting methodological sensitivities or temporal differences captured by TurboID, would greatly clarify the interpretation of ATP13A4's subcellular localization.

3. Although ATP13A4 shares ~37 % sequence identity with the well-characterized polyamine transporter ATP13A2, supporting the authors' hypothesis that it moves polyamines, this modest similarity also leaves room for additional substrates. It may be helpful to explore whether ATP13A4 can transport other metabolites alongside polyamines.

4. The current data leave the directionality of ATP13A4-mediated polyamine transport unclear. If ATP13A4 resides at the plasma membrane, it could plausibly account for the polyamine retention noted in Fig. 1p ,q and Supplementary Fig. 1g ,h. Conversely, if the transporter is restricted to lysosomes in astrocytes, the pathway by which it elevates cytosolic—or lowers extracellular—polyamine levels remains obscure. Because this point bears directly on the study's main conclusions, additional evidence is needed to clarify ATP13A4's subcellular localization and transport direction.

5. The proposed two-step model hinges on both endocytic uptake of extracellular polyamines and ATP13A4-mediated release from endosomes/lysosomes into the astrocytic cytosol. Could the authors clarify whether ATP13A4 actively contributes to the initial endocytic process, or functions solely as an intracellular exporter once polyamines are internalized? Clearer information on transport directionality—and a revised version of Fig. 1j to reflect this—would help readers follow the model more easily.

6. The manuscript attributes abnormal astrocyte function to ATP13A4 deficiency, as suggested in Fig. 2h-j. However, astrocyte morphology in Atp13a4 knockout mice is not directly shown. Such data would strengthen the claim that ATP13A4 loss leads to astrocytic dysfunction

7. The authors propose in Figs 3 and 4 that elevated extracellular polyamines (e.g., spermidine) cause synaptic and behavioral abnormalities. To establish causality, the authors should test whether polyamine levels were indeed increased or lowering polyamine levels rescues these phenotypes. This would strengthen the proposed mechanistic link.

8. The evidence for an anxiolytic phenotype in female KO mice rests largely on 30-s time-bin analyses, yet the full open-field center-time trace (Fig. 4f) shows only a modest WT–KO difference. Complementary assays such as the elevated plus-maze or light–dark box would provide a more comprehensive assessment of anxiety-related behavior. Likewise, the PTZ challenge was performed at 25 mg/kg, a dose that may be too low to elicit reliable seizure activity; alternative metrics (e.g., seizure-severity scoring across a broader dose range) could strengthen the conclusions.

9. The manuscript centers on astrocytic arborization while largely dismissing neuronal morphology, citing "no significant differences in neurite elongation" after ACM treatment (Supplementary Fig. 6a–b) to claim ATP13A4 is dispensable for dendritic growth. This rests on the untested premise that ATP13A4 is exclusive to astrocytes, yet the documented increase in excitatory synapse number and mEPSC frequency suggests neuronal branching is probably altered. Two methodological issues compound the concern: (i) ACM treatment assumes astrocytes are the sole regulators of extracellular polyamines and that spermidine accumulates in the medium; direct polyamine application would provide a clearer test; (ii) all data are in vitro, despite the availability of ATP13A4-KO mice. Demonstrating astrocytic and neuronal morphology in vivo would greatly strengthen the authors' conclusions.

Version 1:

Reviewer comments:

Reviewer #1

(Remarks to the Author)
The authors have done an excellent job in responding to my critiques.
I only have on further suggestion. The authors should consider placing Reviewer Figure 1, which shows that the pathogenic variants do not rescue morphological defects in the main paper. Its a nice finding that reinforces, at the cellular level, that the variant functions as a loss of function.

Reviewer #2

(Remarks to the Author)
The revised version of this manuscript appropriately addressed my concerns. I recommend acceptance for publication.

Reviewer #3

(Remarks to the Author)
The authors substantially revised their manuscript to address my review comments by incorporating new in vivo metabolomic data, primary knockout astrocyte analyses, and comprehensive developmental behavioral assessments to corroborate the mechanistic link between ATP13A4-dependent astrocytic polyamine regulation and excitatory synapse development. I have no additional comments.

Open Access This Peer Review File is licensed under a Creative Commons Attribution 4.0 International License, which permits use, sharing, adaptation, distribution and reproduction in any medium or format, as long as you give appropriate credit to the original author(s) and the source, provide a link to the Creative Commons license, and indicate if changes were made.
In cases where reviewers are anonymous, credit should be given to 'Anonymous Referee' and the source.
The images or other third party material in this Peer Review File are included in the article's Creative Commons license, unless indicated otherwise in a credit line to the material. If material is not included in the article's Creative Commons license and your intended use is not permitted by statutory regulation or exceeds the permitted use, you will need to obtain permission directly from the copyright holder.
To view a copy of this license, visit https://creativecommons.org/licenses/by/4.0/

We thank the reviewers for their constructive and insightful evaluation of our manuscript. In response, we have substantially revised the study by incorporating multiple new experimental datasets, additional analyses, and a refined conceptual framework that directly address the major concerns raised.

The revised manuscript substantially strengthens the original submission by addressing key limitations and establishing a mechanistic and physiological link between ATP13A4 function, extracellular polyamine homeostasis, and excitatory synaptogenesis. Most importantly, we now provide **direct *in vivo* evidence that ATP13A4 loss disturbs polyamine compartmentalization in the brain**. Targeted metabolomic analyses in *Atp13a4* KO mice reveal a clear redistribution of polyamines, with selective depletion in cortical tissue and accumulation in cerebrospinal fluid. These findings establish a physiological link between ATP13A4 function and extracellular polyamine homeostasis, supporting a model in which ATP13A4-mediated uptake contributes to constraining extracellular polyamine availability.

In addition, we substantially expanded the *in vivo* phenotypic characterization by incorporating a comprehensive developmental milestone assessment. *Atp13a4* KO mice exhibit **robust and reproducible delays in early postnatal development**, including delayed eye opening and significant impairments in multiple sensorimotor milestones such as rooting reflex and forelimb grasp. Importantly, these developmental phenotypes were observed similarly in male and female mice, indicating that the early neurodevelopmental effects of ATP13A4 loss are largely sex independent. These findings substantially strengthen the developmental and *in vivo* relevance of the study.

At the molecular level, we further demonstrate that ATP13A4 loss induces coordinated changes in polyamine metabolism, including upregulation of the polyamine synthesis enzyme ODC1 and downregulation of the catabolic enzyme SMOX in brain tissue and astrocytes, indicating a compensatory shift toward a polyamine-conserving state. Together, these findings reveal a multi-level disruption of polyamine homeostasis in *Atp13a4* KO mice.

In parallel, we strengthened the mechanistic framework linking astrocytes to synapse regulation. By integrating astrocyte-conditioned medium experiments with quantitative metabolomics and defined spermidine supplementation, we provide evidence that **extracellular spermidine functions as a synaptogenic cue**. Additional controls exclude altered polyamine catabolism as the primary underlying mechanism, supporting a model in which impaired ATP13A4-mediated uptake elevates extracellular spermidine and enhances excitatory synaptogenesis.

To further support the astrocytic contribution, we extended our cellular analyses using primary astrocytes derived from *Atp13a4* KO mice, demonstrating that loss of ATP13A4 is sufficient to impair astrocyte morphogenesis. These findings strengthen the evidence for a cell-autonomous role of ATP13A4 in astrocyte development.

Finally, we strengthened the evidence for ATP13A4 expression in glial cells by incorporating multiple single-cell and single-nucleus transcriptomic datasets alongside TurboID-based

proteomics, providing a comprehensive and consistent characterization of its glia-enriched, astrocyte-predominant expression profile.

The strength of the revised study lies in the convergence of biochemical, cellular, *in vivo*, and human genetic evidence, providing an integrated characterization of ATP13A4 function and its role in neurodevelopment. Collectively, **these revisions establish a coherent framework linking ATP13A4-dependent polyamine regulation to altered excitatory synapse development and directly address the major concerns raised by the reviewers**. The study identifies extracellular spermidine as a synaptogenic cue and supports a direct link between polyamine transport and neurodevelopmental circuit formation, thereby substantially strengthening the conceptual advance of the work. Although future cell type-specific genetic approaches will further refine the relative contributions of astrocytic versus non-astrocytic ATP13A4, the current cellular and *in vivo* data provide complementary mechanistic and physiological evidence supporting the main conclusions of the study.

Below, we summarize the principal revisions and respond to each reviewer comment in detail.

Principal revisions

1. *In vivo* validation of polyamine compartmentalization (NEW)

We now provide direct *in vivo* evidence that ATP13A4 loss disrupts brain polyamine homeostasis (**Fig. 5a-g**). Targeted LC-MS/MS metabolomics reveals compartmental redistribution of polyamines, with selective depletion in cortical tissue and accumulation in cerebrospinal fluid. These findings support a model in which ATP13A4-mediated uptake contributes to regulating extracellular polyamine availability in the brain and reinforce our mechanistic and cellular findings *in vitro*.

2. Neurodevelopmental phenotype substantially strengthened (NEW)

In response to the relatively modest adult behavioral phenotype observed in *Atp13a4* KO mice, we expanded the *in vivo* analysis to include early developmental stages (**Fig. 6a-k**). We now include a comprehensive developmental milestone assessment demonstrating delayed early postnatal development, including delayed eye opening and significant impairments in rooting reflex and forelimb grasp after Bonferroni correction. Importantly, these developmental phenotypes were observed similarly in male and female mice, indicating a largely sex-independent early neurodevelopmental phenotype. Together, these data extend the *in vivo* characterization of *Atp13a4* KO mice from subtle adult behavioral alterations to an early developmental phenotype consistent with altered circuit maturation. We also revised the text to explicitly frame these findings as global KO phenotypes and discuss conditional astrocyte-specific models as an important future direction.

3. Mechanistic link between polyamine homeostasis and synaptogenesis strengthened (NEW)

Our revised study strengthens the mechanistic link between ATP13A4-dependent polyamine homeostasis and excitatory synaptogenesis. We demonstrate that ATP13A4 influences both intracellular polyamine balance and extracellular polyamine availability, with loss of ATP13A4 leading to elevated extracellular spermidine. By integrating astrocyte-

conditioned media experiments with quantitative metabolomics and defined spermidine supplementation, we identify extracellular spermidine as a synaptogenic cue (**Fig. 4d-f**). In parallel, we show that ATP13A4 deficiency induces coordinated metabolic adaptations (**Fig. 5i-l**), including upregulation of ODC1 and downregulation of SMOX in brain tissue and astrocytes, consistent with a compensatory response to altered intracellular polyamine availability. Together, these findings reveal a broader disruption of polyamine homeostasis and support a mechanistic framework linking polyamine dysregulation to enhanced excitatory synaptogenesis.

4. Validation of astrocyte morphology defects in knockout models (NEW)

We extended our cellular analyses to primary astrocytes derived from *Atp13a4* KO mice and demonstrate that loss of ATP13A4 is sufficient to impair astrocyte morphogenesis (**Fig. 3d-e**). These findings complement prior knockdown and *in vivo* PALE experiments and strengthen the evidence for a cell-autonomous role of ATP13A4 in astrocyte development.

5. Strengthened evidence for glia-enriched expression with astrocyte predominance

We strengthened the evidence for ATP13A4 expression using multiple sc/snRNA-seq datasets (**Supplementary Fig. 1-2**) and TurboID proteomics (**Fig. 1e-i**), which collectively demonstrate glia-enriched and astrocyte-predominant ATP13A4 expression. The terminology throughout the manuscript has been refined accordingly to reflect this expression pattern and to distinguish subtle differences between expression in mouse and human brain cell types.

6. Functional characterization of human ATP13A4 variants expanded

We strengthened the link with human disease by including biochemical analysis of additional patient-associated variants (E276K, C-terminal deletion), which exhibit impaired ATP13A4 function consistent with a loss-of-function mechanism in neurodevelopmental disorders (**Fig. 2i**).

Below, we provide detailed, point-by-point responses to each of the reviewer's comments and outline the revisions made to address these concerns.

<u>Reviewers' comments:</u>

<u>Reviewer #1 (Remarks to the Author):</u>

In this article the role of ATP13A4 in astrocytes is examined. The authors demonstrate that ATP13A4 is a transporter of polyamines in astrocytes and further show that loss of ATP13A4 influences astrocyte morphogenesis in the developing cortex and excitatory synapse formation in vitro and in development. Finally, they use a germline KO of ATP13A4 to show that dysregulation of polyamine transport can impact mouse behavior in the adult. They find a few variants of ATP13A4 associated with intellectual disability and autism, and show that these variants are defective in polyamine transport.

Overall, this is potentially an interesting paper that sadly fails to live up to its billing. The studies are certainly interesting and provocatively presented, but are lacking depth and rigor, which calls into question many of the results. Moreover, important mechanisms are lacking, particularly in regards to the observed sex differences and molecular explanations for how defects in polyamine uptake can specifically influence excitatory synapse formation. Below are experimental suggestions that I hope the authors will consider as they revise a potentially interesting manuscript.

We thank the reviewer for the careful and critical evaluation of our manuscript and appreciate the recognition that the study addresses an interesting and potentially important question. In response to these comments, we substantially revised the manuscript to improve both the rigor and depth of the work, resulting in stronger support for the main conclusions. In the revised version, we expanded our analyses, clarified data presentation, and refined our interpretations throughout. In particular, we strengthened the experimental support for our central conclusions, provided additional context for the observed sex-specific effects, and further developed the mechanistic framework linking ATP13A4-dependent polyamine uptake to astrocyte function and excitatory synapse development. Specifically, our data support a model in which ATP13A4-dependent polyamine handling influences extracellular polyamine availability and, consequently, excitatory synaptogenesis. This work establishes a conceptual framework for understanding how polyamines shape synaptic function and highlights extracellular polyamine regulation as a key layer of control.

The present study characterizes a previously unstudied, disease-relevant gene using complementary molecular, cellular, and *in vivo* approaches. Given the broad scope of the work, spanning ATP13A4 function, polyamine regulation, cellular phenotypes, and *in vivo* disease-relevant outcomes, we focused on establishing the functional link between ATP13A4-dependent polyamine homeostasis and the phenotypes observed in our experimental models. While additional downstream mechanisms remain important areas for future investigation, the current study provides a framework linking ATP13A4-dependent polyamine regulation to altered astrocyte function and excitatory synapse development. We revised the manuscript accordingly to more clearly discuss potential downstream

mechanisms, including effects on ion channel activity, transcriptional or translational regulation, and cytoskeletal organization.

Below, we provide detailed, point-by-point responses to each of the reviewer's comments and outline the revisions made to address these concerns.

1) I remain completely unconvinced about the specificity of ATP13A4 expression in astrocytes. The data in Fig 1C is specious and unconvincing, also its just in the developing cortex. Their analysis of behavior in Fig 4 is with a germline KO and therefore, they need to show that this gene is specific to astrocytes throughout the brain, in multiple brain regions. Supplemental Figure 1 is from various scRNA-Seq datasets and is not convincing, i.e. they show mouse data from P7 cortex that shows various levels of expression of this gene in nearly every lineage in the brain. Notably, all these data are RNA based, some protein analysis is necessary, along with more rigorous demonstration of the astrocyte-specificity of this gene throughout the brain.

We thank the reviewer for this important comment. To address these concerns, we expanded our analyses, incorporated additional datasets spanning multiple brain regions and developmental stages, and revised our terminology to more accurately describe the expression pattern of ATP13A4. The RNA-FISH data in **Fig. 1c** were intended as spatial validation of the transcriptomic observations, which are supported by multiple independent datasets and complemented by *in vivo* proteomic evidence.

(1) Improved RNA-FISH visualization

We note that the RNA-FISH panel in the originally submitted **Fig. 1c** was inadvertently distorted during file conversion, which reduced image resolution and obscured the *msAtp13a4* mRNA puncta. We apologize for the poor image quality in the submitted version. This issue has now been corrected in the revised figure, and the panels have been enlarged to improve visibility. In response to the reviewer's suggestion, we added a magnified view at P21 to more clearly highlight the *msAtp13a4* mRNA puncta (**Fig. 1c**, rightmost column). The dashed box in the P21 panels indicates the region shown at higher magnification (scale bar, 3 μm). The updated panel includes three independent high-resolution examples spanning P7, P15, and P21. Each developmental stage is shown as merged Aldh1l1-GFP and *msAtp13a4* RNA-FISH images together with enhanced renderings highlighting individual RNA puncta. These images consistently demonstrate *msAtp13a4* RNA puncta within Aldh1l1-positive astrocytes across the developmental stages examined. Additionally, we have revised the quantification in **Fig. 1d** by replacing the absolute puncta counts with a percentage-based representation. Each bar now reflects the proportion of total *msAtp13a4* puncta overlapping with astrocytes versus non-astrocyte cells. This representation more clearly illustrates the astrocyte enrichment of *msAtp13a4* mRNA across cortical layers and developmental timepoints.

(2) Expanded transcriptomic analyses

We extended our transcriptomic analysis by integrating additional publicly available datasets from both human and mouse brain (**Supplementary Fig. 1-2**). Across independent

human and mouse datasets spanning multiple brain regions and developmental stages, ATP13A4 consistently shows glial-enriched expression:

- In human datasets (1-5), ATP13A4 is enriched in astrocytes and oligodendrocyte precursor cells (OPCs), with limited neuronal or microglial expression, and is also detected in specialized glial subtypes such as Bergmann glia (cerebellar astrocytes supporting Purkinje neurons) and choroid plexus epithelial cells (**Supplementary Fig. 1**).
- In mouse datasets (6-9), *msAtp13a4* is consistently enriched in astrocytes across developmental stages, with limited expression in neurons or microglia and only minimal expression in other glial populations (**Supplementary Fig. 2**). Importantly, these datasets include multiple CNS regions, demonstrating that this enrichment is not restricted to the developing cortex. By comparison, *msAtp13a2* and *msAtp13a3* are broadly expressed across neuronal and glial lineages, whereas *msAtp13a5* is largely restricted to vascular-associated cells.

Together, these analyses support ATP13A4 as a glia-enriched P5B-type ATPase with prominent astrocytic expression in mouse brain and a broader glial distribution in humans.

(3) Protein-level validation using TurboID

To address the reviewer's concern that the evidence was primarily RNA-based, we note that the original manuscript already included protein-level validation of ATP13A4 expression in astrocytes. Using an astrocyte-specific *in vivo* proximity biotinylation (TurboID) approach (**Fig. 1e-i; Supplementary Fig. 4**), we detected endogenous Atp13a4 protein enriched within LAMP1-positive astrocytic endo-/lysosomal compartments, whereas Atp13a3 was detected at substantially lower levels. These findings provide direct *in vivo* evidence for endogenous msAtp13a4 protein in astrocytes. While TurboID does not quantify absolute cell-type specificity, it enables robust detection of endogenous protein within defined cellular compartments *in vivo*. Detection of endogenous ATP13A4 by conventional immunostaining remains technically challenging, despite extensive evaluation of both commercial and in-house antibodies, a limitation that has also been reported for other P5B-type ATPases such as ATP13A2 and ATP13A3. In this context, the TurboID-based proteomic approach represents the most robust available strategy to assess endogenous ATP13A4 expression *in vivo* and supports endogenous astrocytic ATP13A4 protein expression.

(4) Revised terminology and interpretation

We revised the manuscript text to clarify our conclusions regarding cell-type specificity. Rather than describing ATP13A4 as strictly "astrocyte-specific," we now refer to it as "glia-enriched," which more accurately reflects the transcriptomic data, particularly in humans. In mouse brain, however, *Atp13a4* expression remains most prominent in astrocytes, with only limited expression detected in other cell populations. This expression pattern provides the rationale for focusing our functional analyses on astrocyte biology while avoiding claims of absolute astrocyte exclusivity.

We further acknowledge that low-level expression in additional glial populations, particularly in human datasets, cannot be excluded. Nevertheless, the prominent astrocytic expression of ATP13A4 in mouse brain, together with the known role of astrocytes as major

polyamine-storing cells in the brain (10-14), supports our focus on astrocyte-mediated polyamine regulation. Our findings address the longstanding question of how astrocytes acquire extracellular polyamines, supporting a role for ATP13A4-mediated uptake in regulating astrocyte morphology and astrocyte-dependent excitatory synaptogenesis, thereby linking astrocytic polyamine handling to neuronal circuit development.

Collectively, these data support a revised conclusion that ATP13A4 is a glia-enriched P5B-type ATPase with prominent astrocytic expression, particularly in mouse brain. Together with the *in vivo* proteomic validation, these findings provide a strong rationale for focusing the present study on astrocyte biology.

2) In Fig.2i they show that shRNAi knockdown of ATP13A4 yields reduced morphological complexity of astrocytes. This is not the ideal experiment, as it's known that shRNAi knockdown experiments in astrocytes can often give results that differ from the gold standard KO (as witnessed by the recent neuroligin paper, Golf, et al. 2025). Accordingly, the authors have the ATP13A4-KO mouse line, therefore, they should repeat this experiment in the KO mouse line. Perhaps using in utero electroporation approaches used in their prior studies or sparse labeling with viral approaches. Either way, the authors need to get away from the flawed in vitro, shRNAi approaches and use the optimal tools to demonstrate this point.

We thank the reviewer for this constructive comment.

We note that the initial shRNA experiments were designed to model partial loss of ATP13A4 function, consistent with the hypomorphic behavior of several patient-associated variants (A356V, E646D, E276K, exon deletion). We clarified this rationale in the revised manuscript.

Importantly, we have now directly addressed the reviewer's concern by performing morphological analyses in primary astrocytes derived from *Atp13a4* KO mice. When co-cultured with WT neurons, *Atp13a4* KO astrocytes exhibit a clear reduction in arborization and morphological complexity, consistent with the phenotype observed in the shRNA-based knockdown experiments (**Fig. 3d-e**). **These findings demonstrate that loss of Atp13a4 is sufficient to impair astrocyte morphogenesis, thereby validating the original observations obtained using shRNA-based approaches.**

In vivo sparse labeling approaches, such as *in utero* electroporation (IUE) or PALE, could provide additional single-cell morphological resolution in future studies but are technically challenging to implement efficiently in the current *Atp13a4* KO background. To further assess astrocyte alterations *in vivo* within the current study, we performed GFAP immunostaining in *Atp13a4* KO brain sections. This analysis revealed increased GFAP immunoreactivity compared to WT controls (**Fig. 5m-n**), indicative of an altered astrocyte state and consistent with perturbations in astrocyte homeostasis. Although GFAP staining does not resolve fine astrocyte morphology, these findings provide complementary *in vivo* evidence for astrocyte alterations following ATP13A4 loss.

Together, these findings demonstrate that both partial (KD) and complete (KO) ATP13A4 loss converge on a consistent astrocyte morphological phenotype, supporting a role for

ATP13A4-dependent polyamine regulation in astrocyte development and providing genetic validation of the original shRNA-based observations.

3) The in vitro studies in Fig.3 nicely show that polyamine levels can influence excitatory synapse number, linking dysregulation of ATP13A4 to extracellular polyamine levels. However, mechanistic insight into how polyamines selectively influence excitatory synapses is lacking. The authors need to provide some molecular explanation for this, as this is a potentially interesting result. Do polyamine interact with NMDA or AMPA receptors? Do they influence clustering of these receptors? What is the nature of their interactions with excitatory synapses? This question is paramount.

Although further molecular investigation would be of interest, our study was designed to define the role of ATP13A4-dependent polyamine regulation across biochemical, cellular, and *in vivo* levels and establishes a link between extracellular polyamine availability and excitatory synapse formation. Specifically, our data support a model in which ATP13A4-dependent polyamine uptake influences extracellular spermidine availability, thereby modulating astrocyte-dependent excitatory synapse formation. This interpretation is further strengthened by the newly included *in vivo* metabolomic analyses demonstrating compartmental redistribution of polyamines between brain tissue and CSF following ATP13A4 loss (**Fig. 5a-g**).

We agree with the reviewer that some downstream mechanistic questions remain open. Nevertheless, the current study integrates molecular, cellular, and *in vivo* analyses that collectively link ATP13A4-dependent polyamine regulation to the observed cellular and physiological phenotypes. <u>We have revised the manuscript accordingly to discuss potential downstream pathways and relevant future directions more explicitly</u>.

Polyamines such as spermidine and spermine are known to modulate ionotropic glutamate receptors (NMDA, AMPA) by affecting channel conductance, gating, and rectification (15-18). They also influence postsynaptic receptor clustering and trafficking, as well as local protein synthesis via the eIF5A hypusination pathway (19, 20). Beyond synaptic modulation, polyamines contribute to cytoskeletal remodeling, lysosomal and mitochondrial function, and metabolic regulation, which may indirectly impact synapse formation. Defining the relative contribution of these mechanisms will be an important direction for future studies building on the findings presented here.

4) The behavior studies in Figure 4 are fundamentally flawed as the authors use a germline KO line, when the gold standard for any astrocyte gene is to selectively knockout in astrocytes using Aldh1l1-CreER in combination with a floxed allele, or alternatively use some conditional dCas9/gRNA method. This goes back to point 1, where the specificity of ATP13A4 has not been definitely demonstrated. Given this, its very difficult to interpret the behavioral results in Figure 4, as this phenomenon could be the result of loss of ATP13A4 in other lineages—either during development or in the adult. Some sort of conditional knockout experiment needs to be included in these behavioral studies.

We used a germline *Atp13a4* KO model as a physiologically and translationally relevant system reflecting the systemic genetic context of human ATP13A4 deficiency. We acknowledge that the use of a germline KO limits the ability to attribute the observed behavioral phenotypes exclusively to astrocytic ATP13A4 loss, and <u>we have clarified this point in the revised manuscript</u>.

Our expanded expression analyses, including multiple sc/snRNA-seq datasets (**Supplementary Fig. 1-2**), demonstrate that msAtp13a4 expression is strongly enriched in astrocytes in mouse brain, with only limited expression detected in other cell populations (see response to **Reviewer 1**, **Q1**). Together with the astrocyte-specific cellular phenotypes described in the current study, these findings support a prominent contribution of astrocytic ATP13A4 to the developmental and synaptic phenotypes observed *in vivo*.

We agree that future conditional or inducible approaches will be important to further dissect cell type-specific contributions to the behavioral and synaptic phenotypes identified here. <u>We therefore revised the framing of the manuscript to explicitly interpret the behavioral findings as consequences of global ATP13A4 loss rather than astrocyte-restricted deletion</u>. Within this context, **the current germline KO model provides an informative <u>first</u> *in vivo* system to study how ATP13A4-dependent polyamine dysregulation impacts brain development, polyamine homeostasis, and neurobehavioral phenotypes**.

5) Also, the behavioral assays are not convincing at all, very subtle defects, in a sex specific manner. There are to issues that arise from this. First, I question the statistics. They need to employ multiplicity adjusted statistical analysis to account for the number of tasks performed, as these subtle differences could simply be by chance. Second, there are no explanations offered for the sex differences. Do the astrocytes from male or females from ATP13A4 KO mice show differences in morphology or capacity to promote excitatory synapses? This needs to be further explored, because if male astrocytes demonstrate alterations in synapse formation and morphology, but not effect on behavior, then these phenomenon cannot fully explain the behavioral deficits. There is a clear disconnect between the behavior data and the cellular phenotypes that needs further explanation.

We thank the reviewer for this comment and agree that the adult behavioral phenotypes observed in *Atp13a4* KO mice are modest. <u>We have revised the manuscript accordingly to describe these findings with appropriate caution</u>.

<u>(1) Early neurodevelopmental phenotype</u>

To directly address the reviewer's concern regarding the absence of a clear neurodevelopmental phenotype, <u>we performed a comprehensive analysis of early postnatal developmental milestones</u>. This revealed reproducible delays across multiple domains, including rooting reflex, forelimb grasp, and eye opening (**Fig. 6a-k**, see response to **Reviewer 1**, **Q9**), demonstrating that *Atp13a4* deficiency impacts early neurodevelopment. These assays capture fundamental aspects of sensorimotor maturation and early circuit formation that precede complex behavioral outputs. Importantly, **these developmental delays were consistently observed across the cohort and were largely sex independent**

(**Supplementary Fig. 13**), indicating a broad and penetrant effect during early brain development. In contrast, adult behavioral phenotypes were more subtle and displayed a female bias (**Fig. 6l-o**, **Supplementary Fig. 14-15**). Together, these findings support a model in which Atp13a4 loss primarily impacts early neurodevelopmental processes in both sexes, whereas adult behavioral manifestations are more subtle and partially sex biased.

(2) Adult behavioral characterization

Although adult behavioral phenotypes were modest, several effects were reproducibly observed across independent assays and were most pronounced in female *Atp13a4* KO mice. To further characterize these phenotypes, we expanded the behavioral analysis by including DigiGait recordings and an expanded PTZ dose-response assessment:

- Using an expanded PTZ dose range (15-45 mg/kg; see also response to **Reviewer 3**, **Q8**) in 1-year-old mice, we did not observe consistent genotype-dependent differences in seizure threshold or severity, indicating that ATP13A4 deficiency does not produce a robust alteration in PTZ-induced seizure susceptibility under the conditions tested.
- We also performed quantitative gait analysis using automated DigiGait recordings (**Fig. 6m-o**). Female *Atp13a4* KO mice exhibited altered hindlimb positioning parameters, consistent with the enhanced rotarod performance observed in female KO animals (**Fig. 6l**), whereas most temporal gait parameters remained unchanged. Together, these findings indicate subtle but reproducible alterations in motor coordination in adult *Atp13a4* KO mice, with the strongest effects observed in females.

(3) Sex differences

Our developmental analyses demonstrate that *Atp13a4* deficiency induces early neurodevelopmental alterations that are largely shared across sexes, as evidenced by consistent delays in postnatal milestones (see response to **Reviewer 1**, **Q9**; **Fig. 6a-k**; **Supplementary Fig. 13**). **These findings suggest that early alterations in circuit maturation occur in both sexes, while sex-dependent modulatory mechanisms influence the extent to which these changes manifest at synaptic and behavioral levels in adulthood.** To further address this point, we performed sex-stratified analyses across multiple readouts, including polyamine metabolomics, excitatory synapse density, and astrocyte reactivity (GFAP immunostaining). While polyamine levels and astrocyte reactivity were comparably altered in both sexes, the increase in excitatory synapse density was more pronounced in female *Atp13a4* KO mice than in males (**Supplementary Fig. 16**), consistent with the female-biased behavioral phenotype observed in adulthood.

(4) Statistical analysis

For the adult behavioral battery, we applied multiplicity-adjusted corrections within each test but not across assays, as each assay probes a distinct behavioral domain (e.g. anxiety-related behavior, motor coordination, learning) and was therefore treated as an independent outcome measure. While this method does not control for experiment-wide error across all assays, global correction across all behavioral assays would substantially reduce sensitivity without necessarily improving interpretability. We clarified this rationale in the Methods section of the revised manuscript.

For the developmental milestone battery (**Fig. 6c-k**), the assays collectively assess a shared biological process, namely postnatal neurodevelopmental maturation, and were therefore treated as non-independent measures. Accordingly, we applied Bonferroni correction across all nine milestone tests. Following correction, rooting reflex (adjusted P = 0.0306) and forelimb grasp (adjusted P = 0.0432) remained statistically significant, whereas other milestones did not survive correction.

6) The authors rely on loss of function approaches. What happens when they overexpress ATP13A4? Is astrocyte morphology enhanced and excitatory synapse formation reduced? Such complementary manipulations should be explored, at least in the in vitro setting.

We thank the reviewer for this valuable suggestion. We would like to clarify that the effects of ATP13A4 overexpression on astrocyte morphology were already included in the original manuscript (**Fig. 3f-g**). These experiments show that overexpression of ATP13A4 in primary astrocytes does not significantly alter arborization or process complexity, indicating that increased ATP13A4 levels are not sufficient to further enhance astrocyte morphology under baseline conditions. These findings are consistent with a model in which ATP13A4 is required for maintaining normal astrocyte morphology through regulated polyamine uptake, whereas overexpression alone is not sufficient to drive additional structural remodeling in the absence of altered extracellular polyamine levels.

7) The variants associated with autism and intellectual disability are interesting, but the studies with the variants is quite thin. The biochemical studies suggest that these variants function as a loss of function. To prove this, the author should overexpress the variants in the context of KO and assess rescue of core synaptic or morphological phenotypes. It is entirely possible that these variants are hypomorphs or even neomorphs, and they might observe some degree of rescue or even a new function. The analysis of the variants, while tantalizing is incomplete.

We respectfully disagree that the functional analysis of the pathogenic variants is thin.

(1) Biochemical evidence for loss of function

We purified all ATP13A4 variants and assessed their activity using complementary biochemical assays (ATPase and EP assays) directly on target. These analyses demonstrate that all pathogenic variants exhibit impaired biochemical properties in the absence of confounding cellular factors that may modulate transporter activity. Importantly, these biochemical findings correlate well with the cell-based polyamine uptake assays in which transporter activity was assessed in cells.

Although additional factors may influence ATP13A4 function in a cellular context, the variants consistently exhibit impaired transporter activity across both biochemical and cellular assays. As described in the original manuscript, the autism-associated p.A356V and p.E646D variants exhibit partial loss of function. Specifically, both variants show reduced phosphoenzyme formation, diminished polyamine-stimulated ATPase activity, and impaired polyamine uptake in H4 cells (**Fig. 2i**; **Supplementary Fig. 6c-e; Supplementary**

Fig. 7k-n). These findings indicate that both variants retain residual activity, consistent with a hypomorphic rather than complete loss-of-function phenotype.

(2) Additional disease-associated variants

To further strengthen our findings, we expanded the analysis to include two additional disease-associated variants identified in this study: the p.E276K missense variant and the exon 19-25 deletion, associated with intellectual disability and epilepsy, respectively. Biochemical analysis of purified proteins (**Supplementary Fig. 7g-j**) demonstrated markedly reduced spermidine-stimulated ATPase activity compared to WT ATP13A4 (**Fig. 2i**), indicating impaired transporter function at the molecular level, independent of cellular context. As these assays directly assess ATP13A4 transport activity, they provide a robust measure of loss of function.

(3) Functional rescue experiments

In response to the reviewer's suggestion, we performed an initial rescue experiment in an *Atp13a4* KO background to assess whether disease-associated variants can restore astrocyte morphological phenotypes. Given the technical variability associated with expression-based rescue experiments in primary astrocytes, we focused on an initial proof-of-principle rescue analysis. Re-expression of WT hATP13A4 restored astrocyte morphological complexity, confirming the specificity of the phenotype. In contrast, the exon 19-25 deletion failed to rescue this defect, consistent with its impaired biochemical activity and supporting a loss-of-function mechanism (**Figure R1**). These findings provide supportive functional evidence that disease-associated variants are impaired in their ability to restore core astrocyte phenotypes, supporting the biochemical evidence for impaired ATP13A4 function.

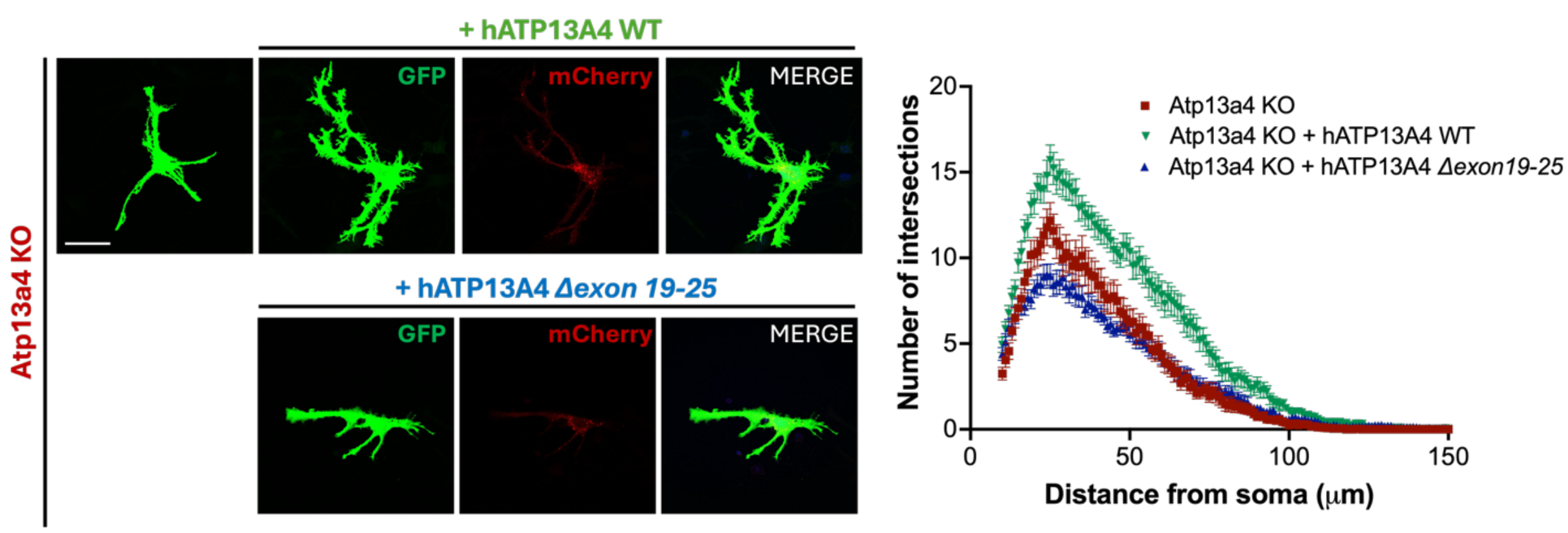

Figure R1. Disease-associated C-terminal deletion fails to rescue astrocyte morphological defects in an *Atp13a4* KO background. Representative images of GFP-transfected *Atp13a4* KO astrocytes (green) in the presence or absence of hATP13A4 constructs (WT or *Δexon19-25*) with an N-terminal mCherry tag (red) after 48 h co-culture with WT neurons. Re-expression of WT ATP13A4 restores astrocyte morphological complexity, whereas the *Δexon19-25* variant fails to rescue this phenotype. Scale bar, 50 µm. Quantification of astrocyte complexity was performed using Sholl analysis.

Together, our results provide convergent biochemical, cellular, and functional evidence that disease-associated ATP13A4 variants impair transporter activity and support a predominantly loss-of-function (including hypomorphic) disease mechanism.

8) The variants are associated with intellectual disability and are proposed to be loss of function alleles. Yet KO of the gene has as very mild defect on learning (water maze) and has as very subtle (and questionable) seizure phenotype. These disconnects need further EXPERIMENTAL explanation, as they make this manuscript very difficult to follow.

We thank the reviewer for this important comment and agree that the adult behavioral and seizure phenotypes observed in *Atp13a4* KO mice are relatively mild. We therefore revised the manuscript to describe these findings with appropriate caution and expanded the *in vivo* characterization by incorporating early developmental analyses.

(1) Adult phenotype

Although adult behavioral phenotypes observed at 8 weeks of age were modest, they were reproducibly observed across multiple assays and accompanied by alterations in brain polyamine homeostasis (**Fig. 5a-g;** see response to **Reviewer 2, Q3**). Mild or context-dependent behavioral phenotypes are common in mouse models of neurodevelopmental disorders, even for genes associated with severe neurological manifestations in humans. For example, *Ocrl1* KO mice lack the major cognitive and neurological features of Lowe syndrome due to compensation by *Inpp5b* (21), *Cdkl5* KO mice show mild behavioral changes and only reveal seizure susceptibility under sensitized conditions (22), while *Scn2a* heterozygous mice display transient social and learning deficits that attenuate with age (23). These examples illustrate that modest behavioral phenotypes in mice do not necessarily preclude disease relevance and may reflect compensatory mechanisms or species-specific differences in neural circuitry.

(2) Developmental phenotype

Importantly, analysis of early postnatal developmental milestones revealed clearer neurodevelopmental alterations in *Atp13a4* KO mice (see response to **Reviewer 1, Q9**). KO animals exhibited delayed acquisition of several milestones, including rooting reflex, forelimb grasp and eye opening, consistent with impaired early postnatal maturation. These assays capture fundamental developmental processes that precede complex behavioral outputs and provide direct evidence that ATP13A4 loss impacts early neurodevelopment.

Together, these molecular, developmental, and behavioral findings support a model in which ATP13A4-dependent polyamine dysregulation perturbs early circuit maturation. Within this framework, the mouse model captures early developmental and synaptic alterations associated with disease mechanisms, even if the full spectrum of human behavioral phenotypes is not reproduced.

9) The claims of neurodevelopmental defects with the KO analysis are overblown, as nothing in the Fig4 behavior assays suggests a neurodevelopmental defect.

We thank the reviewer for this comment and agree that the behavioral assays presented in the original Fig. 4 alone did not provide sufficient evidence for a neurodevelopmental phenotype.

To directly address this concern, we performed a comprehensive analysis of early postnatal developmental milestones in *Atp13a4* KO and WT littermates (**Fig. 6a-k**). These assays assess key aspects of early neurodevelopmental maturation and reflex acquisition. **This analysis revealed reproducible delays in multiple developmental milestones in *Atp13a4*-deficient pups**, with eye opening, rooting reflex and forelimb grasp reaching statistical significance after Bonferroni correction across the nine milestone tests. Additional trends toward delayed acquisition were observed for open field behavior and auditory startle. As these assays capture fundamental developmental processes that precede complex behavioral outputs, these findings provide direct evidence that ATP13A4 loss impacts early neurodevelopment.

Importantly, these developmental delays were observed across the cohort and were not sex dependent. Sex-stratified analyses revealed comparable trends in males and females across assays (**Supplementary Fig. 13**). Although not all comparisons reached statistical significance within individual sex groups, stratification reduced the statistical power available for these subgroup analyses, while the overall effect directions remained similar between sexes. Together, these findings indicate that *Atp13a4* deficiency broadly affects early developmental maturation rather than producing a strictly sex-restricted phenotype.

We incorporated these new developmental data into the revised manuscript and revised the interpretation of the adult behavioral findings accordingly. Together, the results support a model in which ATP13A4 deficiency primarily disrupts early neurodevelopment, whereas adult behavioral phenotypes are more modest and context dependent.

10) The ephys studies in Fig.3 are presumably performed in developing cortex, yet the behavior studies are performed at later stages. Thus, do the defects in astrocyte morphology and excitatory synaptic activity persist into adulthood (when the behaviors are performed) or are these phenotypes developmental in nature and resolve during maturation into adulthood. This issue needs to be clarified.

We acknowledge the reviewers' concern regarding the developmental timing of the electrophysiological recordings in relation to the behavioral assays. To clarify, all electrophysiological recordings were performed in two-month-old WT and *Atp13a4* KO mice, matching the age range of animals used for the adult behavioral studies. This temporal alignment indicates that the observed alterations in excitatory synaptic activity are not restricted to an early developmental window but persist into adulthood.

In the revised manuscript, we now explicitly specify the age of the animals in the figure legends and Methods section and clarify in the Results that the electrophysiological and behavioral datasets were obtained from age-matched adult cohorts. These findings support the conclusion that ATP13A4 loss results in persistent alterations in excitatory synaptic activity rather than a transient developmental phenotype.

Reviewer #2 (Remarks to the Author):

The manuscript "Astrocytes fine-tune excitatory synaptic development via polyamine transport" by Sarah van Veen and colleagues describes ATP13A4 as an astrocyte-specific polyamine transporter. The team uses orthogonal approaches to 1) validate astrocyte-specific Atp13a4 expression; 2) demonstrate subcellular localization of Atp13a4 in late endosomes/lysosomes; 3) confirm substrate specificity of polyamines in stimulating ATPase activity; 4) demonstrate requirement of Atp13a4 for astrocytic polyamine uptake; 5) identify a role for Atp13a4 and polyamines in establishing efficient astrocyte morphology and excitatory synapses; 6) document reduced Atp13a4 functionality in purified protein harboring disease-associated variants; and 7) describe sex-specific neurodevelopmental deficits in a mouse model associated with human pathology.

The manuscript is very well written, locally structured, and presents several new findings that expand what is known about polyamine transport in the brain. As polyamines are becoming increasingly associated with neurodevelopment, the results of this study will be of value across disciplines.

I have a few suggestions, mostly minor, for improvement:

We thank the reviewer for the thoughtful and constructive evaluation of our manuscript. We are very grateful for the positive assessment of our work, particularly regarding its clarity, structure, and **interdisciplinary relevance**. We appreciate the recognition of our findings demonstrating ATP13A4 as a key astroglial polyamine transporter that contributes to intracellular polyamine handling, astrocyte morphology, and excitatory synaptic development, as well as the identification of disease-associated variants with impaired function. Below, we provide detailed responses to each of the reviewer's comments and outline the revisions made to the manuscript.

1) The manuscript overall gives the impression that polyamine biosynthesis/interconversion is inactive in astrocytes. Data presented in figure S4p indicate a loss of endogenous intracellular spermidine with Atp13a4 KD, presumably due to decreased uptake. However, spermidine is not generally detectable in culture medium, suggesting additional effects either on the pathway or export. Similarly, Fig. 3D indicates more extracellular SPD, which is referenced as "endogenous," but no analysis of non-conditioned medium is included. This would be informative in determining the source of spermidine, compared with WT, supporting decreased uptake or potentially increased excretion.

We thank the reviewer for these thoughtful comments and agree that distinguishing between uptake and export mechanisms is important for interpreting the observed increase in extracellular spermidine.

To directly address this point, <u>we performed targeted LC–MS/MS metabolomic analysis of non-conditioned culture medium (NCM)</u>. Under our experimental conditions, spermidine was not detectable in NCM (**Fig. 4d**), demonstrating that the extracellular spermidine observed in astrocyte-conditioned medium (ACM) is not derived from the baseline culture medium but originates from astrocytic activity. This finding supports a model in which

astrocytes contribute to the extracellular polyamine pool and dynamically exchange polyamines with their environment, and that loss of ATP13A4 disrupts this balance, leading to extracellular accumulation. While these data do not fully distinguish between increased release and decreased reuptake, the ACM measurements are not consistent with a model based solely on medium-derived spermidine and instead support altered extracellular polyamine regulation upon ATP13A4 loss. These results have been incorporated into the revised manuscript and are discussed in the context of astrocytic polyamine turnover and intercompartmental redistribution.

2) Additionally, quantification of extracellular N1-acetylated SPD in the ACM may be informative.

We thank the reviewer for this valuable suggestion to assess acetylated polyamine derivatives in ACM.

To address this, we performed targeted LC-MS/MS analysis of N-acetylputrescine, $N^{1/8}$-acetylspermidine and N^{1}-acetylspermine under the same conditions as shown in **Fig. 4d**. N^{1}-acetylspermine was not detectable in ACM, and the levels of N-acetylputrescine and $N^{1/8}$-acetylspermidine did not differ significantly between control and ATP13A4-deficient astrocytes (**Supplementary Fig. 10h-j**). These data indicate that the elevated extracellular spermidine observed upon *msAtp13a4* knockdown is not accompanied by increased acetylation or export of acetylated polyamine derivatives. This argues against enhanced catabolic conversion as the primary mechanism underlying the extracellular spermidine increase. Instead, these findings support a model in which ATP13A4 loss perturbs extracellular polyamine regulation without evidence for increased acetylation-dependent export. These results have been incorporated into the revised manuscript, and the Results and Discussion sections have been updated accordingly to reflect their implications for astrocytic polyamine homeostasis.

3) As it stands, ATP13A4 certainly plays a role in transport, but the polyamine data suggest there might be more to the story. The discussion and conclusions should reflect this.

We thank the Reviewer for this comment. We agree that the initial polyamine data suggested a more complex phenotype than a simple transport defect. To directly address this point, we performed additional experiments to define how ATP13A4 loss affects polyamine homeostasis *in vivo*.

(1) *In vivo* metabolomic analyses

Specifically, we conducted targeted LC-MS/MS metabolomic profiling of cortical brain tissue and cerebrospinal fluid (CSF) from WT and *Atp13a4* KO mice (**Fig. 5a-g, Supplementary Fig. 11h-m**). In cortical tissue, we observed a significant reduction in putrescine levels in KO mice, whereas spermidine and spermine remained unchanged. In addition, the acetylated derivatives $N^{1/8}$-acetylspermidine and N^{1}-acetylspermine were significantly decreased, while N-acetylputrescine was not altered, indicating selective alterations in polyamine metabolism within the brain parenchyma. In contrast, CSF analysis revealed an opposing trend, with increased levels of putrescine and $N^{1/8}$-acetylspermidine

in *Atp13a4* KO mice. Although CSF does not directly measure local interstitial or perisynaptic polyamine concentrations, these findings support altered extracellular polyamine distribution following ATP13A4 loss. Together, the opposing changes observed in cortical tissue and CSF indicate a redistribution of polyamines between intra- and extracellular compartments in the brain.

Importantly, **these new data reinforce our original conclusion that loss of ATP13A4 alters extracellular polyamine homeostasis**. In our initial submission, we proposed that ATP13A4 mediates astrocyte-dependent polyamine uptake, thereby contributing to the regulation of extracellular polyamine levels. The altered CSF polyamine profile observed here provides *in vivo* support for this interpretation. Concurrently, the depletion of specific polyamine species within brain tissue indicates a shift in polyamine partitioning between intra- and extracellular pools. We have included these data in a new **Fig. 5** presenting combined brain and CSF metabolomic analyses.

(2) Compensatory metabolic adaptations

To further determine whether ATP13A4 loss impacts polyamine homeostasis beyond transport alone, we examined the expression of key enzymes involved in polyamine metabolism in both whole-brain lysates and primary astrocytes derived from WT and *Atp13a4* KO mice (**Fig. 5i-l, Supplementary Fig. 12a-p**). In brain tissue, ODC1 (ornithine decarboxylase 1), the rate-limiting enzyme of polyamine synthesis, was significantly upregulated in KO mice, whereas SRM (spermidine synthase) and SMS (spermine synthase) levels remained unchanged. Among catabolic enzymes, SAT1 (spermidine/spermine N^1-acetyltransferase 1) displayed variable expression with one apparent outlier, resulting in no significant difference between genotypes, while SMOX (spermine oxidase) was significantly reduced. In primary astrocytes, a broadly comparable pattern was observed, with increased ODC1 and reduced SMOX expression. SRM expression was significantly increased, whereas SMS and SAT1 remained unchanged. These data indicate that the metabolic response to ATP13A4 loss is largely conserved, with minor cell-type-specific differences. This profile, characterized by increased biosynthetic capacity and reduced catabolic turnover, is consistent with a compensatory response to reduced intracellular polyamine availability. **Together with the metabolomic data, these findings support a model in which impaired ATP13A4-mediated uptake leads to intracellular depletion of polyamines in astrocytes, triggering adaptive metabolic reprogramming.**

Importantly, these data demonstrate that loss of ATP13A4, despite its restricted glial expression pattern and low abundance, not only alters polyamine distribution across compartments but also induces secondary changes in polyamine metabolism. This integrated response underscores the importance of astrocyte-mediated polyamine transport in maintaining polyamine balance in the brain and highlights the *Atp13a4* KO mouse as a useful model to study how glial disturbances in polyamine handling affect brain metabolism. We have incorporated both the metabolomic and biochemical analyses into the main figures of the revised manuscript to emphasize these central findings.

4) Also regarding 3d and e: if the value of WT ACM actually equals that of non-conditioned medium, it would alleviate the discrepancy in concentration between 3d and 3e.

We thank the reviewer for this helpful suggestion. To address this point, we quantified spermidine in non-conditioned medium (NCM) alongside ACM (see also response to **Reviewer 2, Q2**; **Fig. 4d**). Spermidine was not detectable in NCM under our experimental conditions, indicating that the values observed in ACM do not reflect baseline medium levels. Thus, the WT ACM signal does not correspond to background medium but represents astrocyte-derived extracellular spermidine.

While this comparison clarifies the origin of the signal in **Fig. 4d**, it does not fully account for the apparent difference in concentration between **Fig. 4d** and **Fig. 4e**. We interpret this discrepancy as reflecting differences in experimental context: LC-MS/MS of ACM measures the total extracellular spermidine accumulated over time, whereas **Fig. 4e** assesses the biological response to defined exogenous spermidine concentrations. In addition, LC-MS/MS quantifies total recoverable spermidine and does not distinguish between freely diffusible and bound extracellular fractions, which may further contribute to differences between measured and biologically effective concentrations. Accordingly, ACM measurements should not be interpreted as a direct proxy for the effective concentration experienced by neurons in the synaptogenesis assay.

5) Fig S5, astrocyte morphology study and synapse formation: the fact that signal changes are observed with as little as 10 nM polyamine is notable and should be emphasized for its physiological relevance (but also because this experiment is a longer treatment time in the absence of aminoguanidine or SFM; heat inactivation does not eliminate BSAO activity).

We thank the reviewer for highlighting the physiological relevance of the observed effects at low polyamine concentrations. We agree that the pronounced changes in astrocyte morphology and synapse formation at concentrations as low as 10 nM underscore the high sensitivity of astrocytes to extracellular polyamine levels. Notably, these concentrations overlap with the lower range of reported extracellular polyamine levels in the brain, supporting the physiological relevance of these findings. This point has now been emphasized more clearly in the revised manuscript.

Regarding the experimental conditions, these assays were performed in serum-free medium. While this minimizes the contribution of serum-derived amine oxidases, we acknowledge that the absence of serum and trophic factors may influence astrocyte responsiveness and could enhance sensitivity to exogenously applied polyamines.

Minor suggestions:

6) Figure 1c: msAtp13a4 punctae are not visible; please provide a more convincing image Lines 150-151 state that msAtp13a4 is enriched within GFP-positive astrocytes, but it is unclear to what this enrichment is compared to. Were punctae also measured in neurons?

We thank the Reviewer for this valuable comment. We acknowledge that the *msAtp13a4* puncta were not clearly visible in the originally submitted image. The apparent signal weakness was accentuated by a distortion introduced during file conversion, which reduced image clarity. This issue has now been corrected in the revised figure (**Fig. 1c**), which now includes enlarged, high-resolution panels spanning three developmental timepoints (P7, P15, and P21) and a magnified view at P21 to more clearly demonstrate individual *msAtp13a4* mRNA puncta (scale bar, 3 μm). We apologize for the confusion.

In the revised manuscript, we have clarified that the term "enriched" refers to the more prominent *msAtp13a4* punctate signal observed in GFP-labeled astrocytes relative to neighboring GFP-negative cells, which include neurons. While we did not systematically quantify puncta in neurons, our imaging consistently supports a relative enrichment of *msAtp13a4* in astrocytes, as further supported by the quantification shown in **Fig. 1d**, which demonstrates that the majority of *msAtp13a4* puncta consistently overlap with Aldh1l1-GFP-positive astrocytes across all cortical layers and timepoints examined. The text has been revised accordingly to avoid implying absolute cell-type specificity.

7) Fig. 1j: This schematic is very useful but could be improved by defining the orange and green dots in the figure legend.

We thank the reviewer for this helpful suggestion. We have updated the figure legend to explicitly define the color coding.

8) Are the sex-specific effects observed in mice also noted in human patients?

We thank the reviewer for this question. To date, no sex-specific differences have been reported among human carriers of ATP13A4 variants. Published cases include both males and females carrying disruptive or missense variants associated with language impairment, autism spectrum disorder, intellectual disability, or epilepsy. These include the chromosomal inversion described by Kwasnicka-Crawford et al (24), affecting a father and daughter, as well as missense variants such as p.Ala356Val (sex not specified), p.Glu646Asp (sex not specified) (24-26) and p.Glu276Lys (female), and truncating variants including p.Gln273* (male) (27) and the exon 19–25 deletion (female) described in our study.

Although the sex of several affected individuals is not specified, no consistent difference in variant type, frequency, or clinical severity between males and females have been reported to date. Importantly, the total number of documented patients worldwide remains very small, which currently precludes meaningful conclusions regarding potential sex-dependent effects in humans. We have therefore clarified in the revised Discussion that, although we report sex-dependent phenotypic differences in adult *Atp13a4* KO mice, the currently available clinical data remain too limited to determine whether similar sex-dependent effects occur in human patients. We further clarify that the *Atp13a4* KO mice exhibit differences in developmental milestones that are sex-independent, suggesting that the mild sex-dependent differences at older age may possibly reflect compensatory adaptations in brain circuitry rather than intrinsic sex-dependent consequences of ATP13A4 deficiency.

9) The idea that extracellular spermidine is part of the pathology is very interesting. Can it be measured in vivo? Could it be detected in CSF? Is there increased spermidine in peripheral blood of the mouse model?

We thank the reviewer for this insightful suggestion. To assess whether ATP13A4 deficiency affects polyamine levels *in vivo*, we performed targeted LC-MS/MS metabolomic analysis of plasma, cortical tissue, and cerebrospinal fluid (CSF) from WT and *Atp13a4* KO mice.

Plasma levels of ornithine, putrescine, spermidine, spermine, and their major acetylated derivatives were comparable between genotypes (**Supplementary Fig. 11a-g**), indicating that circulating polyamines are largely unaffected by ATP13A4 loss. N^1-acetylspermine was below the limit of detection in plasma in both genotypes, consistent with its low abundance in circulation.

In contrast, analysis of brain and CSF samples revealed clear, compartment-specific alterations in polyamine homeostasis. Targeted metabolomic profiling of cortical tissue showed a significant reduction in putrescine and acetylated polyamine derivatives in *Atp13a4* KO mice (**Fig. 5a-d**), indicating depletion of specific intracellular polyamine pools. While these measurements reflect total tissue levels and do not distinguish intra- versus extracellular compartments, complementary analysis of cerebrospinal fluid (CSF) provided insight into extracellular polyamine distribution. Notably, CSF levels of putrescine and $N^{1/8}$-acetylspermidine were significantly increased in *Atp13a4* KO mice (**Fig. 5e-g**), consistent with altered extracellular polyamine accumulation. Although CSF does not directly measure local interstitial or perisynaptic polyamine concentrations, these findings provide *in vivo* evidence for extracellular polyamine redistribution following ATP13A4 loss.

Together, these findings indicate that ATP13A4 deficiency does not alter systemic polyamine levels but instead leads to localized dysregulation of brain polyamine homeostasis, characterized by intracellular depletion and extracellular accumulation. **These new *in vivo* data strengthen the conclusions of our manuscript and provide direct support for our proposed model that ATP13A4-mediated uptake in astrocytes contributes to buffering extracellular polyamine levels.**

Reviewer #3 (Remarks to the Author):

The study identifies ATP13A4, a P5B-type ATPase highly enriched in astrocytes, as the principal transporter that internalizes extracellular polyamines and maintains their homeostasis. ATP13A4 loss in cultured astrocytes or knockout mice reduces astrocytic arbor complexity, heightens excitatory synaptogenesis, and produces mild, sex-specific behavioral phenotypes; human loss-of-function variants produce similar defects, firmly linking disturbed astrocytic polyamine transport to neurodevelopmental pathology. Exogenous spermidine phenocopies the knockout, underscoring astrocytes' pivotal role in clearing excess polyamines to sculpt synapse formation.

This work provides the first mechanistic bridge between glial polyamine handling and excitatory synapse development, spotlighting astrocytes (not neurons) as critical regulators of circuit maturation.

By uniting molecular, cellular, animal, and human genetic data, it opens a new therapeutic avenue for neurodevelopmental disorders rooted in disrupted astrocyte physiology. The presented data are comprehensive and largely convincing, and addressing the following points would strengthen the manuscript.

We sincerely thank the reviewer for the insightful and encouraging evaluation of our manuscript. **We greatly appreciate the reviewer's positive summary of our findings and their recognition that this study provides a mechanistic link between glial polyamine handling and excitatory synapse development.** We are grateful for the acknowledgment that our integrative approach – spanning molecular, cellular, animal, and human genetic data – offers novel insight into how polyamine dysregulation in astroglia can influence neurodevelopmental outcomes. Below, we address each of the reviewer's points in detail and describe the corresponding revisions we made to the manuscript.

1. In Figure 1c the msAtp13a4 in-situ hybridization signal is barely discernible. It would be helpful to know whether this faint appearance reflects genuinely low transcript levels in the sampled tissue or instead arises from technical factors such as probe efficiency, imaging settings, or overall image quality.

We thank the reviewer for raising this important point. The relatively faint appearance of the msAtp13a4 RNA-FISH signal reflects a combination of both biological and technical factors. Consistent with publicly available single-cell RNA-seq datasets (**Supplementary Fig. 1-2**), *msAtp13a4* is expressed at relatively low transcript abundance in astrocytes compared with canonical astrocyte markers such as *Gfap* or *Aldh1l1*. Nonetheless, the signal detected by RNA-FISH is specific and reproducible across multiple replicates.

We also note that in the originally submitted version, the apparent signal weakness was accentuated by a distortion introduced during file conversion from PowerPoint to PDF, which reduced image clarity. This issue is corrected in the revised figure (**Fig. 1c**), which now includes enlarged, high-resolution panels spanning three developmental timepoints (P7, P15, and P21) and a magnified view at P21 to more clearly demonstrate individual

msAtp13a4 mRNA puncta. The inclusion of multiple independent examples across postnatal development further confirms the reproducibility of the signal. We apologize for the confusion.

Thus, while *Atp13a4* transcripts are indeed of low abundance, the revised images across multiple developmental timepoints and independent expression datasets together confirm that the observed pattern accurately reflects genuine astrocytic expression rather than technical artifact.

2. The TurboID data in Figures 1e–i place ATP13A4 and its paralog ATP13A3 in close proximity to the endo-/lysosomal marker LAMP1, yet the conventional fluorescence images in Supplementary Figure 1 (particularly panels 1a, 1c, and 1f) do not show obvious colocalization between ATP13A4 and LAMP1. A brief explanation that reconciles these observations, perhaps by highlighting methodological sensitivities or temporal differences captured by TurboID, would greatly clarify the interpretation of ATP13A4's subcellular localization.

We thank the reviewer for this helpful observation. We agree that the original LAMP1 immunofluorescence images did not optimally illustrate the co-localization of LAMP1 with ATP13A4. In the revised manuscript, we have replaced these images with improved data that more clearly visualize the LAMP1-positive compartment and its spatial relationship to ATP13A4 (**Supplementary Fig. 3**). This improved image better reflects the proximity suggested by the TurboID data.

Importantly, TurboID-based proximity labeling and conventional immunofluorescence provide complementary information with distinct spatial and temporal resolution. TurboID labels proteins within a ~10 nm radius of the bait over an extended time window, thereby capturing transient interactions and neighboring proteins within shared membrane microdomains or trafficking intermediates. In contrast, immunofluorescence represents a static snapshot, where colocalization can be underestimated due to limited resolution, fixation effects, or the highly dynamic and vesicular nature of the endo-/lysosomal system.

We therefore interpret the strong TurboID enrichment of msAtp13a4 and msAtp13a3 in the Lamp1-positive compartment as evidence for dynamic or transient association with endo-/lysosomal membranes, which may not always be captured as clear overlap in conventional imaging. This interpretation is consistent with previous observations for P5B-type ATPases (28). We have clarified this distinction in the revised manuscript to reconcile the apparent discrepancy between both approaches.

3. Although ATP13A4 shares ~37 % sequence identity with the well-characterized polyamine transporter ATP13A2, supporting the authors' hypothesis that it moves polyamines, this modest similarity also leaves room for additional substrates. It may be helpful to explore whether ATP13A4 can transport other metabolites alongside polyamines.

We thank the reviewer for raising this important point regarding potential alternative substrates. Although ATP13A4 shares only ~37% overall sequence identity with the well-

characterized polyamine transporter ATP13A2, the residues shaping the transmembrane substrate-binding cavity are highly conserved between the two proteins (**Supplementary Fig. 5a**). Structural alignment of the polyamine-binding pockets shows that nearly all spermine-interacting residues are preserved, with only a single non-conservative substitution (ATP13A4 p.Asn930 *versus* ATP13A2 p.Asp955) within the cavity. While the overall architecture of the binding site remains strongly conserved, supporting a shared core specificity for polyamines, this localized difference may subtly alter charge or steric constraints and could therefore contribute to the broader polyamine specificity observed for ATP13A4.

To directly address the possibility of additional substrates, we expanded our ATPase substrate screen to include diamines and acetylated polyamine derivatives. As shown in the revised manuscript (**Supplementary Fig. 5h**), ATP13A4 ATPase activity is robustly stimulated not only by spermidine and spermine, but also by other polyamine species. In contrast, the polyamine precursor ornithine does not stimulate ATPase activity under the conditions tested. Importantly, although these molecules clearly activate ATP hydrolysis, their dose response curves are consistently right-shifted relative to spermidine, indicating a lower apparent affinity. These data demonstrate that ATP13A4 is not strictly specific for higher-order polyamines but instead recognizes a broader class of structurally related cationic metabolites, with the highest apparent affinity for spermidine and spermine. The lack of stimulation by ornithine further indicates that substrate recognition is not solely determined by positive charge, but also depends on structural features such as chain length, spacing of amine groups, and the absence of a carboxyl moiety.

We have incorporated these new data into the revised manuscript, clarifying that ATP13A4 functions as a polyamine transporter with broad substrate specificity.

4. The current data leave the directionality of ATP13A4-mediated polyamine transport unclear. If ATP13A4 resides at the plasma membrane, it could plausibly account for the polyamine retention noted in Fig. 1p ,q and Supplementary Fig. 1g ,h. Conversely, if the transporter is restricted to lysosomes in astrocytes, the pathway by which it elevates cytosolic—or lowers extracellular—polyamine levels remains obscure. Because this point bears directly on the study's main conclusions, additional evidence is needed to clarify ATP13A4's subcellular localization and transport direction.

We address this important mechanistic question together with the next comment (5), where we provide additional data and clarification on ATP13A4 localization, directionality, and its contribution to endocytic versus transporter-mediated uptake.

5. The proposed two-step model hinges on both endocytic uptake of extracellular polyamines and ATP13A4-mediated release from endosomes/lysosomes into the astrocytic cytosol. Could the authors clarify whether ATP13A4 actively contributes to the initial endocytic process, or functions solely as an intracellular exporter once polyamines are internalized? Clearer information on transport directionality—and a revised version of Fig. 1j to reflect this—would help readers follow the model more easily.

We thank the reviewer for these comments. To address this point, we expanded our analysis to clarify how ATP13A4 contributes to cellular polyamine uptake and to refine its proposed transport mechanism. Our functional data consistently support a model in which ATP13A4 drives net cellular uptake and cytosolic accumulation of extracellular polyamines irrespective of its precise intracellular localization.

(1) Functional uptake and directionality

Across multiple models (H4 neuroglioma, C8-D1A astrocytes, and primary astrocytes), expression of ATP13A4 promotes robust cellular BODIPY-spermidine uptake, while its knockdown or catalytic inactivation markedly reduces internalization (**Fig. 2g-h**; **Supplementary Fig. 6c-e, i-q, s-t**). Thus, these data support the key conclusion that ATP13A4 is essential for the accumulation of extracellular polyamines within cells, irrespective of the precise entry mechanism.

Consistent with its classification as a P5B-type ATPase, ATP13A4 operates through an ATP-dependent, unidirectional transport cycle analogous to ATP13A2 (29-33). Spermidine-induced dephosphorylation of ATP13A4 in our biochemical assays indicates that substrate binding occurs at the luminal-facing E2P state, followed by dephosphorylation and release of polyamines into the cytosol (**Fig. 2a**). This biochemical behavior aligns with the strong conservation of the luminal polyamine binding pocket between ATP13A4 and ATP13A2 (**Supplementary Fig. 5a**). Previously, ATP13A2 cryo-EM structures have elucidated the polyamine translocation path from lumen to cytosol, and a similar mechanism for ATP13A4 can be predicted based on the biochemical properties and sequence conservations. Together, our data support the model that ATP13A4 mediates active, inward polyamine transport from the exoplasmic to cytoplasmic side.

(2) Endocytic contribution

To assess the contribution of endocytosis to polyamine uptake, we performed FACS-based polyamine uptake assays using endocytosis inhibitors and temperature-dependent blockade of endocytosis (**Supplementary Fig. 6f-g**).

In H4 neuroglioma cells overexpressing hATP13A4, we observed strongly increased BODIPY-spermidine uptake, which was modestly reduced (~20%) by endocytosis inhibitors (Dynasore, Genistein, Pitstop 2). In contrast, uptake was more strongly reduced (~50%) by the same inhibitors in cells expressing FLUC or the catalytically inactive hATP13A4 D486N mutant on top of the endogenous ATP13A4, consistent with greater reliance of endogenous ATP13A4 on endocytic internalization. Temperature-dependent uptake assays supported these findings: BODIPY-spermidine uptake was strongly reduced at 4°C in FLUC-expressing or catalytically inactive (D486N) cells, but only partially reduced in WT hATP13A4-overexpressing H4 cells. **Our findings are consistent with an active, transporter-mediated mechanism that largely depends on endocytosis.**

(3) Subcellular localization and transport model

The majority of ATP13A4 has been confined to endosomal compartments. The TurboID approach has placed ATP13A4 within LAMP1-positive astrocytic vesicles. ATP13A4 displays a predominantly punctate intracellular distribution that colocalizes with the endo-/lysosomal markers LAMP1 and CD63 (**Supplementary Fig. 3**), consistent with localization

to the endo-/lysosomal system. However, overexpression of ATP13A4 may also mediate endocytosis-independent polyamine uptake, which may reflect saturation of the endosomal system and presence of ATP13A4 in the plasma membrane due to the overexpression of ATP13A4. Indeed, we noted partial localization of mCherry-ATP13A4 to the plasma-membrane (**Figure R2**). However, we cannot rule out a regulated, dynamic localization between the plasma membrane and the endosomal system, which has been previously reported for ATP13A3 (34). Whether ATP13A4 similarly undergoes dynamic relocalization in response to e.g. changes in extracellular polyamine availability remains an interesting question for future investigation. Unfortunately, localization studies of endogenous ATP13A4 are currently not yet feasible, since validated antibodies that allow the detection of endogenous ATP13A4 via immunocytochemistry are not yet available.

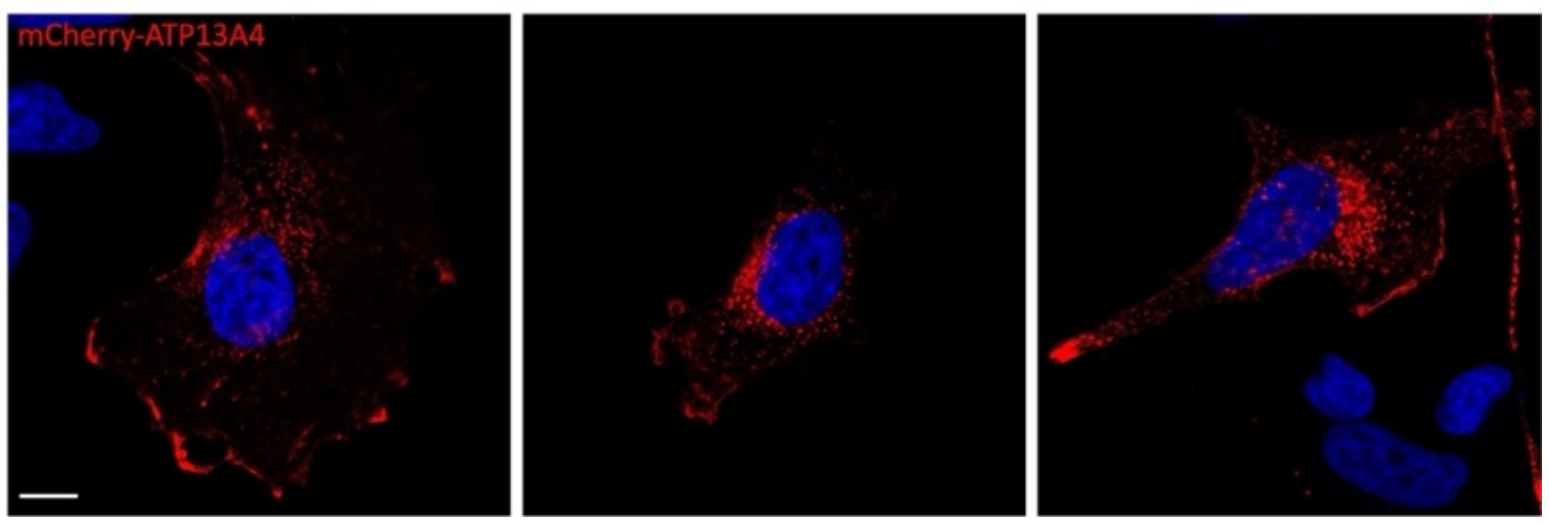

Figure R2. msAtp13a4 displays a predominantly endo-/lysosomal-like punctate distribution with occasional plasma membrane localization. Immunocytochemistry of HeLa cells transiently expressing N-terminal mCherry-labeled msAtp13a4. Scale bar, 10 µm.

Irrespective of the precise localization, ATP13A4 contributes to net cytosolic accumulation and retention of polyamines within cells, which at least largely depends on endocytosis at the endogenous level, which is consistent with the overall uptake phenotype observed across all experimental systems. We clarified this mechanism and its implications in the revised Discussion.

To further clarify this point for the reader, we have revised the legend of **Fig. 2a** to explicitly indicate that the "extracytosolic" side refers broadly to polyamines located outside the cytosol, including both the extracellular space and the lumen of endocytic/endolysosomal compartments. This adjustment avoids implying a single defined entry route and reflects our data, which support ATP13A4-mediated transport into the cytosol without definitively distinguishing whether polyamines are delivered via direct uptake at the plasma membrane or following endocytic internalization. The revised schematic therefore emphasizes transport directionality (exoplasmic to cytosolic) rather than a specific membrane context.

6. The manuscript attributes abnormal astrocyte function to ATP13A4 deficiency, as suggested in Fig. 2h-j. However, astrocyte morphology in Atp13a4 knockout mice is not directly shown. Such data would strengthen the claim that ATP13A4 loss leads to astrocytic dysfunction.

We thank the reviewer for this constructive comment. To address this point, we performed morphological analyses in primary astrocytes derived from *Atp13a4* KO mice (see also our response to **Reviewer 1, Q2**). When co-cultured with WT neurons, *Atp13a4* KO astrocytes exhibited a clear reduction in arborization and morphological complexity (**Fig. 3d-e**), consistent with the phenotype observed in our shRNA-based knockdown experiments.

These data demonstrate that loss of ATP13A4 is sufficient to impair astrocyte morphogenesis and confirm that the observed phenotype is not an artifact of knockdown approaches. To complement these findings *in vivo*, we performed GFAP immunostaining in *Atp13a4* KO brain tissue. KO animals displayed increased GFAP immunoreactivity compared to WT controls (**Fig. 5m-n**), indicative of an altered astrocyte state and consistent with disrupted astrocyte homeostasis. Although GFAP staining does not resolve fine astrocyte morphology, these findings provide complementary *in vivo* evidence for astrocyte alterations following ATP13A4 loss.

Together, the primary astrocyte analyses and *in vivo* GFAP data strengthen the conclusion that ATP13A4 loss leads to astrocytic dysfunction.

7. The authors propose in Figs 3 and 4 that elevated extracellular polyamines (e.g., spermidine) cause synaptic and behavioral abnormalities. To establish causality, the authors should test whether polyamine levels were indeed increased or lowering polyamine levels rescues these phenotypes. This would strengthen the proposed mechanistic link.

We thank the reviewer for this important suggestion and agree that direct quantification of polyamine levels further strengthens the proposed mechanistic link between polyamine dysregulation and the observed synaptic and behavioral phenotypes.

To address the first part of this comment, <u>we performed targeted LC-MS/MS metabolomic analysis of cerebrospinal fluid (CSF) from WT and *Atp13a4* KO mice</u> (**Fig. 5e-g**). These data reveal significantly increased levels of putrescine and $N^{1/8}$-acetylspermidine in *Atp13a4* KO mice. Although CSF does not directly measure local interstitial or perisynaptic polyamine concentrations, these findings provide *in vivo* evidence for altered extracellular polyamine distribution following ATP13A4 loss. Together with our in vitro findings showing that elevated extracellular spermidine impacts astrocyte morphology and excitatory synaptogenesis, **these results support the conclusion that dysregulated extracellular polyamine homeostasis contributes to the observed phenotypes**.

We agree that experimentally manipulating polyamine levels would provide additional mechanistic insight into the causal contribution of altered polyamine homeostasis to the synaptic and behavioral phenotypes observed in *Atp13a4* KO mice. However, establishing such rescue paradigms would require prolonged *in vivo* manipulation of polyamine metabolism combined with extensive molecular, electrophysiological, and behavioral characterization. We therefore focused the present revision on directly quantifying *in vivo* polyamine alterations and defining their relationship to the observed astrocytic and synaptic phenotypes. Nevertheless, the current findings provide a strong framework for future studies aimed at testing whether normalization of polyamine levels can rescue the phenotypes associated with ATP13A4 deficiency.

8. The evidence for an anxiolytic phenotype in female KO mice rests largely on 30-s time-bin analyses, yet the full open-field center-time trace (Fig. 4f) shows only a modest WT–KO difference. Complementary assays such as the elevated plus-maze or

light–dark box would provide a more comprehensive assessment of anxiety-related behavior. Likewise, the PTZ challenge was performed at 25 mg/kg, a dose that may be too low to elicit reliable seizure activity; alternative metrics (e.g., seizure-severity scoring across a broader dose range) could strengthen the conclusions.

We thank the reviewer for these thoughtful and constructive suggestions. We agree that the behavioral phenotypes observed in adult *Atp13a4* KO mice are relatively mild, and we revised the manuscript accordingly to describe these findings with appropriate caution.

(1) Open field and anxiety-related behavior

In the open-field test, the 30-s time-bin analyses revealed altered exploratory dynamics consistent with reduced anxiety-like behavior in female KO mice; however, we agree that the cumulative center-time trace shows only modest genotype differences. To further evaluate this phenotype, we performed elevated plus maze testing (**Figure R3a-d**), which did not reveal significant genotype-dependent effects. Together, these findings indicate that the apparent anxiolytic phenotype is subtle and context-dependent rather than a robust, generalized behavioral change.

(2) Expanded PTZ analysis

For the PTZ assay, the original 25 mg/kg paradigm was selected because lower PTZ doses can be more sensitive for detecting increased seizure susceptibility in predisposed models. To further address the reviewer's concern, we performed an expanded PTZ dose-response analysis in a one-year-old cohort (**Figure R3e**). Across this range, we did not observe consistent genotype-dependent differences in seizure threshold or severity. At higher doses, responses showed substantial inter-individual variability and converged across genotypes. These findings indicate that *Atp13a4* loss does not robustly alter seizure susceptibility under the conditions tested.

In parallel, automated DigiGait analysis revealed altered hindlimb positioning parameters in female *Atp13a4* KO mice, whereas most temporal gait measures remained unchanged (**Fig. 6m-o**). Together with the altered open-field exploratory dynamics and enhanced rotarod performance, these findings support subtle and partially sex-biased alterations in adult motor and exploratory behavior.

(3) Developmental interpretation

To better contextualize the adult behavioral findings, we expanded our analyses to early postnatal development (see response to **Reviewer 1**, **Q9**; **Fig. 6a-k**). We observed consistent delays across multiple postnatal developmental milestones, with eye opening, rooting reflex and forelimb grasp reaching statistical significance after Bonferroni correction. These early phenotypes were observed across the cohort and were largely sex independent, indicating a broad effect of *Atp13a4* loss on early circuit maturation.

Taken together, our data support a model in which *Atp13a4* deficiency primarily impacts early neurodevelopmental processes, leading to alterations in circuit formation that manifest later as mild, context-dependent, and partially sex-specific behavioral phenotypes in adulthood. We have revised the manuscript accordingly to

better reflect this developmental framework and to avoid overinterpretation of the adult behavioral data.

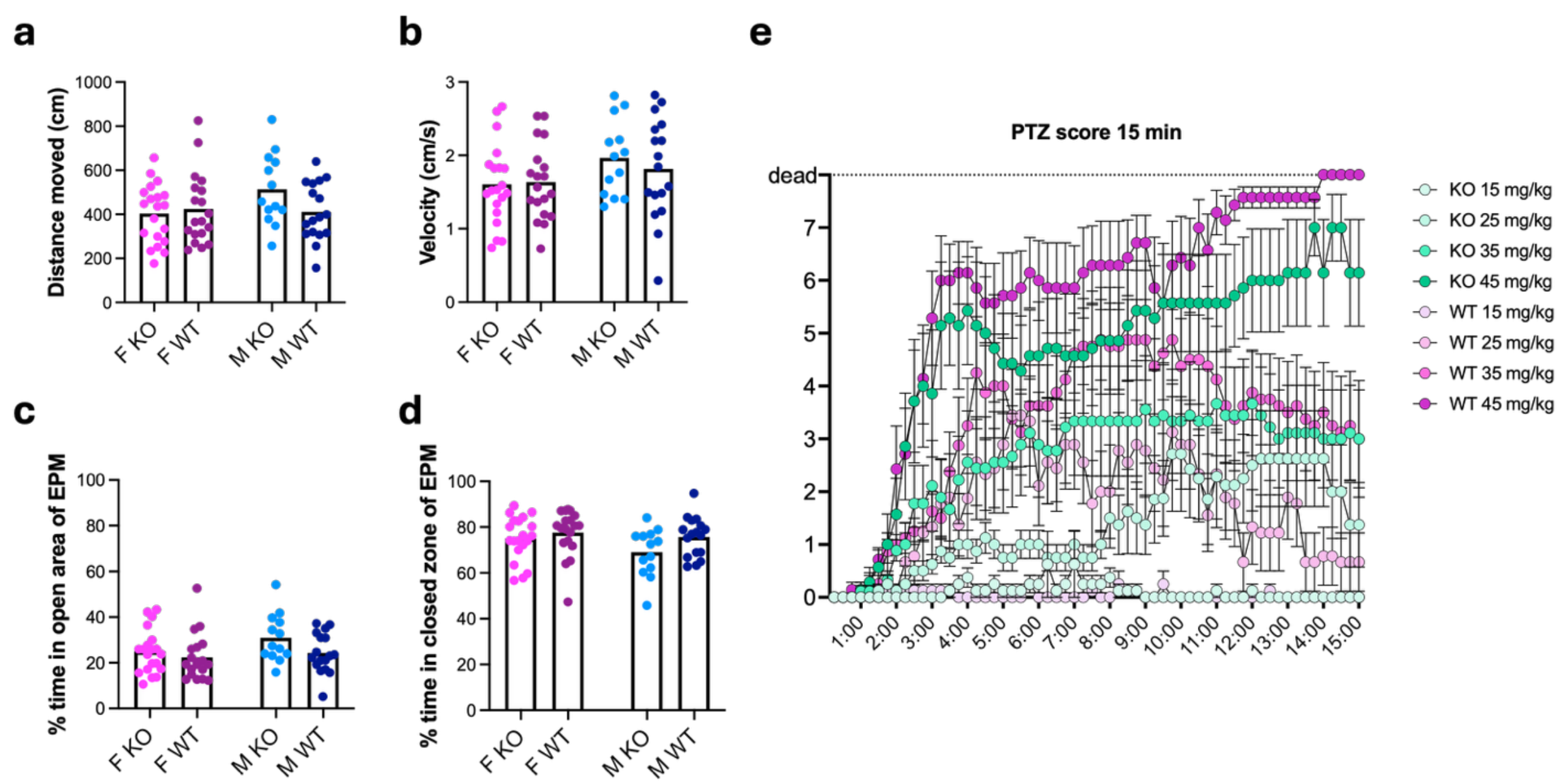

Figure R3. Expanded behavioral assessment of adult *Atp13a4* KO mice. a-d, Elevated plus maze (EPM) behavior in female (F) and male (M) *Atp13a4* KO and WT mice. Distance moved (**a**), velocity (**b**), percentage time spent in open arms (**c**), and percentage time spent in closed arms (**d**) are shown. **e**-**h**, PTZ-induced seizure susceptibility assessed across an expanded dose range (15-45 mg/kg) in 1-year-old mice. Seizure responses are plotted over time, showing substantial inter-individual variability and no consistent genotype-dependent differences in seizure threshold or severity. Each dot represents an individual animal; bars denote mean.

9. The manuscript centers on astrocytic arborization while largely dismissing neuronal morphology, citing "no significant differences in neurite elongation" after ACM treatment (Supplementary Fig. 6a–b) to claim ATP13A4 is dispensable for dendritic growth. This rests on the untested premise that ATP13A4 is exclusive to astrocytes, yet the documented increase in excitatory synapse number and mEPSC frequency suggests neuronal branching is probably altered. Two methodological issues compound the concern: (i) ACM treatment assumes astrocytes are the sole regulators of extracellular polyamines and that spermidine accumulates in the medium; direct polyamine application would provide a clearer test; (ii) all data are in vitro, despite the availability of ATP13A4-KO mice. Demonstrating astrocytic and neuronal morphology in vivo would greatly strengthen the authors' conclusions.

We thank the reviewer for this comment and for raising important points regarding the interpretation of ACM experiments and the relationship between synaptic and neuronal morphology phenotypes.

(1) ACM versus direct polyamine exposure

We agree that ACM-based approaches should be complemented by direct polyamine application. Importantly, both approaches were performed. Direct application of spermidine to primary astrocytes and neurons recapitulated the key phenotypes observed

with ACM, including enhanced astrocyte arborization and increased excitatory synaptogenesis (**Supplementary Fig. 9b-c**, **f-g**; **Fig. 4e-f**). This demonstrates that extracellular spermidine is sufficient to drive these effects independently of other ACM-derived factors. In parallel, we directly quantified spermidine levels in ACM and found a significant increase following *Atp13a4* knockdown (**Fig. 4d**), confirming that ATP13A4-deficient astrocytes alter the extracellular polyamine environment. Together, these data demonstrate that ACM reflects a measurable, ATP13A4-dependent change in extracellular polyamine availability rather than an assumption of accumulation.

(2) Neuronal morphology versus synaptic phenotype

We acknowledge that the increase in excitatory synapse number and mEPSC frequency could suggest accompanying changes in neuronal morphology. However, under our experimental conditions, neither ACM (**Supplementary Fig. 10a-b**) nor direct spermidine application (**Supplementary Fig. 10m-n**) induced significant changes in dendrite elongation, despite both conditions robustly increasing excitatory synapse number (**Fig. 4e-f**). This dissociation indicates that ATP13A4-dependent polyamine dysregulation does not promote global dendritic outgrowth but instead selectively enhances synaptic connectivity. Our data therefore support a model in which extracellular polyamines primarily modulate synapse formation and function rather than large-scale neuronal morphology. This is consistent with established roles of polyamines in regulating ionotropic glutamate receptors, synaptic protein translation, and spine dynamics, processes that can increase synapse number and efficacy without altering overall neurite length.

(3) Cell-type specificity

We have modified the manuscript to now describe ATP13A4 as glia-enriched, with predominant expression in astrocytes in the mouse brain. Our conclusions therefore rely on the dominant role of astrocytes in extracellular polyamine buffering rather than absolute cell-type specificity. Future studies using cell type–specific animal models will be required to further delineate the relative contributions of astrocytic versus non-astrocytic ATP13A4 to the observed phenotypes.

(4) *In vivo* validation

As described in our responses to **Reviewer 1** (**Q2**) and **Reviewer 3** (**Q6**), we now include analyses of *Atp13a4* KO models showing altered astrocyte morphology and reactivity (**Fig. 3d-e**, **Fig. 5m-n**). In addition, our *in vivo* data demonstrate compartment-specific changes in polyamine homeostasis, including extracellular accumulation in CSF and intracellular depletion in brain tissue (**Fig. 5a-g**). In addition, we observe increased excitatory synapse number and synaptic activity *in vivo* (**Fig. 5o-p**), consistent with our *in vitro* findings (**Fig. 4**).

We did not pursue additional *in vivo* neuronal morphology analyses, as our *in vitro* data consistently show that neither ACM nor direct spermidine exposure alters dendrite elongation despite effects on synapse number. These findings indicate that ATP13A4-dependent polyamine dysregulation primarily impacts synaptic connectivity rather than global neuronal morphology and therefore suggest that major alterations in dendritic architecture are unlikely to represent the primary phenotype *in vivo*.

Together, these findings provide *in vivo* support for our model that ATP13A4-dependent astrocytic uptake regulates extracellular polyamine levels, which in turn modulate excitatory synaptic development.

Reference list

1. Bakken TE, van Velthoven CT, Menon V, Hodge RD, Yao Z, Nguyen TN, et al. Single-cell and single-nucleus RNA-seq uncovers shared and distinct axes of variation in dorsal LGN neurons in mice, non-human primates, and humans. Elife. 2021;10.
2. Jorstad NL, Close J, Johansen N, Yanny AM, Barkan ER, Travaglini KJ, et al. Transcriptomic cytoarchitecture reveals principles of human neocortex organization. Science. 2023;382(6667):eadf6812.
3. Siletti K, Hodge R, Mossi Albiach A, Lee KW, Ding SL, Hu L, et al. Transcriptomic diversity of cell types across the adult human brain. Science. 2023;382(6667):eadd7046.
4. Velmeshev D, Perez Y, Yan Z, Valencia JE, Castaneda-Castellanos DR, Wang L, et al. Single-cell analysis of prenatal and postnatal human cortical development. Science. 2023;382(6667):eadf0834.
5. Zhang Y, Sloan SA, Clarke LE, Caneda C, Plaza CA, Blumenthal PD, et al. Purification and Characterization of Progenitor and Mature Human Astrocytes Reveals Transcriptional and Functional Differences with Mouse. Neuron. 2016;89(1):37-53.
6. Saunders A, Macosko EZ, Wysoker A, Goldman M, Krienen FM, de Rivera H, et al. Molecular Diversity and Specializations among the Cells of the Adult Mouse Brain. Cell. 2018;174(4):1015-30 e16.
7. Tabula Muris C, Overall c, Logistical c, Organ c, processing, Library p, et al. Single-cell transcriptomics of 20 mouse organs creates a Tabula Muris. Nature. 2018;562(7727):367-72.
8. Zeisel A, Hochgerner H, Lonnerberg P, Johnsson A, Memic F, van der Zwan J, et al. Molecular Architecture of the Mouse Nervous System. Cell. 2018;174(4):999-1014 e22.
9. Zhang Y, Chen K, Sloan SA, Bennett ML, Scholze AR, O'Keeffe S, et al. An RNA-sequencing transcriptome and splicing database of glia, neurons, and vascular cells of the cerebral cortex. J Neurosci. 2014;34(36):11929-47.
10. Skatchkov SN, Woodbury-Farina MA, Eaton M. The role of glia in stress: polyamines and brain disorders. Psychiatr Clin North Am. 2014;37(4):653-78.
11. Laube G, Veh RW. Astrocytes, not neurons, show most prominent staining for spermidine/spermine-like immunoreactivity in adult rat brain. Glia. 1997;19(2):171-9.
12. Krauss M, Langnaese K, Richter K, Brunk I, Wieske M, Ahnert-Hilger G, et al. Spermidine synthase is prominently expressed in the striatal patch compartment and in putative interneurones of the matrix compartment. J Neurochem. 2006;97(1):174-89.
13. Krauss M, Weiss T, Langnaese K, Richter K, Kowski A, Veh RW, et al. Cellular and subcellular rat brain spermidine synthase expression patterns suggest region-specific roles for polyamines, including cerebellar pre-synaptic function. J Neurochem. 2007;103(2):679-93.
14. Bernstein HG, Muller M. The cellular localization of the L-ornithine decarboxylase/polyamine system in normal and diseased central nervous systems. Prog Neurobiol. 1999;57(5):485-505.
15. Guerra GP, Rubin MA, Mello CF. Modulation of learning and memory by natural polyamines. Pharmacol Res. 2016;112:99-118.
16. Biedermann B, Skatchkov SN, Brunk I, Bringmann A, Pannicke T, Bernstein HG, et al. Spermine/spermidine is expressed by retinal glial (Muller) cells and controls distinct K+ channels of their membrane. Glia. 1998;23(3):209-20.
17. Williams K. Interactions of polyamines with ion channels. Biochem J. 1997;325 (Pt 2)(Pt 2):289-97.
18. Chen W, Harnett MT, Smith SM. Modulation of neuronal voltage-activated calcium and sodium channels by polyamines and pH. Channels (Austin). 2007;1(4):281-90.

19. Cavalli P, Raffauf A, Passarella S, Helmuth M, Dieterich DC, Landgraf P. Manipulation of DHPS activity affects dendritic morphology and expression of synaptic proteins in primary rat cortical neurons. Front Cell Neurosci. 2024;18:1465011.
20. Padgett LR, Shinkle MR, Rosario S, Stewart TM, Foley JR, Casero RA, Jr., et al. Deoxyhypusine synthase mutations alter the post-translational modification of eukaryotic initiation factor 5A resulting in impaired human and mouse neural homeostasis. HGG Adv. 2023;4(3):100206.
21. Kim HK, Kim JH, Kim YM, Kim GH, Lee BH, Choi JH, et al. Lowe syndrome: a single center's experience in Korea. Korean J Pediatr. 2014;57(3):140-8.
22. Wang W, Frankel WN. Overlaps, gaps, and complexities of mouse models of Developmental and Epileptic Encephalopathy. Neurobiol Dis. 2021;148:105220.
23. Lena I, Mantegazza M. Na(V)1.2 haploinsufficiency in Scn2a knock-out mice causes an autistic-like phenotype attenuated with age. Sci Rep. 2019;9(1):12886.
24. Kwasnicka-Crawford DA, Carson AR, Roberts W, Summers AM, Rehnstrom K, Jarvela I, et al. Characterization of a novel cation transporter ATPase gene (ATP13A4) interrupted by 3q25-q29 inversion in an individual with language delay. Genomics. 2005;86(2):182-94.
25. Auranen M, Varilo T, Alen R, Vanhala R, Ayers K, Kempas E, et al. Evidence for allelic association on chromosome 3q25-27 in families with autism spectrum disorders originating from a subisolate of Finland. Mol Psychiatry. 2003;8(10):879-84.
26. Worthey EA, Raca G, Laffin JJ, Wilk BM, Harris JM, Jakielski KJ, et al. Whole-exome sequencing supports genetic heterogeneity in childhood apraxia of speech. J Neurodev Disord. 2013;5(1):29.
27. Hong S, Wang L, Zhao D, Zhang Y, Chen Y, Tan J, et al. Clinical utility in infants with suspected monogenic conditions through next-generation sequencing. Mol Genet Genomic Med. 2019;7(6):e684.
28. Hamouda NN, Van den Haute C, Vanhoutte R, Sannerud R, Azfar M, Mayer R, et al. ATP13A3 is a major component of the enigmatic mammalian polyamine transport system. J Biol Chem. 2021;296:100182.
29. Chen X, Zhou M, Zhang S, Yin J, Zhang P, Xuan X, et al. Cryo-EM structures and transport mechanism of human P5B type ATPase ATP13A2. Cell Discov. 2021;7(1):106.
30. Mu J, Xue C, Fu L, Yu Z, Nie M, Wu M, et al. Conformational cycle of human polyamine transporter ATP13A2. Nat Commun. 2023;14(1):1978.
31. Sim SI, von Bulow S, Hummer G, Park E. Structural basis of polyamine transport by human ATP13A2 (PARK9). Mol Cell. 2021;81(22):4635-49 e8.
32. Tillinghast J, Drury S, Bowser D, Benn A, Lee KPK. Structural mechanisms for gating and ion selectivity of the human polyamine transporter ATP13A2. Mol Cell. 2021;81(22):4650-62 e4.
33. Tomita A, Daiho T, Kusakizako T, Yamashita K, Ogasawara S, Murata T, et al. Cryo-EM reveals mechanistic insights into lipid-facilitated polyamine export by human ATP13A2. Mol Cell. 2021;81(23):4799-809 e5.
34. Sekhar V, Andl T, Phanstiel Ot. ATP13A3 facilitates polyamine transport in human pancreatic cancer cells. Sci Rep. 2022;12(1):4045.

We sincerely thank the reviewers for their careful evaluation of our revised manuscript and for their positive and encouraging comments. We are grateful for their constructive feedback throughout the review process, which has substantially strengthened the manuscript. We are pleased that Reviewers #2 and #3 consider their concerns fully addressed and have no further comments. In response to Reviewer #1's final suggestion, we have incorporated the requested rescue experiment into the manuscript and provide our detailed response below.

Reviewer #1: The authors have done an excellent job in responding to my critiques. I only have one further suggestion. The authors should consider placing Reviewer Figure 1, which shows that the pathogenic variants do not rescue morphological defects, in the main paper. It is a nice finding that reinforces, at the cellular level, that the variant functions as a loss of function.

We thank the reviewer for the positive assessment of our revised manuscript and for this helpful suggestion. We agree that the rescue experiment provides additional support for the functional impairment of disease-associated ATP13A4 variants. To address this comment, we have incorporated these data into the manuscript and now describe the experiment in the Results section. Briefly, re-expression of WT hATP13A4 restored morphological complexity in Atp13a4 KO astrocytes, whereas the disease-associated C-terminal deletion variant (Δexon19-25) failed to do so (**Supplementary Fig. 9**). As this experiment serves primarily as supportive validation of the biochemical and cellular functional analyses already presented in **Fig. 2**, we elected to include it as a supplementary figure to maintain the focus and flow of the main manuscript. We thank the reviewer for highlighting the value of these data.

Reviewer #2: The revised version of this manuscript appropriately addressed my concerns. I recommend acceptance for publication.

We thank the reviewer for the careful evaluation of our manuscript and for the positive assessment of the revised version. We appreciate the reviewer's constructive comments throughout the review process, which have helped strengthen the manuscript.

Reviewer #3: The authors substantially revised their manuscript to address my review comments by incorporating new in vivo metabolomic data, primary knockout astrocyte analyses, and comprehensive developmental behavioral assessments to corroborate the mechanistic link between ATP13A4-dependent astrocytic polyamine regulation and excitatory synapse development. I have no additional comments.

We sincerely thank the reviewer for the thoughtful evaluation of our work and for recognizing the extensive revisions made to strengthen the manuscript. We appreciate the reviewer's constructive feedback throughout the review process, which helped improve the rigor and clarity of the study.
